# No pervasive relationship between species size and local abundance trends

**DOI:** 10.1038/s41559-021-01624-8

**Published:** 2021-12-30

**Authors:** J. Christopher D. Terry, Jacob D. O’Sullivan, Axel G. Rossberg

**Affiliations:** School of Biological and Behavioural Sciences, Queen Mary University of London, London, UK

**Keywords:** Climate-change ecology, Population dynamics, Macroecology, Conservation biology, Community ecology

## Abstract

Although there is some evidence that larger species could be more prone to population declines, the potential role of size traits in determining changes in community composition has been underexplored in global-scale analyses. Here, we combine a large cross-taxon assemblage time series database (BioTIME) with multiple trait databases to show that there is no clear correlation within communities between size traits and changes in abundance over time, suggesting that there is no consistent tendency for larger species to be doing proportionally better or worse than smaller species at local scales.

## Abstract (web summary)

Despite expectations that global anthropogenic pressures on species with communities may be size biased, this relationship has not been tested on a large scale. Here the authors use existing databases to show that larger species have not experienced more declines in abundance within their respective communities than small species.

## Main

Recent analyses have found that, despite high and increasing levels of community turnover, there is no clear overall trend in local-scale species richness^1–4^. However, it remains unclear how this result translates into functional changes. One of the most fundamental functional traits of a species is its size^5,6^ and there is an expectation that a warming climate will lead to a shift towards smaller species^7–11^, drawing upon metabolic theory^12^ and the observed distributional patterns described by Bergmann’s rule^13,14^. Temperature-driven shifts towards smaller species have been observed in tundra plant communities^15^ and some^7,9,16^, but not all^11^, aquatic systems. Furthermore, larger species have been more extinction prone during some previous mass extinctions^17,18^ and are more likely to show strong recent population declines^19^. Although relationships are threat dependent^20,21^, larger species tend to be assessed at a higher risk of extinction due to longer generational intervals and increased threat from habitat loss, fragmentation and hunting^22^.

One might therefore expect a detectable signal of shifts in community trait values beneath the apparent underlying consistency in taxonomic diversity. To examine this, we tested whether the size of a species is correlated with the change in abundance through time using the publicly available BioTIME database^23^. This database is the largest collection of time series of ecological communities and, despite considerable biases that we discuss below, has wide geographic and taxonomic scope^23^. It consists of ‘studies’ defined by a consistent sampling methodology and taxonomic focus. After cleaning and standardizing the names associated with the records, we linked six fundamental ‘size’ traits from four openly accessible trait databases representing four broad guilds: adult body mass from a database of amniote life history traits^24^, adult body length and qualitative body size of marine species from the World Register of Marine Species (WoRMS) database^25^, plant maximum height and seed mass from the TRY database^26^ and maximum body length of fish from a compilation^27^ based on data in the FishBase repository^28^.

**Fig. 1:**
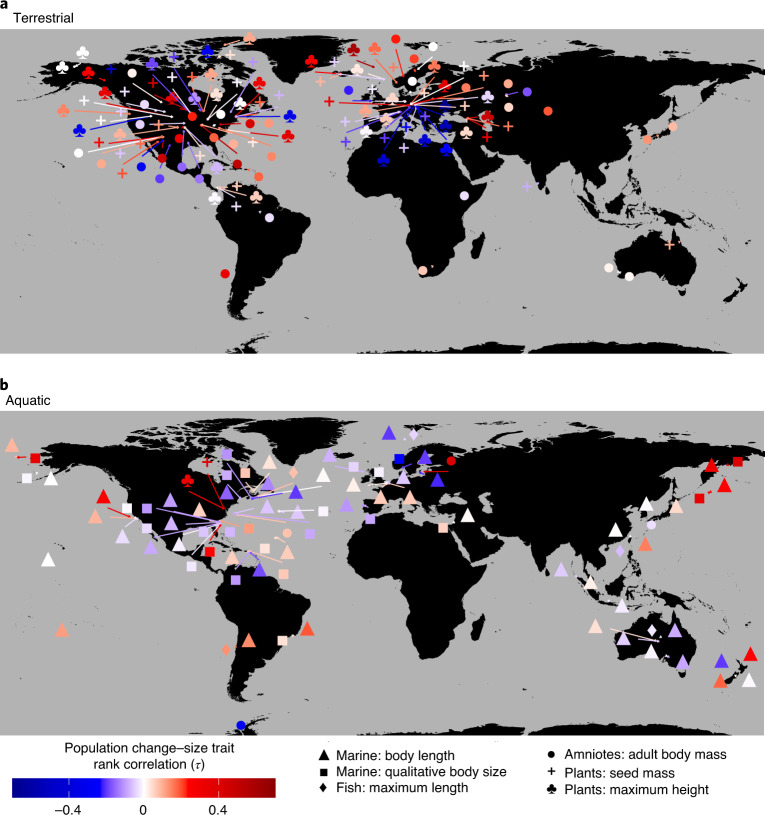
Global distribution of studies in our dataset, showing average *τ* for each study–trait combination and divided into aquatic and terrestrial realms. The aquatic realm is principally marine but includes three freshwater studies. Note that the locations are shown as the centre point of each study, which can cause oceanic studies to be ‘located’ on land. See Extended Data Fig. 1 for full details of study-level results.

Observations from single-location studies were combined, whilst widely dispersed studies were separately binned into a global grid of cells, each approximately 10 km wide, and data from each study and cell were treated as discrete assemblages, following previous analyses^1,29^. Selecting only assemblages with quantitative observations of ≥10 species, over ≥5 years and with ≥40% of the species having records for at least one size trait, we generated 12,956 assemblage time series from 144 studies (Fig. 1). This filtered dataset represented 2,109,593 observations of 10,286 species, of which 7,234 could be linked to at least one size trait (representing 84.02% of observations). Equally weighting studies, the average time series length was 18.2 years (range 5–71.8 years), and the average number of species per included assemblage was 65.4 (range 10–337). The log_10_ ratio between the largest and smallest species in each study averaged 2.49 (range 0.55–6.73) across the ‘mass’ traits and 1.06 (range 0.3–3.15) across the ‘length’ traits.

For each trait and community assemblage time series for which there were sufficient data, we first square-root transformed and standardized each time series following previous approaches^3^ and calculated *β*_*i*_, the slope of a regression of abundance of species *i* against time. We then calculated, for each assemblage, *τ* (the Kendall rank correlation coefficient between the trait in question) and *β*, across the species for which we had trait data. This gives a non-parametric measure of whether larger species are more or less likely than smaller species to have increased through time and, importantly, can be calculated where trait values for only a fraction of the observed species are available. To weight each study within BioTIME equally, where there were multiple assemblages per study, these were averaged to generate a *τ* value for each possible study–trait combination. To provide a reference distribution against which to evaluate the statistical significance of this multistage analysis, we repeated the procedure with 10,000 trait randomizations within each assemblage.

**Fig. 2:**
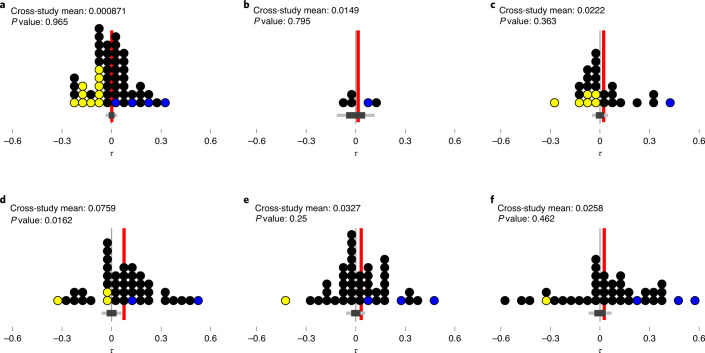
Correlation between six body-size traits and changes in abundance through time (*τ*). **a**–**f**, Distribution of Kendall rank correlation coefficient between body-size traits for body length (**a**) and qualitative body size (**b**) of marine species, maximum length of fish (**c**), adult body mass of amniotes (**d**) and seed mass (**e**) and maximum height (**f**) of plants versus changes in abundance through time. Each dot represents one study, averaging across the constituent assembly time series for studies of large spatial extent. Study-level results are binned into classes 0.05 units of *τ* wide. Coloured dots highlight studies that were individually identified as showing a significant trend (yellow for negative, blue for positive; see Extended Data Fig. 1 for study-level intervals). The error bar below each plot displays the distribution (central 95% and 66%) of mean *τ* values over 10,000 permutations of the size trait data, whilst the red line indicates the observed mean *τ* value within that panel. Displayed *P* values are calculated from permutation tests. Equivalent results using alternative approaches to transforming the community data are given in Extended Data Fig. 3.

**Extended Data Fig. 1:**
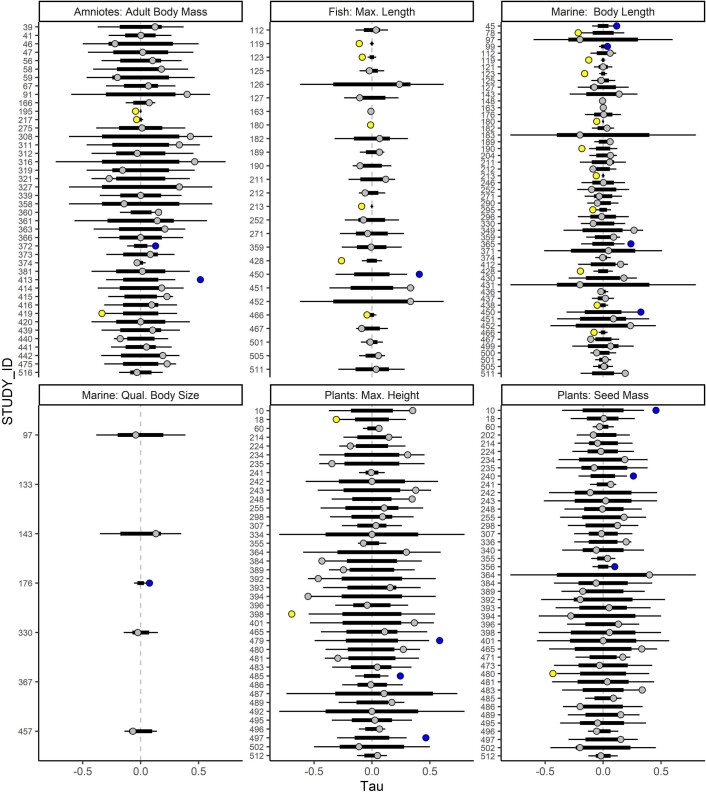
Significance of trait-trend correlation (τ) for each trait-study combination. Within each study, the trait values (including NAs) were randomised 10000 times, and the Kendall rank-correlation recalculated to generate a reference distribution. Black lines show 95% and 66% intervals, dots show observed values. Individual studies were coloured if τ fell outside the central 95%.

**Extended Data Fig. 2:**
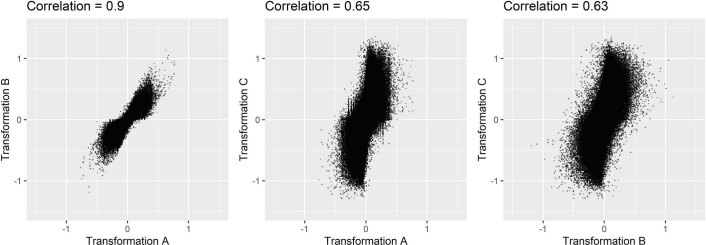
Strong correlation between all trend slopes (β‘s) calculated using alternative population data transformations. Transformation A is that presented in the main text results, Transformation B is the standardisation by dividing by the mean population values, and Transformation C is the rank based approach. Correlations shown are Pearson’s correlation coefficients.

**Extended Data Fig. 3:**
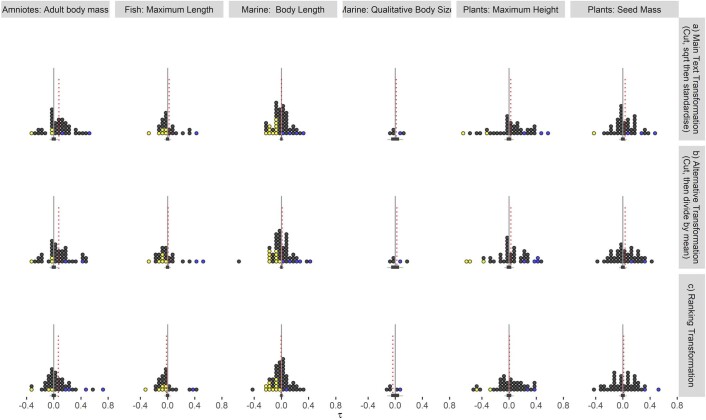
Strong concordance of overall results under different data transformations. Figure elements are the same as in the main text Fig. 2. The red dotted line shows the mean τ value in each facet. All three transformations show the same pattern of trait-level significance - the only guild where a significant (positive) deviation from the null distribution (bar under histograms) is detectable is the amniotes.

**Extended Data Fig. 4:**
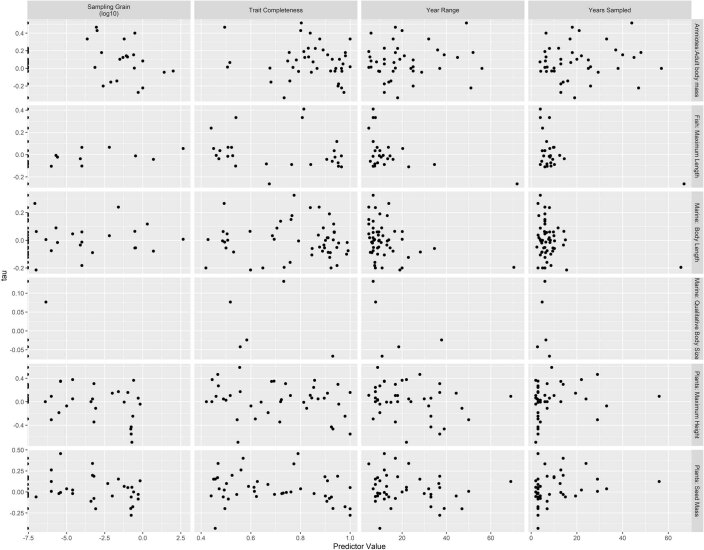
No consistent relationships between the suite of study-level predictors and the principal response variable τ. Predictors (left-right): sampling grain (as listed in BioTIME metadata, the fraction of species in the study that had trait data, the range of years from the start to the end of the study, and the total number years for which there was sample data. Further possible predictors are shown in Extended Data Fig. 5. Statistical results are given in Supplementary Tables 1 and 2. Results are facetted by trait.

**Extended Data Fig. 5:**
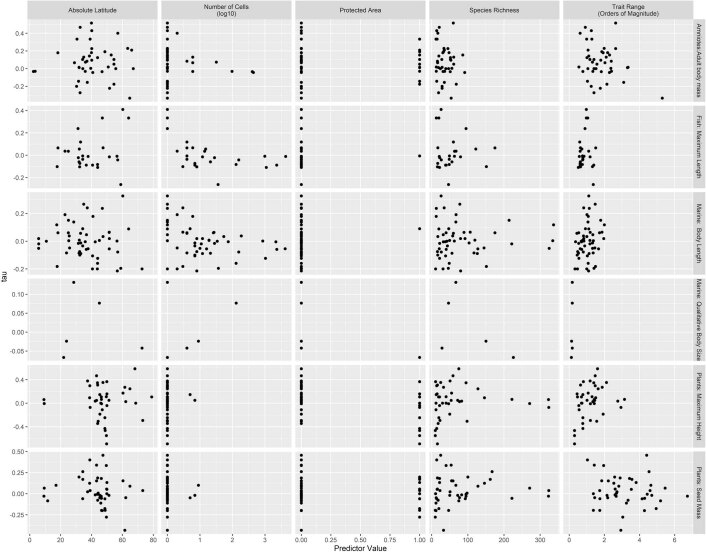
No consistent relationships between the suite of study-level predictors and the principal response variable τ (continued). Predictors (left-right): absolute latitude of study as listed in BioTIME metadata, number of spatially binned assemblages the study was divided into, whether the site was a protected area as listed in BioTIME metadata, the total number of species in the assemblage, and the size difference between the largest and smallest species in the assemblage. Statistical results are given in Supplementary Tables 1 and 2. Results are facetted by trait.

**Extended Data Fig. 6:**
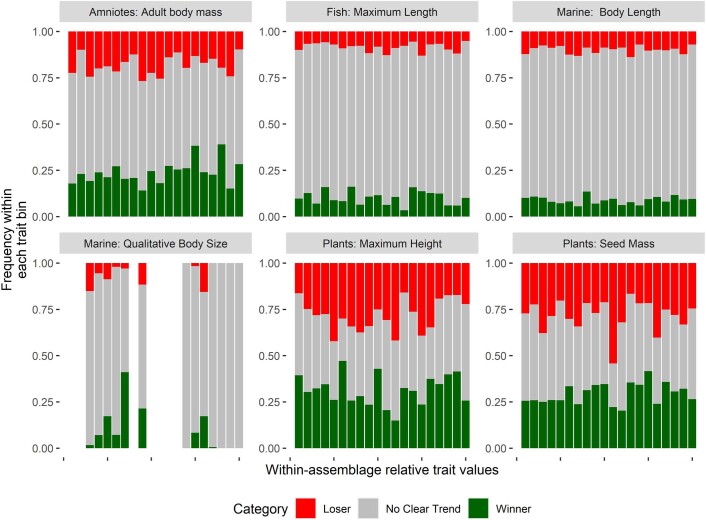
Species-level analysis of population trends. Colours show proportion of species in each relative size bin that are identifiable as a ‘winner’ or ‘loser’ based on the sign and significance (p < 0.1) of a regression line of the transformed abundance against time. Compared to the analysis in the main text (which effectively weights each study equally), this analysis weights each species in each study equally. Species from across the whole dataset are binned along the x-axis by their relative trait value within their assemblage. Because the ‘Qualitative Body Size’ trait has only a limited number of categories, some relative trait ranks did not occur. Where a species is observed in multiple assemblages within a study, the contribution of each time series is downweighted so that each species contributes equally for each study it is in. Statistical summaries are given in Supplementary Tables 3 and 4.

Certain individual studies showed significant relationships between size traits and population trends (coloured dots in Fig. 2 and Extended Data Fig. 1). However, for five of the six tested size traits, the overall mean *τ* values did not differ significantly from the null model (Fig. 2). For one trait (amniote body mass, Fig. 2d) we found a marginally significant (unadjusted for multiple comparisons) overall average positive relationship between size and the slope of population trends (*β*). Alternative population data transformations gave highly concordant results (Extended Data Figs. 2 and 3). Possible confounding factors for the value of *τ* associated with each study, namely the total span of the time series, the number of sample points, the species richness, the range of traits in the assemblage, the average size trait completeness, the number of assemblages within the study, the grain of the study and the absolute latitude, did not consistently predict either *τ* or *τ*^*2*^ (Extended Data Figs. 4 and 5 and Supplementary Tables 1 and 2). Further, the likelihood of an individual species showing either a statistically significant positive or negative population trend was not linked to its relative size trait value within the assemblage (all *P* > 0.05; Extended Data Fig. 6 and Supplementary Table 3).

These results indicate that there is not yet evidence for widely pervasive within-assemblage trends in a core functional trait, size. Importantly however, this study should not be seen as a refutation or diminishment of the heightened threats faced by the very largest apex species^30,31^, which constitute only a minor component of the BioTIME database. Rather, against a background of considerable turnover^2,3^ across whole observed community assemblages, on average, species positions in communities are being taken up by species of comparable size. Our results suggest that previously identified shifts towards smaller species found in some aquatic systems^9,16^ may not be as universal as currently expected^7,11^ and align with the divergent changes in global body-size abundance distributions observed between mammal guilds^32^ and the apparent stability of trait diversity in North American birds despite declines in abundance^33^.

The tendency towards an overall positive association between body-size and population trends across the amniote studies could have a number of drivers that would benefit from further investigation. One putative explanation that has been put forward for positive size trends is that anthropogenic dispersal limitations (generally considered to act more strongly against smaller species) may be having a greater immediate impact than climate change^34^. There are also indications of differences between terrestrial and marine systems. Previous work with the same datasets^1,29^ has found greater species richness and abundance changes in marine than terrestrial systems, whilst here we see a signal of greater trait changes in the (largely terrestrial) amniotes.

In our dataset, the fish length trait studies displayed a particularly skewed distribution of *τ* values (Fig. 2c), with a modal peak of studies showing small negative values then a tail of strongly positive relationships. This guild is also the most likely to have experienced sustained anthropogenic pressure^35^, and many of the ‘fish’ datasets in BioTIME include data from surveys of actively fished and managed areas. Accurately quantifying marine community trends is a challenge^36,37^, but this pattern could reflect the imposition or relaxation of anthropogenic pressure across marine systems^38,39^. Positive *τ* values could represent recoveries from past pressures on larger species, and positive *τ* values were associated with shorter study durations in the fish studies (Extended Data Fig. 4).

Our analysis necessarily sacrifices fine resolution for global scale. Technically, BioTIME studies represent assemblages defined by taxonomy and sampling protocol rather than complete ecological communities. We must implicitly assume that the scope of each study within BioTIME strikes a reasonable balance between the need to include a sufficiently diverse set of species to be able to observe any potential impact of trait differences whilst maintaining meaningful comparability. Limitations to total time series lengths and the limited range of sizes recorded within each dataset inevitably constrain our capacity to detect gradual changes or subtle influences of size. Although the lack of consistent study-level drivers of *τ* suggests that the results are unlikely to be solely determined by the inevitable spatial and temporal limitations of the BioTIME database, future work should seek to improve the scope and resolution of available data to enable more strongly parametric analyses and examine additional measures of community change.

Whilst available trait databases of amniotes and fish are carefully curated, checked and taxonomically tidy, the other databases pose more problems in terms of taxonomic matching and consistency of trait measurements. Without direct correspondence between the sources of dynamics and trait data, it is necessary to take traits as fixed values for each species, despite known differences in traits in time^8,40–42^ and space^43^ that may themselves represent responses to global change. However, in Celtic Sea fish, within-species shifts have been shown to contribute less to community-level size shifts than changes in species composition^44^. We also note that ‘size’ traits for indeterminately growing plants have a less clear meaning than for animals. However, both seed size and maximum height are linked to environmental variables^45,46^, plant size is linked to life history^47,48^ and changes in community height driven by species turnover have been observed in tundra plants^15^.

Many of the criticisms and defences regarding earlier studies using the BioTIME dataset, and indeed other analyses of large collections of time series, also apply to this work^49,50^. The consistency between the alternative approaches we tested to determine population trends (Extended Data Fig. 3) demonstrates that our conclusions are not dependent on particular data transformation choices. However, a largely non-parametric statistical approach was necessitated by the unevenness of the available data, and it must be noted that it could lack the power and resolution to identify subtle changes. Biases in the underlying BioTIME database towards vertebrate taxa, particular biomes and temperate North American and European sites^23^ are further exaggerated when crossed with trait data availability (Fig. 1). One particularly concerning gap is the absence of any insect studies in our dataset due to a paucity of usable trait information. Observations suggest that there have been considerable changes in the structure of insect communities^34,51,52^. Developing comprehensive insect trait datasets, including using proxies and data imputation, will be crucial to address this deficit^53–55^.

In conclusion, despite necessary reservations, this global analysis suggests that examples of relative increases of larger species^11,34^ may in fact be as frequent as shifts towards smaller-sized species^16^. Community responses appear to be considerably more nuanced and localized than previously considered based on theoretical macro-ecological expectations^7^.

## Methods

### Generating assemblage time series

We downloaded all studies available in the ‘open’ component of the BioTIME database of community time series^23^ from 10.5281/zenodo.3265871. BioTIME contains observations from both fixed plots (repeat measures from the same set of specific localized sites) and from wide-ranging surveys and transects that may not necessarily precisely align year on year. We followed previous approaches^1^ and first identified studies as ‘multi-site’ or ‘single-site’ based on the number of coordinates in the BioTIME database. Single-site studies were considered as one combined assemblage, whilst widely dispersed ‘multi-site’ studies were portioned into assemblages based on a global hexagonal grid of 96 km^2^ cells using dggridR^56^. We retained records from assemblages with abundance or biomass data of at least 10 distinct species and at least 5 years between the first and last record.

### Cleaning names

Although the majority of the records are identified with binomials to species level, a portion of the records in the BioTIME database are labelled only at higher taxonomic levels. For simplicity, we refer to all distinct names as ‘species’. We identified uninformative labels (for example ‘spA’, ‘unidentified’, ‘Miscellaneous’, ‘larvae’, ‘grass’), and common names (mostly birds) were converted to binomials using the Encyclopaedia of Life tool via the taxize R package^57,58^ followed by manual inspection based on study location and species distribution where multiple options were presented. We excluded studies where the species are listed using codes. Informative names were standardized against the Global Biodiversity Information Facility name backbone^59^ using ‘taxize’. The dominant kingdom represented in each study was used to distinguish homonyms. Where BioTIME included only a genus-level identification, we matched these to genus-level size trait values listed in trait databases. Where BioTIME only included taxonomic information of higher rank than genus, we did not attempt to match the traits.

### Trait data

We used four separate trait databases that include some measure of organism size, but we did not mix information between databases. For amniotes, the life history database was downloaded from 10.6084/m9.figshare.c.3308127.v1^24^ from which we used the ‘adult_body_mass_g’ field. For plants, we downloaded from the TRY database (https://www.try-db.org/)^26^ all records of ‘seed dry mass’ (trait 26) and ‘plant height vegetative’ (trait 3106). We grouped these by accepted species name, and calculated the mean of the log_10_(seedmass) values and the maximum observed height. We did not assign a value when the standard deviation of log_10_(seedmass) values was greater than 1. The resultant dataset was derived from 91 original datasets (cited in Supplementary Information). For fish, we downloaded a curated database of fish traits from https://store.pangaea.de/Publications/Beukhof-etal_2019/TraitCollectionFishNAtlanticNEPacificContShelf.xlsx^27^, which in turn is largely based on data from the FishBase database^28^. It is focused on the North Atlantic and Pacific continental shelf, but this represents the majority of the relevant BioTIME studies. It includes values for both genus and species level. We used maximum length, and when there were multiple values for a particular species, we took an average. For marine species, we downloaded size data from the WoRMS database^25^. Aphia identifications (IDs) for all the species in our assemblages (excluding plants and fungi) were identified and used to download all attributes associated with these IDs held on WoRMS using the ‘worrms*’* R package^60^. Quantitative ‘body size’ measurements of length were scaled to millimetre units. We discarded values from stages other than adults, and values corresponding to minimums or thicknesses, then took a mean, except where the values differed by over an order of magnitude, which we discarded. Qualitative body sizes listed on WoRMS are divided into four categories (<0.2 mm, 0.2–2 mm, 2–200 mm, >200 mm), that were carried forwards as simple numbers (1–4). Data not from adults were discarded, and where an ID was associated with multiple distinct size categories, it was discarded.

**Extended Data Fig. 7:**
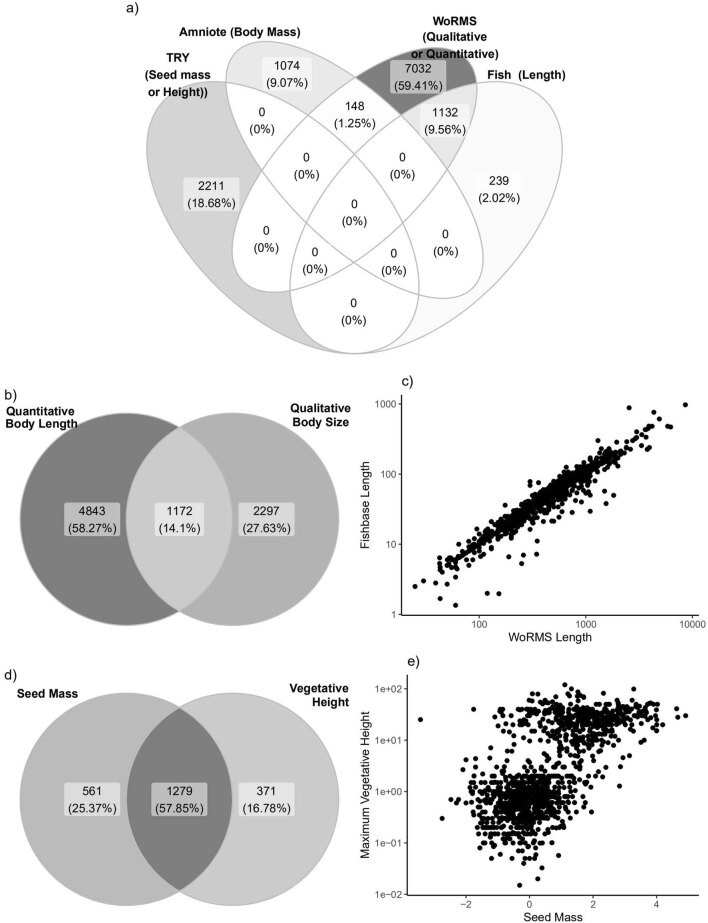
Further details of degree of overlap and correspondence between traits. **a**) Number of species that could be related to at least one trait from the four sources. **b**) Overlap within the WoRMS database between the quantitative and qualitative body lengths was relatively low. In cases where the data was available on both categories, the Spearman’s rank correlation was 0.65. **c**) Very strong correlation between the size traits for species that had data in both the WoRMS and the FishBase databases. **d**) Overlap in trait data between then plant species held in the TRY database was comparatively high. **e**) Correlation between the seed mass and vegetative height trait values was moderate, and considerably less within guilds.

Summaries of the size trait data completeness are given in Extended Data Fig. 7. Note that 66 studies had sufficient data for analysis under multiple size traits: 36 with both categories of plant data, 25 with length data from both WoRMS and the fish-specific database, 1 study spanning the amniote life history traits and WoRMS database, and 4 studies sharing both qualitative and quantitative size information from WoRMS.

### Abundance change–trait correlation

We assessed each assemblage–trait combination where ≥40% and ≥5 of the species had data for that trait and >80% of year samples contained at least 5 species. We excluded transitory species within each assemblage by including only those species that were seen in over half of the year samples. Where this filtering left data from less than 1% of the cells in the original study, we removed the whole study. Where a study included both ‘abundance’ and ‘biomass’ data, we preferentially used the abundance data. Studies with only presence–absence data were not used.

We largely followed a data transformation approach previously established on the BioTIME dataset^3^ for each species time series. Where a species’ time series included repeated trailing or leading zeros, these were cut to one to avoid artificial flattening of the slope. The totals for each species were square-root transformed, then scaled to a mean of 0 and a standard deviation of 1. We fit an ordinary least-squares regression model through the transformed population series against year for each species in the assemblage. The set of slopes (*β*) of these linear models within each dataset summarized the relative change in abundance of each species in the assemblage through time. Very small *β* values (<10^−5^), caused by model fitting errors when there is no change in rank abundance, were set to 0 to avoid spurious rankings. The main response variable *τ* for each assemblage was then computed as Kendall’s rank correlation coefficient between size trait values and the set of *β*s. Species with missing trait values were excluded from the calculation of *τ*. The default *τ*_B_ approach was used for ties^61^. Where there were multiple assemblages per study, study-level *τ* was taken as a simple arithmetic mean of all assemblage-level *τ* values.

We also test two alternative transformations of the population data (Extended Data Figs. 2 and 3): (1) A ranking approach where, within each year, all *n* species in the assemblage were assigned relative ranks (from 1 for the highest to 1/*n* for the lowest) by their abundance or biomass depending on the fields available in BioTIME. Ties were averaged, and where a species was not observed in a particular year, it was assigned a rank of zero for that year. (2) Transformation by dividing each population time series by its mean value.

### Statistics

To generate a null model for the impact of traits, the abundance change slopes (*β*s) were computed as above, but the available trait values (including ‘NA’s where trait data were missing) were randomly reassigned to the species in that assemblage and *τ* was recalculated. This was repeated 10,000 times per assemblage to generate a null distribution of expected *τ* values for each study. The significance of size-trend relationships within each study was determined based on whether the observed *τ* value fell within the central 95% interval of the null distribution. Similarly, the significance of overall patterns within each size trait was determined by comparison of the observed mean *τ* value across all studies within the trait, with the distribution of within-trait means from the randomized dataset.

To examine study-level determinates of *τ* within each size trait, for each study we calculated: (1) the mean total species richness of each assemblage over the time frame, (2) the mean assemblage-level trait data completeness, (3) the mean number of years from which there were data, (4) the mean span of years from which there were data, (5) the log_10_-transformed number of assemblages within the study (that is, the spatial extent), (6) the absolute latitude of the centre of the study and (7) the range of traits in the assemblage (log_10_(max) − log_10_(min)). We fitted a set of linear models to assess whether these factors could predict either *τ* or *τ*^2^.

In a secondary analysis that emphasizes species-level changes, we tested whether relative size within an assemblage affects the likelihood that a species can be clearly identified as increasing or decreasing its population. We focused on those species observed in at least five different years over the time series. Following previous work with the dataset^3^, we assigned each species as either a ‘winner’, ‘loser’ or without an identifiable trend based on the sign and significance (*P* < 0.1) of the year terms (*β*s) described above (Supplementary Table 4). Then, within each trait, we conducted separate logistic regressions to test for significant relationships between the relative trait rank and the likelihood of a species’ being identified as either a ‘winner’ or (in separate tests) a ‘loser’. To prevent domination by species that occur in many assemblages within a study, the regression was downweighted by the number of assemblages in which each species appeared within each study.

### Reporting summary

Further information on research design is available in the Nature Research Reporting Summary linked to this article.

## Supplementary information

Supplementary Information Supplementary Tables 1–4 and data source references.

Reporting Summary

Peer Review Information.

Supplementary Data 1 Core results table, including study IDs and references.

## Data Availability

Original sources of open-source datasets are listed in the Methods. Core results and list of BioTIME studies used are available in .csv format as Supplementary Data 1. Full processed data are available alongside analysis code at https://github.com/jcdterry/BioTIME_BodySize and archived on Zenodo^62^.

## Code availability

All analyses were conducted using R. Code and illustrative notebooks to reproduce all steps are available at https://github.com/jcdterry/BioTIME_BodySize and archived on Zenodo^62^.
