# Supplementary material for: No pervasive relationship between species size and local abundance trends
Source: Nat Ecol Evol. 2021 Dec 30;6(2):140–4. doi: 10.1038/s41559-021-01624-8 (PMC8825279; doi:10.1038/s41559-021-01624-8)
Supplement: Supplementary file 1 — Supplementary Tables 1–4 and data source references. [file 41559_2021_1624_MOESM1_ESM.pdf]

nature portfolio

https://doi.org/10.1038/s41559-021-01624-8

## Supplementary information

# No pervasive relationship between species size and local abundance trends

In the format provided by the authors and unedited

# Supplementary Information accompanying 'No pervasive relationship between species size and local abundance trends'

JCD Terry, J D O'Sullivan and AG Rossberg

Contents: Supplementary Tables 1-4 and original data citations from within the BioTIME and TRY databases.

| | Marine | | Fish | | Amniotes | | Plants | | | |
|---|---|---|---|---|---|---|---|---|---|---|
| | Body Length | | Maximum Length | | Adult body mass | | Seed Mass | | Maximum Height | |
| | Estimate | p-value | Estimate | p-value | Estimate | p-value | Estimate | p-value | Estimate | p-value |
| **Coefficents** | | | | | | | | | | |
| Intercept | 0.0142 | 0.924 | -0.027 | 0.878 | 0.688 | **0.0111** | 0.187 | 0.348 | 0.0429 | 0.917 |
| Species Richness | -0.0203 | 0.321 | -0.106 | **0.00159** | -0.056 | 0.261 | 0.0607 | 0.644 | 0.272 | 0.31 |
| Number of Cells (Log10) | -0.000569 | 0.0988 | 0.000806 | 0.357 | 0.000811 | 0.672 | -0.000225 | 0.628 | -0.000394 | 0.631 |
| Years Sampled | -0.00164 | 0.634 | -0.0106 | **0.039** | -0.00513 | 0.578 | 0.00981 | **0.0139** | 0.0136 | 0.0593 |
| Year Range | -0.00243 | 0.427 | 0.00447 | 0.326 | 0.00513 | 0.565 | -0.00771 | **0.013** | -0.0111 | **0.0345** |
| Trait Range | 0.125 | **0.0373** | -0.0956 | 0.385 | -0.111 | **0.0108** | -0.0203 | 0.515 | 0.0531 | 0.577 |
| Absolute Latitude | -0.00133 | 0.419 | 0.0069 | **0.00637** | 0.00108 | 0.69 | -0.00349 | 0.216 | -0.000315 | 0.943 |
| **Summary** | | | | | | | | | | |
| Trait Completeness | 0.0198 | 0.868 | -0.0139 | 0.918 | -0.561 | **0.035** | 0.212 | 0.296 | 0.0996 | 0.765 |
| Observations | 52 | | 26 | | 42 | | 43 | | 40 | |
| Adjusted R-squared | 0.122 | | 0.552 | | 0.0718 | | 0.101 | | 0.105 | |

**Extended Data Table 1** Linear model analysis of putative study-level predictors of size trait − population trend correlations ($\tau$). We tested each trait separately, except 'Qualitative body size', for which there were too few studies for a meaningful test. No corrections were made to the reported values for multiple comparisons. There were no consistent drivers - while certain relationships between possible predictor variables were identified as significant at $p<0.05$ (highlighted in **bold**), these were not consistent across the set of traits and had low explanatory power (adjusted-$R^2$) in all cases except Fish (as discussed in the main text).

| | Marine | | Fish | | Amniotes | | Plants | | | |
|---|---|---|---|---|---|---|---|---|---|---|
| | Body Length | | Maximum Length | | Adult body mass | | Seed Mass | | Maximum Height | |
| | Estimate | p-value | Estimate | p-value | Estimate | p-value | Estimate | p-value | Estimate | p-value |
| **Coefficents** | | | | | | | | | | |
| Intercept | -0.00667 | 0.779 | -0.0831 | 0.12 | 0.139 | 0.125 | 0.0693 | 0.265 | 0.193 | 0.265 |
| Species Richness | -0.0132 | **0.000181** | -0.0307 | **0.00178** | -0.0164 | 0.336 | -0.0302 | 0.461 | -0.11 | 0.32 |
| Number of Cells (Log10) | -5.05e-05 | 0.355 | 9.75e-05 | 0.701 | -0.00043 | 0.515 | -0.00023 | 0.118 | -0.000342 | 0.317 |
| Years Sampled | -0.000555 | 0.317 | -0.00194 | 0.183 | -0.000276 | 0.931 | 0.000585 | 0.624 | -0.00194 | 0.507 |
| Year Range | 0.00071 | 0.151 | 0.00197 | 0.147 | 0.000898 | 0.77 | -0.000585 | 0.528 | 0.0016 | 0.447 |
| Trait Range | 0.000782 | 0.934 | 0.0117 | 0.715 | -0.00485 | 0.735 | 0.00265 | 0.784 | -0.00754 | 0.848 |
| Absolute Latitude | 0.00048 | 0.0729 | 0.00219 | **0.00359** | 0.000556 | 0.55 | -6.24e-05 | 0.943 | -0.000647 | 0.722 |
| **Summary** | | | | | | | | | | |
| Trait Completeness | 0.0226 | 0.24 | 0.0484 | 0.23 | -0.123 | 0.172 | -0.0245 | 0.696 | -0.0905 | 0.514 |
| Observations | 52 | | 26 | | 42 | | 43 | | 40 | |
| Adjusted R-squared | 0.271 | | 0.51 | | -0.0407 | | -0.0545 | | -0.0555 | |

**Extended Data Table 2** Full statistical results of tests for the drivers of $\tau^2$, in order to test if there are drivers for deviations from trait-neutrality. Each trait was tested independently and no corrections were made to the reported values for multiple comparisons. Here too there were no consistent drivers.

| Trait | Likelihood of being ‘Winner’ | | |
|---|---|---|---|
| | Coefficient estimate | SE | p-value |
| Marine: Body Length | -0.066 | -0.095 | 0.277 |
| Marine: Qualitative Body Size | -1.269 | -0.831 | 1.151 |
| Fish: Maximum Length | -0.170 | 0.088 | 0.439 |
| Amniotes: Adult body mass | 0.490 | -0.248 | 0.262 |
| Plants: Seed Mass | 0.331 | 0.058 | 0.273 |
| Plants: Maximum Height | -0.084 | -0.158 | 0.318 |

| Trait | Likelihood of being ‘Loser’ | | |
|---|---|---|---|
| | Coefficient estimate | SE | p-value |
| Marine: Body Length | 0.263 | 0.811 | 0.719 |
| Marine: Qualitative Body Size | 1.548 | 0.270 | 0.591 |
| Fish: Maximum Length | 0.479 | 0.699 | 0.854 |
| Amniotes: Adult body mass | 0.283 | 0.061 | 0.382 |
| Plants: Seed Mass | 0.275 | 0.225 | 0.832 |
| Plants: Maximum Height | 0.334 | 0.791 | 0.635 |

**Extended Data Table 3** Results from logistic regressions seeking to determine if the relative trait rank within an assemblage predicts the likelihood of an identifiable population level trend. Separate analyses were carried out for ‘winner’ and ‘loser’ categories. Each test was a logistic regression, weighted by the reciprocal of the number of cells within a study that include the species. Coefficient estimates are shown on the transformed scale. Separate models were fit for each trait and direction of trend, and no corrections were made for multiple comparisons.

| Trait | Loser | No Clear Trend | Winner |
|---|---|---|---|
| Amniotes: Adult body mass | 229.9 | 718.0 | 280.1 |
| Marine: Body Length | 259.4 | 2130.7 | 237.9 |
| Plants: Maximum Height | 195.5 | 333.0 | 278.5 |
| Fish: Maximum Length | 96.8 | 871.0 | 114.1 |
| Plants: Seed Mass | 356.9 | 583.6 | 333.5 |
| Marine: Qualitative Body Size | 29.9 | 256.0 | 45.1 |

**Extended Data Table 4** Total counts of the number of species in each category for each trait. Species that are found in multiple studies are counted each time. Where species have different trends in different cells within a study they are assigned fractionally. For example, a species that was observed in 10 assemblages within a study, and was observed to be a 'winner' in two and 'no clear trend' in eight is counted as 0.2 of a winner and 0.8 of no trend.

# Original BioTIME Citations

Citations for the original studies used from the BioTIME database. See Supplementary Data 1 for which citation is linked to which study ID.


1. Webb, S. L. & Scanga, S. E. (2001) Windstorm Disturbance without Patch Dynamics: Twelve Years of Change in a Minnesota Forest. Ecology, 82, 893 –897.

2. Zachmann, L., Moffet, C. & Adler, P. (2010) Mapped quadrats in sagebrush steppe: long-term data for analyzing demographic rates and plant–plant interactions. Ecology, 91, 3427.

3. Holmes, R. T. & Sherry, T. W. (2001) Thirty-year bird population trends in an unfragmented temperate deciduous forest: the importance of habitat change. The Auk, 118, 589–610.

4. Holmes, R. T. & Sturges, F. W. (1975) Bird community dynamics and energetics in a northern hardwoods ecosystem. Journal of Animal Ecology, 44, 175–200.

5. Holmes, R. T. & Sherry, T. W. (1988) Assessing population trends of New Hampshire forest birds: Local versus regional patterns. The Auk, 105, 756–768.

6. Holmes, R. T., Sherry, T. W. & Sturges, F. W. (1986) Bird Community Dynamics in a Temperate Deciduous Forest: Long-Term Trends at Hubbard Brook. Ecological Monographs, 56, 201–220.

7. Preston, F. W. (1960) Time and Space and the Variation of Species. Ecology, 41, 611–627.

8. Brooks, A.J. "MCR LTER: Coral Reef: Long-term Population and Community Dynamics: Fishes". Moorea Coral Reef. Available at: http://mcr.lternet.edu/cgi-bin/showDataset.cgi?docid=knb-lter-mcr.6, accessed 2012.

9. Brooks, A.J. (2016) Moorea Coral Reef LTER: Coral Reef: Long-term Population and Community Dynamics: Fishes. Available at: knb-lter-mcr.6.54 doi:10.6073/pasta/d688610e536f54885a3c59d287f6c4c3, accessed 2016.

10. Williamson, M. (1983) The Land-Bird Community of Skokholm: Ordination and Turnover. Oikos, 41, 378–384.

11. Vickery, W. L. & Nudds, T. D. (1984) Detection of Density-Dependent Effects in Annual Duck Censuses. Ecology, 65, 96.

12. Friggens, M. (2008) "Sevilleta LTER Small Mammal Population Data", Albuquerque, NM: Sevilleta Long Term Ecological Research Site Database: SEV008. Available at: http://sev.lternet.edu/data/sev-8, accessed 2012.

13. Waide, R. B. (2010) "Bird abundance - point counts". Long Term Ecological Research Network. Available at: http://dx.doi.org/10.6073/pasta/0d96957379936a038ebbbcc6135b2fab, accessed 2012.

14. Waide, R. B. "Bird abundance - point counts, El Verde Field Station, Puerto Rico: Luquillo Long Term Ecological Research Site Database: Data Set 23". Available at: http:/luq.lternet.edu/data/luqmetadata23, accessed 2012.

15. Ernest, S., Valone, T.J. & Brown, J.H. (2009) Long?term monitoring and experimental manipulation of a Chihuahuan Desert ecosystem near Portal, Arizona, USA. Ecology, 90, 1708-1708.

16. Condit, R., Lao, S., Pérez, R., Dolins, S.B., Foster, R. & Hubbell, S. (2012) Dataset: Barro Colorado Forest Census Plot Data (Version 2012).

17. Condit, R. (1998) Tropical forest census plots: Methods and results from Barro Colorado Island, Panama and a Comparison with other plot. Springer Verlag and RG Landes Company, Berlin,

18. Condit, R., Ashton, P., Bunyavejchewin, S., Dattaraja, H., Davies, S., Esufali, S., Ewango, C., Foster, R., Gunatilleke, I. & Gunatilleke, C. (2006) The importance of demographic niches to tree diversity. Science, 313, 98-101.

19. Condit, R., Chisholm, R.A. & Hubbell, S.P. (2012) Thirty years of forest census at Barro Colorado and the importance of immigration in maintaining diversity. PloS one, 7, e49826.

20. Hubbell, S. P., Condit, R. & Foster, R. B. (2005) "Barro Colorado Forest Census Plot Data". Available at: https://ctfs.arnarb.harvard.edu/webatlas/datasets/bci, accessed 2012.

21. Condit, R., Pérez, R., Aguilar, S., Lao, S., Robin, F. & Hubbell, S. (2018) Tree species abundance through time in tropical forest census plots, Panama. DataONE Dash, Dataset, , Available at: https://doi.org/10.15146/R3MM4V

22. "Animal Demography Unit - Coordinated Waterbird Counts (CWAC) - AfrOBIS". Available at http://www.iobis.org/, accessed 2012.

23. Zettler, M.L. (2005) Macrozoobenthos baltic sea (1980-2005) as part of the IOW-Monitoring. Institut für Ostseeforschung Warnemünde, Germany. Available at: http://www.iobis.org/mapper/?dataset=2289, accessed 2012.

24. "Baltic Seabirds Transect Surveys", Institute of Ecology of Vilnius University - OBIS-SEAMAP. Available at: http://www.emodnet-biology.eu/component/imis/?module=dataset&dasid=1971, accessed 2012.

25. Markhaseva, E.L., Golikov, A.A., Agapova, T.A. & Beig, A.A. (1985) Archives of the Arctic Seas Zooplankton. Available at: http://www.iobis.org/mapper/?dataset=4470, accessed 2012.

26. "CSIRO Marine Data Warehouse - OBIS Australia", CSIRO Division of Marine and Atmospheric Research (CMAR), Australia. Available at http://www.iobis.org, accessed 2012.

27. "South Western Pacific Regional OBIS Data Asteroid Subset", NIWA (National Institute of Water and Atmospheric Research - New Zealand) MBIS (Marine Biodata Information System) accessed through South Western Pacific OBIS. Available at: http://www.iobis.org/mapper/?dataset=219, accessed 2012.

28. Clark, D. & Branton, B. (2007) DFO Maritimes Research Vessel Trawl Surveys, OBIS Canada Digital Collections. Bedford Institute of Oceanography, Dartmouth, Nova Scotia, Canada, OBIS Canada,

29. "CRED Towed-Diver Fish Biomass Surveys in the Pacific Ocean 2000-2010". (2011) Coral Reef Ecosystem Division (CRED), Pacific Island Fisheries Sciences Center, National Marine Fisheries Service. Available at: http://www.iobis.org/mapper/?dataset=1581, accessed 2012.

30. Sherman, S. (2010) "Maine Department of Marine Resources Inshore Trawl Survey, 2000 – 2009". Maine Department of Marine Resources, Maine. Available at: http://www.usgs.gov/obis-usa/data_search_and_access/datasets.html, accessed 2012.

31. Reichert, M. (2009) "MARMAP Chevron Trap Survey 1990-2009". SCDNR/NOAA MARMAP Program, SCDNR MARMAP Aggregate Data Surveys, The Marine Resources Monitoring, Assessment, and Prediction (MARMAP) Program, Marine Resources Research Institute, South Carolina Department of Natural Resources U.S.A.. Available at: http://www.usgs.gov/obis-usa/data_search_and_access/participants.html, accessed 2012.

32. Reichert, M. (2010) "MARMAP Neuston Nets 1990-2009". SCDNR/NOAA MARMAP Program, SCDNR MARMAP Aggregate data surveys, The Marine Resources Monitoring, Assessment, and Prediction (MARMAP) Program, Marine Resources Research Institute, South Carolina Department of Natural Resources U.S.A.. Available at: http://www.usgs.gov/obis-usa/data_search_and_access/participants.html, accessed 2012.

33. Reichert, M. (2009) “MARMAP Florida Antillean Trap Survey 1990-2009”. SCDNR/NOAA MARMAP Program, SCDNR MARMAP Aggregate Data Surveys, The Marine Resources Monitoring, Assessment, and Prediction (MARMAP) Program, Marine Resources Research Institute, South Carolina Department of Natural Resources U.S.A.. Available at: http://www.usgs.gov/obis-usa/data_search_and_access/participants.html, accessed 2012.

34. Björnberg, T. (1963) On the marine free-living copepods off Brazil. Boletim do Instituto oceanogràfico, 13, 03-142.

35. Silveira, F. L. & Lopes, R. M. (2008) “On the Marine Free-Living Copepods off Brazil -WSAOBIS”. Western South Atlantic OBIS, São Paulo. Available at: http://www.iobis.org, accessed 2012.

36. Escribano, R., Manríquez, K. & Godoy, F. (2006) “Copepoda-COPAS Center (COPAS_CPD1) - Planktonic copepods from the Chilean Humboldt Current System - Eastern South Pacific Regional Node of OBIS (ESPOBIS)”. Available at: http://www.iobis.org, accessed 2012.

37. Hidalgo, P., Escribano, R., Vergara, O., Jorquera, E., Donoso, K. & Mendoza, P. (2010) Patterns of copepod diversity in the Chilean coastal upwelling system. Deep Sea Research Part II: Topical Studies in Oceanography, 57, 2089–2097.

38. “South Western Pacific Regional OBIS Data provider for the NIWA Marine Biodata Information System”. Ocean Biogeographic Information System. Occurrence Dataset. Available at: https://doi.org/10.15468/zuuiyu, accessed 2012.

39. “The Observer Program database”, accessed through the OBIS-USA North Pacific Groundfish Observer (North Pacific Research Board). Available at: http://www.iobis.org, accessed 2012.

40. “PIROP Northwest Atlantic 1965-1992 - OBIS SEAMAP”. Available at: http://www.iobis.org/mapper/?dataset=2245, accessed 2012.

41. Brown, R.G., Nettleship, D.N., Germain, P., Tull, C.E. & Davis, T. (1975) Atlas of eastern Canadian seabirds.

42. Diamond, A., Gaston, A. & Brown, R. (1986) Converting PIROP Counts of Seabirds at Sea to Absolute Densities. Progress Notes No 164. Canadian Wildlife Service, Ottawa,

43. Halpin, P.N., Read, A.J., Fujioka, E., Best, B.D., Donnelly, B., Hazen, L.J., Kot, C., Urian, K., LaBrecque, E. & Dimatteo, A. (2009) OBIS-SEAMAP: The world data center for marine mammal, sea bird, and sea turtle distributions. Oceanography, 22, 104-115.

44. Huettmann, F. (1998) An ecological GIS research application for the northern Atlantic-The PIROP database software, environmental data sets and the role of the internet. In: Riekert W.-F. and Tochtermann K. (Eds.) Hypermedia im Umweltschutz Proceedings of Deutsche Gesellschaft für Informatik (GI) and Forschungsinstitut für anwendungsorientierte Wissensverarbeitung (FAW) Ulm. Umwelt-Informatik aktuell; Bd.17, Metropolis Verlag/Marburg. pp. 213-217

45. Read, A., Halpin, P., Crowder, L., Best, B. & Fujioka, E. (2010) OBIS-SEAMAP: mapping marine mammals, birds and turtles. World Wide Web electronic publication. http://seamap. env. duke. Edu

46. Kennedy, M. & Spry, J. (2011) Atlantic Zone Monitoring Program Maritimes Region plankton datasets. Fisheries and Oceans Canada-BioChem archive. OBIS Canada, Bedford Institute of Oceanography, Dartmouth, Nova Scotia, Canada.

47. “East Coast North America Strategic Assessment Project, Groundfish Atlas for the East Coast of North America”. Available at: http://www.iobis.org, accessed 2012.

48. Wade, E. (2011) Snow crab research trawl survey database (Southern Gulf of St. Lawrence, Gulf region, Canada) from 1988 to 2010. OBIS Canada, Bedford Institute of Oceanography, Dartmouth, Nova Scotia, Canada,

49. Tremblay, J.M. & Branton, B. (2007) DFO Maritimes Research Vessel Trawl Surveys, OBIS Canada Digital Collections. Bedford Institute of Oceanography, Dartmouth, Nova Scotia, Canada, OBIS Canada.

50. “St. John, USVI Fish Assessment and Monitoring Data (2002 - Present)”, (2007) Silver Spring, MD Publisher: NOAAs Ocean Service, National Centers for Coastal Ocean Science (NCCOS). National Oceanic and Atmospheric Association (NOAA)-National Ocean Service (NOS)-National Centers for Coastal Ocean Science (NCCOS)-Center for Coastal Monitoring and Assessment (CCMA)-Biogeography Team. Available at: http://www.iobis.org/mapper/?dataset=1672, accessed 2012.

51. “St. Croix, USVI Fish Assessment and Monitoring Data (2002 - Present)”, (2007) Silver Spring, MD Publisher: NOAAs Ocean Service, National Centers for Coastal Ocean Science (NCCOS). National Oceanic and Atmospheric Association (NOAA)-National Ocean Service (NOS)-National Centers for Coastal Ocean Science (NCCOS)-Center for Coastal Monitoring and Assessment (CCMA)-Biogeography Team. Available at: http://www.iobis.org/mapper/?dataset=1673, accessed 2012.

52. USGS Patuxent Wildlife Research Center “North American Breeding Bird Survey” ftp data set, version 2014.0. Available at: ftp://ftpext.usgs.gov/pub/er/md/laurel/BBS/DataFiles/, accessed 2013.

53. Sukumar, R. Mudumalai Forest Dynamics Plot Data. Available at: http://www.ctfs.si.edu/site/Mudumalai/, accessed 2013.

54. Degraer, S., Wittoeck, J., Appeltans, W., Cooreman, K., Deprez, T., Hillewaert, H., Hostens, K., Mees, J., Vanden Berghe, E. & Vincx, M. (2006) “Macrobel: Long term trends in the macrobenthos of the Belgian Continental Shelf.” Oostende, Belgium. Available at: http://www.emodnet-biology.eu/data-catalog?module=dataset&dasid=145, accessed 2013.

55. Reichert, M. (2010) “MARMAP Fly Net 1990-2009”. SCDNR/NOAA MARMAP Program, SCDNR MARMAP Aggregate Data Surveys, The Marine Resources Monitoring, Assessment, and Prediction (MARMAP) Program, Marine Resources Research Institute, South Carolina Department of Natural Resources USA. Available at: http://www.usgs.gov/obis-usa/, accessed 2013.

56. Reichert, M. (2010) “MARMAP Yankee Trawl 1990-2009”. SCDNR/NOAA MARMAP Program, SCDNR MARMAP Aggregate data surveys, The Marine Resources Monitoring, Assessment, and Prediction (MARMAP) Program, Marine Resources Research Institute, South Carolina Department of Natural Resources USA. Available at: http://www.usgs.gov/obis-usa, accessed 2013.

57. “Northeast Fisheries Science Center Bottom Trawl Survey Data (OBIS-USA).” (2005) NOAA’s National Marine Fisheries Service (NMFS) Northeast Fisheries Science Center. Woods Hole, Massachusetts, USA. Available at: http://www.iobis.org/mapper/?dataset=1435, accessed 2013.

58. Harmon, M. & Franklin, J. (2012) “Long-term growth, mortality and regeneration of trees in permanent vegetation plots in the Pacific Northwest, 1910 to present.” Long-Term Ecological Research. Forest Science Data Bank, Corvallis. Available at: http://andrewsforest.oregonstate.edu/data/abstract.cfm?dbcode=TV010, accessed 2012.

59. USFS “Landbird Monitoring Program (UMT-LBMP).” US Forest Service. Available at: http://www.avianknowledge.net/, accessed 2012.

60. Cavender-Bares, J. & Reich, P.B. (2012) Shocks to the system: community assembly of the oak savanna in a 40?year fire frequency experiment. Ecology, 93

61. Reich, P., Wedin, D., Hobbie, S. & Davis, M. “Experiment 133 - Effect of Burning Patterns on Vegetation in the Fish Lake Burn Compartments - Shrub Survey”. Cedar Creek Ecosystem Science Reserve. Available at: http://www.cedarcreek.umn.edu/research/data/experiment?e133, accessed 2012.

62. Battles, J.J., Fahey, T. & Cleavitt, N. “Forest Inventory of a Northern Hardwood Forest: Watershed 6 1965, Hubbard Brook Experimental Forest.” The Hubbard Brook Ecosystem Study LTER Program. Available at: http://www.hubbardbrook.org/data/dataset.php?id=29, accessed 2016.

63. Battles, J.J., Fahey, T. & Cleavitt, N. "Forest Inventory of a Northern Hardwood Forest: Watershed 6 1977, Hubbard Brook Experimental Forest." The Hubbard Brook Ecosystem Study LTER Program. Available at: http://www.hubbardbrook.org/data/dataset.php?id=30, accessed 2016.

64. Battles, J.J., Fahey, T. & Cleavitt, N. "Forest Inventory of a Northern Hardwood Forest: Watershed 6 1987, Hubbard Brook Experimental Forest." The Hubbard Brook Ecosystem Study LTER Program. Available at: http://www.hubbardbrook.org/data/dataset.php?id=32, accessed 2016.

65. Battles, J.J., Fahey, T. & Cleavitt, N. "Forest Inventory of a Northern Hardwood Forest: Watershed 6 1992, Hubbard Brook Experimental Forest." The Hubbard Brook Ecosystem Study LTER Program. Available at: http://www.hubbardbrook.org/data/dataset.php?id=33, accessed 2016.

66. Battles, J.J., Fahey, T. & Cleavitt, N. "Forest Inventory of a Northern Hardwood Forest: Watershed 6 1997, Hubbard Brook Experimental Forest." The Hubbard Brook Ecosystem Study LTER Program. Available at: http://www.hubbardbrook.org/data/dataset.php?id=34, accessed 2016.

67. Battles, J.J., Fahey, T. & Cleavitt, N. "Forest Inventory of a Northern Hardwood Forest: Watershed 6 1982, Hubbard Brook Experimental Forest." The Hubbard Brook Ecosystem Study LTER Program. Available at: http://www.hubbardbrook.org/data/dataset.php?id=31, accessed 2016.

68. Battles, J.J., Johnson, C., Hamburg, S., Fahey, T., Driscoll, C. & Likens, G. (2003) "Forest Inventory of a Northern Hardwood Forest: Watershed 6 2002." The Hubbard Brook Ecosystem Study LTER Program. Available at: http://www.hubbardbrook.org/data/dataset.php?id=35, accessed 2012.

69. Battles, J.J., Fahey, T. & Cleavitt, N. (2003) "Forest Inventory of a Whole Tree Harvest: Hubbard Brook Experimental Forest Watershed 5, 1982, pre-harvest." The Hubbard Brook Ecosystem Study LTER Program. Available at: http://www.hubbardbrook.org/data/dataset.php?id=36, accessed 2012.

70. Battles, J.J., Fahey, T. & Cleavitt, N. (2013) "Forest Inventory of a Whole Tree Harvest: Hubbard Brook Experimental Forest Watershed 5, 1990, 7 years post-harvest." The Hubbard Brook Ecosystem Study LTER Program. Available at: http://www.hubbardbrook.org/data/dataset.php?id=37, accessed 2016.

71. Battles, J.J., Fahey, T. & Cleavitt, N. (2013b) "Forest Inventory of a Whole Tree Harvest: Hubbard Brook Experimental Forest Watershed 5, 1994, 10 years post-harvest." The Hubbard Brook Ecosystem Study LTER Program. Available at: http://www.hubbardbrook.org/data/dataset.php?id=38, accessed 2016.

72. Battles, J.J., Fahey, T. & Cleavitt, N. (2013c) "Forest Inventory of a Whole Tree Harvest: Hubbard Brook Experimental Forest Watershed 5, 1999, 15 years post-harvest." The Hubbard Brook Ecosystem Study LTER Program. Available at: http://www.hubbardbrook.org/data/dataset.php?id=39, accessed 2016.

73. Muldavin, E. "Pinon-Juniper (Core Site) Quadrat Data for the Net Primary Production Study at the Sevilleta National Wildlife Refuge, New Mexico (2003-Present)." Sevilleta Long Term Ecological Research Program. Available at: http://sev.lternet.edu/node/1718, accessed 2013.

74. Condit, R. "Sherman Forest Dynamics Plot, Panama." The Center for Tropical Forest Science. Smithsonian Tropical Research Institute. Available at: http://www.ctfs.si.edu/site/Sherman/, accessed 2013.

75. Paquette, A., Laliberté, E., Bouchard, A., Blois, S. de, Legendre, P. & Brisson, J. (2007) Lac Croche understory vegetation data set (1998–2006). Ecology, 88, 3209. doi:10.1890/07-0513.1

76. Day, F. (2010) "Long-term N-fertilized vegetation plots on Hog Island, Virginia Coastal Barrier Islands, 1992-2014." Virginia Coast Reserve Long-Term Ecological Research Project. Available at: http://www.vcrlter.virginia.edu/cgi-bin/showDataset.cgi?docid=knb-lter-vcr.106, accessed 2013.

77. Day, F. P., Conn, C., Crawford, E. & Stevenson, M. (2004) Long-term effects of nitrogen fertilization on plant community structure on a coastal barrier island dune chronosequence. Journal of Coastal Research, 20, 722–730.

78. Chen, H., Liao, Y.-C., Chen, C.-Y., Tsai, J.-I., Chen, L.-S. & Shao, K.-T. (2015) Long-term monitoring dataset of fish assemblages impinged at nuclear power plants in northern Taiwan. Scientific data, 2, 150071.

79. Shi, Z., Sherry, R., Xu, X., Hararuk, O., Souza, L., Jiang, L., Xia, J., Liang, J. & Luo, Y. (2015) Evidence for long-term shift in plant community composition under decadal experimental warming. Journal of Ecology, 103, 1131-1140.

80. Reichert, M. (2010) "MARMAP Blackfish Trap Survey 1990-2009". SCDNR/NOAA MARMAP Program. SCDNR MARMAP Aggregate Data Surveys. The Marine Resources Monitoring. Assessment. and Prediction (MARMAP) Program. Marine Resources Research Institute. South Carolina Department of Natural Resources USA. Available at: http://www.usgs.gov/obis-usa/. accessed 2013.

81. Woods, K. D. (2009) Multi-decade, spatially explicit population studies of canopy dynamics in Michigan old-growth forests. Ecology, 90, 3587.

82. Reed, D. C. (2014a) "SBC LTER: Reef: Kelp forest community dynamics: Abundance and size of giant kelp (Macrocystis pyrifera), ongoing since 2000". Santa Barbara Coastal LTER. Available at: http://sbc.lternet.edu/cgi-bin/showDataset.cgi?docid=knb-lter-sbc.18, accessed 2016. doi:10.6073/pasta/d90872297e30026b263a119d4f5bca9f

83. Davis, R. A. & Doherty, T. S. (2015) Rapid Recovery of an Urban Remnant Reptile Community following Summer Wildfire. PLoS ONE 10(5), e0127925. doi: 10.1371/journal.pone.0127925.

84. Edgar, G. J. & Stuart-Smith, R. D. (2014) Systematic global assessment of reef fish communities by the Reef Life Survey program. Nature Scientific Data 1, 140007. doi:10.1038/sdata.2014.7.

85. Adler, P.B., Tyburczy, W.R. & Lauenroth, W.K. (2007) Long-term mapped quadrats from Kansas prairie: demographic information for herbaceaous plants. Ecology, 88, 2673-2673.

86. "The Main Cropping System Experiment (MCSE)". KBS LTER, Kellogg Biological Station. Available at: http://lter.kbs.msu.edu/research/long-term-experiments/main-cropping-system-experiment/, accessed 2016.

87. Merritt, J. (1999) Long Term Mammal Data from Powdermill Biological Station 1979-1999. Environmental Data Initiative. Available at: http://dx.doi.org/10.6073/pasta/83c888854e239a79597999895bb61cfe, accessed 2016.

88. Kaufman, D.W. Seasonal summary of numbers of small mammals on 14 LTER traplines in prairie habitats at Konza Prairie. Konza Prairie Long-Term Ecological Research. Available at: http://lter.konza.ksu.edu/content/csm01-seasonal-summary-numbers-small-mammals-14-lter-traplines-prairie-habitats-konza, accessed 2016.

89. Prins, H. H. T. & Douglas-Hamilton, I. (1990) Stability in a Multi-Species Assemblage of Large Herbivores in East Africa. Oecologia, 83, 392–400.

90. Lightfoot, D. (2013) "Lizard pitfall trap data (LTER-II, LTER-III)". Jornada Basin LTER. Available at: http://jornada.nmsu.edu/lter/dataset/49821/view, accessed 2016.

91. Wilgers, D. J., Horne, E. A., Sandercock, B. K. & Volkmann, A. W. (2006) Effects of rangeland management on community dynamics of the herpetofauna of the tallgrass prairie. Herpetologica, 62, 378–388.

92. Lightfoot, D. & Schooley, R. L. "SMES rodent trapping data, Small Mammal Exclosure Study". Jornada LTER. Available at: http://jornada.nmsu.edu/sites/jornada.nmsu.edu/files/data_files/JornadaStudy_086_smes_rodent_trapping_data_0.csv, accessed 2016.

93. Kelt, D. A., Meserve, P. L., Gutiérrez, J. R., Milstead, W. B. & Previtali, M. A. (2013) Long-term monitoring of mammals in the face of biotic and abiotic influences at a semiarid site in north-central Chile. Ecology, 94, 977. doi:10.1890/12-1811.1.

94. Davies, C. H., Armstrong, A. J., Baird, M., Coman, F., Edgar, S., Gaughan, D., Greenwood, J., Gusmão, F., Henschke, N., Koslow, J. A., Leterme, S. C., McKinnon, A. D., Miller, M., Pausina, S., Palomino, J. U., Roennfeldt, R.-L., Rothlisberg, P., Slotwinski, A., Strzelecki, J., Suthers, I. M., Swadling, K. M., Talbot, S., Tonks, M., Tranter, D. H., Young, J. W. & Richardson, A. J. (2014) Over 75 years of zooplankton data from Australia. Ecology, 95, 3229. http://dx.doi.org/10.1890/14-0697.1.

95. Mack, M. C., Schuur, E. A. G., Bret-Harte, M. S., Shaver, G. R. & Chapin, F. S. (2004) Ecosystem carbon storage in arctic tundra reduced by long-term nutrient fertilization. Nature, 431, 440–443.

96. Shaver, G. (2015) "Above ground plant biomass a moist acidic tussock tundra experimental site, 1984, Artic LTER, Toolik Lake, Alaska". Available at: http://dx.doi.org/10.6073/pasta/08a91cb2697f7cdc82d654e82b53c5c5, accessed 2016.

97. Shaver, G. R. & Chapin, F. S. (1991) Production: biomass relationships and element cycling in contrasting arctic vegetation types. Ecological Monographs, 61(1), 1–31.

98. Svensson, S., Thorner, A. M. & Nyholm, N. E. I. (2010) Species trends, turnover and composition of a woodland bird community in southern Sweden during a period of 57 years. Ornis Svecica 20, 31–44.

99. Lightfoot, D. (2011) "Small Mammal Exclosure Study (SMES) Vegetation Data from the Chihuahuan Desert Grassland and Shrubland at the Sevilleta National Wildlife Refuge, New Mexico (2006-2009)". Long Term Ecological Research Network. Available at: http://dx.doi.org/10.6073/pasta/d80d5e2196cd11ef79df23ebe5a77c19, accessed 2016.

100. Laguionie-Marchais, C., Billett, D. S. M., Paterson, G. L. D., Ruhl, H. A., Soto, E. H., Smith, J. L. & Thatje, S. (2013) Inter-annual dynamics of abyssal polychaete communities in the North East Pacific and North East Atlantic-A family-level study. Deep-Sea Research Part I: Oceanographic Research Papers, 75, 175–186.

101. Laguionie-Marchais, C., Paterson, G. L. J., Bett, B. J., Smith, K. L. & Ruhl, H. A. (2016) Inter-annual species-level variations in an abyssal polychaete assemblage (Sta. M, NE Pacific, 4000 m). Progress in Oceanography, 140, 43–53.

102. Hartnett, D.C. & Collins, S.L. (2016) PVC02 Plant Species Composition on Selected Watersheds at Konza Prairie. Environmental Data Initiative. Available at: http://dx.doi.org/10.6073/pasta/7b6df00de4d0fcecfd344c02de9f9c62, accessed 2017.

103. Bradford, M.G., Murphy, H.T., Ford, A.J., Hogan, D.L. & Metcalfe, D.J. (2014) Long?term stem inventory data from tropical rain forest plots in Australia. Ecology, 95, 2362-2362.

104. Dickson, J.G., Conner, R.N. & Williamson, J.H. (1993) Neotropical migratory bird communities in a developing pine plantation. Procedings on the Annual Conference. SEAFWA, 47, 439-446.

105. Reed, D. C. (2014) "SBC LTER: Reef: Kelp Forest Community Dynamics: Fish abundance". Santa Barbara Coastal LTER. Available at: doi:10.6073/pasta/e37ed29111b2fddffc08355252b8b8c7, accessed 2016.

106. Tomialojc, L. & Wesolowski, T. (1994) Die Stabilität der Vogelgemeinschaft in einem Urwald der gemässigten Zone: Ergebnisse einer 15jährigen Studie aus dem Nationalpark von Bialowieza (Polen). Beob, 91, 73-110.

107. Tomialojc, L. & Wesolowski, T. (1996) Structure of a primaeval forest bird community during 1970s and 1990s (Bialowieza National Park, Poland). Acta Ornithologica, 31, 133-154.

108. Tomialojc, L., Wesolowski, T. & Walankiewicz, W. (1984) Breeding bird community of a primaeval temperate forest (Bialowieza National Park, Poland). Acta ornithologica,

109. Wesolowski, T., Mitrus, C., Czeszczewik, D. & Rowinski, P. (2010) Breeding bird dynamics in a primeval temperate forest over thirty-five years: variation and stability in the changing world. Acta Ornithologica, 45, 209-232.

110. Wesolowski, T., Tomialojc, L., Mitrus, C., Rowinski, P. & Czeszczewik, D. (2002) The breeding bird community of a primaeval temperate forest (Bialowieza National Park, Poland) at the end of the 20th century. Acta ornithologica, 37, 27-45.

111. Wesolowski, T., Rowinski, P., Mitrus, C. & Czeszczewik, D. (2006) Breeding bird community of a primeval temperate forest (Bialowieza National Park, Poland) at the beginning of the 21st century. Acta Ornithologica, 41, 55-70.

112. Wesolowski, T., Czeszczewik, D., Hebda, G., Maziarz, M., Mitrus, C. & Rowinski, P. (2015) 40 years of breeding bird community dynamics in a primeval temperate forest (Bialowieza National Park, Poland). Acta Ornithologica, 50, 95-120.

113. Hall, G. A. (1984) A Long-Term Bird Population Study in an Appalachian Spruce Forest. The Wilson Bulletin, 96, 228–240.

114. Enemar, A., Sjöstrand, B. E., Andersson, G. Ö. & von Proschwitz, T. (2004) The 37-year dynamics of a subalpine passerine bird community, with special emphasis on the influence of environmental temperature and Epirrita autumnata cycles. Ornis Svecica, 14, 63–106.

115. Ruhl, H. A. & Rybicki, N. B. (2010) Long-term reductions in anthropogenic nutrients link to improvements in Chesapeake Bay habitat. Proceedings of the National Academy of Sciences, 107 (38), 16566–16570. doi:10.1073/pnas.1003590107.

116. Willis, T. "Hahei marine dataset (1997-2002), New Zealand fish". Institute of Marine Sciences, University of Portsmouth. Accessed 2016.

117. Lightfoot, D. "Small Mammal Exclosure Study (SMES)". Sevilleta Long Term Ecological Research Program. Available at: http://sev.lternet.edu/content/small-mammal-exclosure-study-smes-0, accessed 2016.

118. Monitoring Site 1000 Project, Biodiversity Center, Ministry of Environment of Japan (2015) "Monitoring site 1000 Coastal zone research - Tidal flat survey" (HIG01.zip, downloaded from http://www.biodic.go.jp/moni1000/findings/data/index_file_tidalflats.html). Accessed 2016.

119. Monitoring Site 1000 Project, Biodiversity Center, Ministry of Environment of Japan (2015) "Monitoring site 1000 Coastal zone research - Algae survey" (MOB02.zip, downloaded from http://www.biodic.go.jp/moni1000/findings/data/index_file_algalbeds.html). Accessed 2016.

120. Monitoring Site 1000 Project, Biodiversity Center, Ministry of Environment of Japan (2014) "Monitoring site 1000 Village survey - Bird survey data (2005-2012)" (SAT02.zip, downloaded from http://www.biodic.go.jp/moni1000/findings/data/index.html). Accessed 2016.

121. Monitoring Site 1000 Project, Biodiversity Center, Ministry of Environment of Japan (2014) "Monitoring site 1000 Village survey - Medium and large mammal survey data (2006-2012)" (SAT03zip, downloaded from http://www.biodic.go.jp/moni1000/findings/data/index.html). Accessed 2016.

122. Monitoring Site 1000 Project, Biodiversity Center, Ministry of Environment of Japan (2013) "Monitoring site 1000 Shorebird Survey" (ShorebirdsDatapackage2012.zip, downloaded from http://www.biodic.go.jp/moni1000/ findings/data/index.html). Accessed 2016.

123. How, R.A. (1998) Long-term sampling of a herpetofaunal assemblage on an isolated urban bushland remnant, Bold Park, Perth. Journal of the Royal Society of Western Australia, 81, 143-148.

124. Vrška, T., Král, K., Janík, D. & Adam, D. "Natural Forests of the Czech Republic". Available at: http://naturalforests.cz/research, accessed 2016.

125. Hsieh, C.-H. "Icthyoplankton data collected from Yenliao Bay in 6 stations northeast of Taiwan (1995-2000)". Ecoinformatics Lab, Institute of Oceanography National Taiwan University. Accessed 2016.

126. Kendeigh, S. C. (1982) Bird populations in east central Illinois: Fluctuations, variations, and development over a half-century. University of Illinois Press.

127. Fraser, W. (2014) “At-sea seabird censuses. Data on the species encountered (including marine mammals), their abundance, distribution and behavior. Data collected aboard cruises off the coast of the Western Antarctic Peninsula, 1993 - present”. Palmer Station Antarctica LTER. Available at: http://dx.doi.org/10.6073/pasta/e3871e749fa737dd94d5a269ac90e8ce, accessed 2016.

128. Svensson, S. (2006) Species composition and population fluctuations of alpine bird communities during 38 years in the Scandinavian mountain range. Ornis Svecica, 16(4), 183–210.

129. Barceló, C., Ciannelli, L., Olsen, E.M., Johannessen, T. & Knutsen, H. (2016) Eight decades of sampling reveal a contemporary novel fish assemblage in coastal nursery habitats. Global change biology, 22, 1155-1167.

130. Olsen, E. M., Carlson, S. M., Gjøsæter, J. & Stenseth, N. C. (2009) Nine decades of decreasing phenotypic variability in Atlantic cod. Ecology Letters, 12, 622–631. doi:10.1111/j.1461-0248.2009.01311.x

131. Rogers, L. A., Stige, L. C., Olsen, E. M., Knutsen, H., Chan, K.-S. & Stenseth, N. C. (2011) Climate and population density drive changes in cod body size throughout a century on the Norwegian coast. Proceedings of the National Academy of Sciences, 108(5), 1961–1966.

132. Stenseth, N. C., Bjørnstadf, O. N., Falck, W, Fromentin, J. M., Gjøsieter, J. & Gray, J. S. (1999) Dynamics of coastal cod populations: intra-and intercohort density dependence and stochastic processes. Proceedings of the Royal Society of London B: Biological Sciences, 266(1429), 1645–1654.

133. NIWA “The New Zealand Freshwater Fish Database”. Available at: https://www.niwa.co.nz/our-services/online-services/freshwater-fish-database, accessed 2016.

134. Hoey, A. “Karimunjawa WCS fish data”. Accessed 2016.

135. Hoey, A. “Aceh WCS fish 2010-16”. Accessed 2016.

136. Hoey, A. “Aceh WCS fish surveys”. Accessed 2016.

137. Zakharov, V. D. (1998) Biodiversity of bird population of terrestrial habitats in Southern Ural. Miass: IGZ, Ural Branch of Russian Academy of Sciences,158 p.

138. Berezovikov, N.N. (2004) The birds of settlements in Markakol Depression (Southern Altai). Russian Ornithological Journal, 249, 3-15.

139. Gavrilov, G. M. & Glebov, I. A. (2013) The composition and community structure of benthic fish in the economic zone of Russia Bering sea on the results of studies of “TINRO centr?” in 2005-2012 years. Modern problems of science and education, 11, 37–49.

140. Khoruzhiy, A. A. & Naydenko, S. V. (2014) Species structure and year-to-year dynamics of nekton biomass in the upper epipelagic layer of the Pacific waters at Kuril Islands in summer periods of the 2000s. Izv. TINRO, 176, 16–36.

141. Kuo, C.-Y., Yuen, Y.S., Meng, P.-J., Ho, P.-H., Wang, J.-T., Liu, P.-J., Chang, Y.-C., Dai, C.-F., Fan, T.-Y., Lin, H.-J., Baird, A. H. & Chen, C. A. (2012) Recurrent Disturbances and the Degradation of Hard Coral Communities in Taiwan. PLoS ONE, 7, e44364.

142. Thorn, S., Bässler, C., Bernhardt-Römermann, M., Cadotte, M., Heibl, C., Schäfer, H., Seibold, S. & Müller, J. (2016) Changes in the dominant assembly mechanism drive species loss caused by declining resources. Ecology Letters, 19, 163–170.

143. Thorn, S., Bässler, C., Gottschalk, T., Hothorn, T., Bussler, H., Raffa, K. & Müller, J. (2014) New insights into the consequences of post-windthrow salvage logging revealed by functional structure of saproxylic beetles assemblages. PLoS ONE, 9, e101757.

144. Thorn, S., Werner, S. A., Wohlfahrt, J., Bässler, C., Seibold, S., Quillfeldt, P. & Müller, J. (2016) Response of bird assemblages to windstorm and salvage logging - Insights from analyses of functional guild and indicator species. Ecological Indicators, 65, 142–148.

145. Neat, F. & Campbell, N. (2011) Demersal fish diversity of the isolated Rockall plateau compared with the adjacent west coast shelf of Scotland. Biological Journal of the Linnean Society, 104, 138–147. doi:10.1111/j.1095-8312.2011.01699.x.

146. Pombo, L. & Rebelo, J. E. (2002) Spatial and temporal organization of a coastal lagoon fish community-Ria de Aveiro, Portugal. Cybium, 26(3), 185–196.

147. Pombo, L., Elliott, M. I. & Rebelo, J. E. (2005) Environmental influences on fish assemblage distribution of an estuarine coastal lagoon, Ria de Aveiro (Portugal). Scientia Marina, 69(1), 143–159.

148. Pombo, L., Rebelo, J. E. & Elliott, M. (2007) The structure, diversity and somatic production of the fish community in an estuarine coastal lagoon, Ria de Aveiro (Portugal). Hydrobiologia, 587(1), 253–268.

149. Rebelo, J. E. (1992) The ichthyofauna and abiotic hydrological environment of the Ria de Aveiro, Portugal. Estuaries, 15(3), 403–413.

150. Muldavin, E. & Collins, S. (2003) Prescribed Burn Effect on Chihuahuan Desert Grasses and Shrubs at the Sevilleta National Wildlife Refuge, New Mexico: Species Composition Study 2004 to present. Sevilleta LTER. Available at: http://sev.lternet.edu/data/sev-166, accessed 2016.

151. Anderson, J., Vermeire, L. & Adler, P.B. (2011) Fourteen years of mapped, permanent quadrats in a northern mixed prairie, USA. Ecology, 92, 1703-1703.

152. Hogstad, O. (1993) Structure and dynamics of a passerine bird community in a spruce-dominated boreal forest. A 12-year study. Annales Zoologici Fennici, 30, 43-54.

153. Elmendorf, S.C. (2012) Global Tundra Vegetation Change –30 years of plant abundance data from unmanipulated and experimentally-warmed plots. Available at: http://www.polardata.ca, accessed 2017. CCIN reference number 10786.

154. Elmendorf, S.C., Henry, G.H., Hollister, R.D., Björk, R.G., Bjorkman, A.D., Callaghan, T.V., Collier, L.S., Cooper, E.J., Cornelissen, J.H. & Day, T.A. (2012a) Global assessment of experimental climate warming on tundra vegetation: heterogeneity over space and time. Ecology letters, 15, 164–175.

155. Elmendorf, S.C., Henry, G.H., Hollister, R.D., Björk, R.G., Boulanger-Lapointe, N., Cooper, E.J., Cornelissen, J.H., Day, T.A., Dorrepaal, E. & Elumeeva, T.G. (2012b) Plot-scale evidence of tundra vegetation change and links to recent summer warming. Nature Climate Change, 2, 453–457.

156. Elmendorf, S.C., Henry, G.H., Hollister, R.D., Fosaa, A.M., Gould, W.A., Hermanutz, L., Hofgaard, A., Jónsdóttir, I.S., Jorgenson, J.C. & Lévesque, E. (2015) Experiment, monitoring, and gradient methods used to infer climate change effects on plant communities yield consistent patterns. Proceedings of the National Academy of Sciences, 112, 448–452.

157. Rinnan, R., Stark, S. & Tolvanen, A. (2009) Responses of vegetation and soil microbial communities to warming and simulated herbivory in a subarctic heath. Journal of Ecology, 97, 788–800.

158. Ylänne, H., Stark, S. & Tolvanen, A. (2015) Vegetation shift from deciduous to evergreen dwarf shrubs in response to selective herbivory offsets carbon losses: evidence from 19 years of warming and simulated herbivory in the subarctic tundra. Global Change Biology, 21, 3696–3711.

159. Institute of Agricultural and Fisheries research (ILVO), Belgium (2016) Macrobenthos monitoring at long-term monitoring stations in the Belgian part of the North Sea between 1979 and 1999. Available at: http://dx.doi.org/10.14284/201, accessed 2016.

160. Institute of Agricultural and Fisheries research (ILVO), Belgium (2016) Macrobenthos monitoring at long-term monitoring stations in the Belgian part of the North Sea from 2001 on. Available at: http://dx.doi.org/10.14284/202, accessed 2016.

161. Institute of Agricultural and Fisheries research (ILVO), Belgium (2015) Epibenthos and demersal fish monitoring at long-term monitoring stations in the Belgian part of the North Sea. Available at: http://dx.doi.org/10.14284/54, accessed 2016.

162. Woods, K. D. (2014) Multi-decade biomass dynamics in an old-growth hemlock-northern hardwood forest, Michigan, USA. PeerJ, 2, e598.

163. Edelist, D., Rilov, G., Golani, D., Carlton, J.T. & Spanier, E. (2013) Restructuring the Sea: profound shifts in the world's most invaded marine ecosystem. Diversity and Distributions, 19, 69–77.

164. Souza, G.B.G. & Vianna, M. "Demersal fish hauls from Guanabara Bay, Brazil 2005-2015". Accessed 2017.

165. Hundt, R. (2001) Ökologisch-geobotanische Untersuchungen an den mitteldeutschen Wiesengesellschaften unter besonderer Berücksichtigung ihres Wasserhaushaltes und ihrer Veränderung durch die Intensivbewirtschaftung im Rahmen der Großflächenproduktion. Biosphärenreservat Rhön, Thüringen. Monografie, 3, 366.

166. Jandt, U. & Bruelheide, H. (2012) German vegetation reference database (GVRD). Biodiversity & Ecology, 4, 355-355.

167. Rocha, R. (2017) Tropical forest fragmentation: effects on the spatio-temporal dynamics of its bat communities. PhD Thesis, University of Lisbon, Lisbon, Portugal.

168. Rocha, R., López-Baucells, A., Farneda, F.Z., Groenenberg, M., Bobrowiec, P.E.D., Cabeza, M., Palmeirim, J.M. & Meyer, C.F.J. (2017) Consequences of a large-scale fragmentation experiment for Neotropical bats: disentangling the relative importance of local and landscape-scale effects. Landscape Ecology, 32, 31-45.

169. Sampaio, E.M., Kalko, E.K., Bernard, E., Rodríguez-Herrera, B. & Handley, C.O. (2003) A biodiversity assessment of bats (Chiroptera) in a tropical lowland rainforest of Central Amazonia, including methodological and conservation considerations. Studies on Neotropical fauna and environment, 38, 17-31.

170. Farneda, F.Z., Rocha, R., López-Baucells, A., Sampaio, E.M., Palmeirim, J.M., Bobrowiec, P.E., Grelle, C.E. & Meyer, C.F. (2018) Functional recovery of Amazonian bat assemblages following secondary forest succession. Biological Conservation, 218, 192-199.

171. Rocha, R., Ovaskainen, O., López-Baucells, A., Farneda, F.Z., Sampaio, E.M., Bobrowiec, P.E.D., Cabeza, M., Palmeirim, J.M. & Meyer, C.F.J. (2018) Secondary forest regeneration benefits old-growth specialist bats in a fragmented tropical landscape. Scientific Reports, 8, 3819.

# Original Data sources from TRY database

List of citations of the original data used from the TRY database. Data was also derived from otherwise unpublished datasets provided by TRY from M. Abedi, B. Amiaud, W. Bond, F. Chapin, E. Forey, N. Gross, A. Günther, M. Harzé, B. Hawkins, T. He, H. Jactel, K. Kramer, V. Lanta, M. Leishman, F.Lenti, B. Shipley, A. Totte, F. Valladares, and I. Wright


1. Adler P. B., D. G. Milchunas, W. K. Lauenroth, O. E. Sala and I. C. Burke (2004) Functional traits of graminoids in semi-arid steppes: a test of grazing histories. Journal of Applied Ecology 2004 41, 653–663

2. Auger, S., Shipley, B. (2012). : Interspecific and intraspecific trait variation along short environmental gradients in an old-growth temperate forest. Journal of Vegetation Science. DOI: 1111/j.1654-1103.2012.01473.x

3. Baraloto, C., C. E. T. Paine, L. Poorter, J. Beauchene, D. Bonal, A.-M. Domenach, B. Herault, S. Patino, J.-C. Roggy, and J. Chave. 2010. Decoupled leaf and stem economics in rainforest trees. Ecology Letters 13:1338-1347

4. Bocanegra-Gonzalez KT, Fermandez-Mendez F, Galvis-Jimenez, JF. (in press) Determinación de la resiliencia en bosques secundarios húmedos tropicales a través de la diversidad funcional de árboles en la región del Bajo Calima, Buenaventura, Colombia.

5. Bond-Lamberty, B., C. Wang, and S. T. Gower (2002), Above- and belowground biomass and sapwood area allometric equations for six boreal tree species of northern Manitoba, Can. J. For. Res., 32(8), 1441-1450.

6. Bragazza L (2009) Conservation priority of Italian alpine habitats: a floristic approach based on potential distribution of vascular plant species. Biodiversity and Conservation 18: 2823–2835.

7. Brendan Choat, Steven Jansen, Tim J. Brodribb, Herve Cochard, Sylvain Delzon, Radika Bhaskar, Sandra J. Bucci, Taylor S. Feild, Sean M. Gleason, Uwe G. Hacke, Anna L. Jacobsen, Frederic Lens, Hafiz Maherali, Jordi Martinez-Vilalta, Stefan Mayr, Maurizio Mencuccini, Patrick J. Mitchell, Andrea Nardini, Jarmila Pittermann, R. Brandon Pratt, John S. Sperry, Mark Westoby, Ian J. Wright & Amy E. Zanne (2012) Global convergence in the vulnerability of forests to drought. Nature 491:752-755 doi:10.1038/nature11688

8. Burrascano S, Copiz R, Del Vico E, Fagiani S, Giarrizzo E, Mei M, Mortelliti A, Sabatini FM, Blasi C (2015) Wild boar rooting intensity determines shifts in understorey composition and functional traits. COMMUNITY ECOLOGY 16(2) 244-253 DOI: 10.1556/168.2015.16.2.12

9. Butterfield, B.J. and J.M. Briggs. 2011. Regeneration niche differentiates functional strategies of desert woody plant species. Oecologia, 165:477-487.

10. Cadotte, M. W. 2017. Functional traits explain ecosystem function through opposing mechanisms. Ecology Letters 20:989-996

11. Campetella, G; Botta-Dukát, Z; Wellstein, C; Canullo, R; Gatto, S; Chelli, S; Mucina, L; Bartha, S (2011): Patterns of plant trait-environment relationships along a forest succession chronosequence. Agriculture, Ecosystems & Environment, 145(1), 38-48. doi:10.1016/j.agee.2011.06.025

12. Carswell, F. E., Meir, P., Wandelli, E. V., Bonates, L. C. M., Kruijt, B., Barbosa, E. M., Nobre, A. D. & Jarvis, P. G. 2000 Photosynthetic capacity in a central Amazonian rain forest. Tree physiology. 20, 3, p. 179-186 8 p.

13. Cavender-Bares, J., A. Keen, and B. Miles. 2006. Phylogenetic structure of floridian plant communities depends on taxonomic and spatial scale. Ecology 87:S109-S122.

14. Chacón-Madrigal, E., Wanek, W., Hietz, P., & S. Dullinger. 2018. Traits indicating a conservative resource strategy are weakly related to narrow range size in a group of neotropical trees. Perspectives in Plant Ecology, Evolution, and Systematics, https://doi.org/10.1016/j.ppees.2018.01.003

15. Ciocarlan V. (2009). The illustrated Flora of Romania. Pteridophyta et Spermatopyta. Editura Ceres, 1141 p (in Romanian).

16. Cornelissen, J. H. C. 1996. An experimental comparison of leaf decomposition rates in a wide range of temperate plant species and types. Journal of Ecology 84:573-582.

17. Cornelissen, J. H. C., B. Cerabolini, P. Castro-Diez, P. Villar-Salvador, G. Montserrat-Marti, J. P. Puyravaud, M. Maestro, M. J. A. Werger, and R. Aerts. 2003. Functional traits of woody plants: correspondence of species rankings between field adults and laboratory-grown seedlings? Journal of Vegetation Science 14:311-322.

18. Cornelissen, J. H. C., P. C. Diez, and R. Hunt. 1996. Seedling growth, allocation and leaf attributes in a wide range of woody plant species and types. Journal of Ecology 84:755-765.

19. Díaz, S., J. G. Hodgson, K. Thompson, M. Cabido, J. H. C. Cornelissen, A. Jalili, G. Montserrat-Martí, J. P. Grime, F. Zarrinkamar, Y. Asri, S. R. Band, S. Basconcelo, P. Castro-Díez, G. Funes, B. Hamzehee, M. Khoshnevi, N. Pérez-Harguindeguy, M. C. Pérez-Rontomé, F. A. Shirvany, F. Vendramini, S. Yazdani, R. Abbas-Azimi, A. Bogaard, S. Boustani, M. Charles, M. Dehghan, L. de Torres-Espuny, V. Falczuk, J. Guerrero-Campo, A. Hynd, G. Jones, E. Kowsary, F. Kazemi-Saeed, M. Maestro-Martínez, A. Romo-Díez, S. Shaw, B. Siavash, P. Villar-Salvador, and M. R. Zak. 2004. The plant traits that drive ecosystems: Evidence from three continents. Journal of Vegetation Science 15:295-304.

20. Everwand G, Fry, EL, Eggers T, Manning P (2014) Seasonal variation in the relationship between plant traits and grassland carbon and water fluxes. Ecosystems 17, 1095-1108

21. Falster DS, Remko A. Duursma, Masae I. Ishihara, Diego R. Barneche, Richard G. FitzJohn, Angelica Våhammar, Masahiro Aiba, Makoto Ando, Niels Anten, Michael J. Aspinwall, Jennifer L. Baltzer, Christopher Baraloto, Michael Battaglia, John J. Battles, Ben Bond-Lamberty, Michiel van Breugel, James Camac, Yves Claveau, Lluís Coll, Masako Dannoura, Sylvain Delagrange, Jean-Christophe Domec, Farrah Fatemi, Wang Feng, Veronica Gargaglione, Yoshiaki Goto, Akio Hagihara, Jefferson S. Hall, Steve Hamilton, Degi Harja, Tsutom Hiura, Robert Holdaway, Lindsay B. Hutley, Tomoaki Ichie, Eric J. Jokela, Anu Kantola, Jeff W. G. Kelly, Tanaka Kenzo, David King, Brian D. Kloeppel, Takashi Kohyama, Akira Komiyama, Jean-Paul Laclau, Christopher H. Lusk, Douglas A. Maguire, Guerric le Maire, Annikki Mäkelä, Lars Markesteijn, John Marshall, Katherine McCulloh, Itsuo Miyata, Karel Mokany, Shigeta Mori, Randall W. Myster, Masahiro Nagano, Shawna L. Naidu, Yann Nouvellon, Anthony P. OGrady, Kevin L. OHara, Toshiyuki Ohtsuka, Noriyuki Osada, Olusegun O. Osunkoya, Pablo Luis Peri, Any Mary Petritan, Lourens Poorter, Angelika Portsmuth, Catherine Potvin, Johannes Ransijn, Douglas Reid, Sabina C. Ribeiro, Scott D. Roberts, Rolando Rodríguez, Angela Saldaña-Acosta, Ignacio Santa-Regina, Kaichiro Sasa, N. Galia Selaya, Stephen C. Sillett, Frank Sterck, Kentaro Takagi, Takeshi Tange, Hiroyuki Tanouchi, David Tissue, Toru Umehara, Hajime Utsugi, Matthew A. Vadeboncoeur, Fernando Valladares, Petteri Vanninen, Jian R. Wang, Elizabeth Wenk, Richard Williams, Fabiano de Aquino Ximenes, Atsushi Yamaba, Toshihiro Yamada, Takuo Yamakura, Ruth D. Yanai, and Robert A. York. 2015. BAAD: a biomass and allometry database for woody plants. Ecology 96:1445.http://dx.doi.org/10.1890/14-1889.1

22. Fitter, A. H. and H. J. Peat 1994. The Ecological Flora Database. Journal of Ecology 82:415-425.

23. Fonseca, C. R., J. M. Overton, B. Collins, and M. Westoby. 2000. Shifts in trait-combinations along rainfall and phosphorus gradients. Journal of Ecology 88:964-977.

24. Frenette-Dussault, C., Shipley, B., Léger, J.F., Meziane, D. & Hingrat, Y. (2012). Functional structure of an arid steppe plant community reveals similarities with Grime's C-S-R theory. Journal of Vegetation Science 23: 208-222.

25. Giarrizzo E., Burrascano S., Chiti T., de Bello F., Leps J., Zavattero L., Blasi C. (2017) Re-visiting historical semi-natural grasslands in the Apennines to assess patterns of changes in plant species composition and functional traits. Applied Vegetation Science 20(2):247-258, doi: 10.1111/avsc.12288

26. Green, W. 2009. USDA PLANTS Compilation, version 1, 09-02-02. (http://bricol.net/downloads/data/PLANTSdatabase/) NRCS: The PLANTS Database (http://plants.usda.gov, 1 Feb 2009). National Plant Data Center: Baton Rouge, LA 70874-74490 USA.

27. Gutiérrez AG, & Huth A (2012) Successional stages of primary temperate rainforests of Chiloé Island, Chile. Perspectives in plant ecology, systematics and evolution. 14: 243– 256

28. Herz, K., Dietz, S., Haider, S., Jandt, U., Scheel, D. & Bruelheide, H. (2017): Drivers of intraspecific trait variation of grass and forb species in German meadows and pastures. – Journal of Vegetation Science 28: 705–716. Doi: 10.1111/jvs.12534. Herz, K., Dietz, S., Haider, S., Jandt, U., Scheel, D. & Bruelheide, H. (2017): Predicting individual plant performance in grasslands. – Ecology and Evolution 7: 8958–8965. DOI: 10.1002/ece3.3393

29. HILL, M.O., PRESTON, C.D. & ROY, D.B. (2004) PLANTATT - attributes of British and Irish Plants: status, size, life history, geography and habitats. Huntingdon: Centre for Ecology and Hydrology.

30. J. Fagúndez & J. Izco 2008. Seed morphology of two distinct species of Erica L. (Ericaceae). Acta Botanica Malacitana 33: 1-9

31. Kattge, J., W. Knorr, T. Raddatz, and C. Wirth. 2009. Quantifying photosynthetic capacity and its relationship to leaf nitrogen content for global-scale terrestrial biosphere models. Global Change Biology 15:976-991.

32. Kleyer, M., R. M. Bekker, I. C. Knevel, J. P. Bakker, K. Thompson, M. Sonnenschein, P. Poschlod, J. M. van Groenendael, L. Klimes, J. Klimesova, S. Klotz, G. M. Rusch, Hermy, M. , D. Adriaens, G. Boedeltje, B. Bossuyt, A. Dannemann, P. Endels, L. Götzenberger, J. G. Hodgson, A.-K. Jackel, I. Kühn, D. Kunzmann, W. A. Ozinga, C. Römermann, M. Stadler, J. Schlegelmilch, H. J. Steendam, O. Tackenberg, B. Wilmann, J. H. C. Cornelissen, O. Eriksson, E. Garnier, and B. Peco. 2008. The LEDA Traitbase: a database of life-history traits of the Northwest European flora. Journal of Ecology 96:1266-1274.

33. Kühn, I., W. Durka, and S. Klotz. 2004. BiolFlor - a new plant-trait database as a tool for plant invasion ecology. Diversity and Distribution 10 363-365.

34. Laughlin, D.C., P.Z. Fulé, D.W. Huffman, J. Crouse, and E. Laliberte. 2011. Climatic constraints on trait-based forest assembly. Journal of Ecology 99:1489-1499.

35. Li, Y. and Shipley, B. (2018) Community divergence and convergence along experimental gradients of stress and disturbance. Ecology, 99: 775-781. doi:10.1002/ecy.2162

36. Liebergesell M, Reu B, Stahl U, Freiberg M, Welk E, Kattge J, Cornelissen JHC, Penuelas J, Wirth C (2016) Functional Resilience against Climate-Driven Extinctions - Comparing the Functional Diversity of European and North American Tree Floras. PLoS ONE 11(2): e0148607. doi:10.1371/journal.pone.0148607

37. Lin Y-S, Medlyn BE, Duursma RA, Prentice IC, Wang H, Baig S, Eamus D, De Dios VR, Mitchell P, Ellsworth DS, De Beeck MO, Wallin G, Uddling J, Tarvainen L, Linderson M-L, Cernusak LA, Nippert JB, Ocheltree TW, Tissue DT, Martin-StPaul NK, Rogers A, Warren JM, De Angelis P, Hikosaka K, Han Q, Onoda Y, Gimeno TE, Barton CVM, Bennie J, Bonal D, Bosc A, Löw M, Macinins-Ng C, Rey A, Rowland L, Setterfield SA, Tausz-Posch S, Zaragoza-Castells J, Broadmeadow MSJ, Drake JE, Freeman M, Ghannoum O, Hutley LB, Kelly JW, Kikuzawa K, Kolari P, Koyama K, Limousin J-M, Meir P, Da Costa ACL, Mikkelsen TN, Salinas N, Sun W, Wingate L, (2015) Optimal stomatal behaviour around the world. Nature Climate Change 5(5): 459-464 DOI: 10.1038/NCLIMATE2550

38. Louault, F., V. D. Pillar, J. Aufrere, E. Garnier, and J. F. Soussana. 2005. Plant traits and functional types in response to reduced disturbance in a semi-natural grassland. Journal of Vegetation Science 16:151-160.

39. Manning, Houston and Evans 2009 Basic and Applied Ecology 10, 300-308

40. Marco Moretti and Colin Legg (2009) Combining plant and animal traits to assess community functional responses to disturbance. Ecography 32: 299 309. doi: 10.1111/j.1600-0587.2008.05524.x

41. Medlyn, B. E., F.-W. Badeck, D. G. G. De Pury, C. V. M. Barton, M. Broadmeadow, R. Ceulemans, P. De Angelis, M. Forstreuter, M. E. Jach, S. Kellomäki, E. Laitat, M. Marek, S. Philippot, A. Rey, J. Strassemeyer, K. Laitinen, R. Liozon, B. Portier, P. Roberntz, K. Wang, and P. G. Jarvis. 1999. Effects of elevated CO2 on photosynthesis in European forest species: a meta-analysis of model parameters. Plant, Cell and Environment 22:1475-1495.

42. MENCUCCINI M., 2003. The ecological significance of long distance water transport: short-term regulation and long-term acclimation across plant growth forms. Plant, Cell and Environment, 26:163-182.

43. Milla & Reich 2011 Annals of Botany 107: 455–465, 2011.

44. Miller JED, Ives AR, Harrison SP, Damschen EI (2018) Early- and late-flowering guilds respond differently to landscape spatial structure. J Ecol. 106:1033–1045. https://doi.org/10.1111/1365-2745.12849

45. Moles, A. T., D. S. Falster, M. R. Leishman, and M. Westoby. 2004. Small-seeded species produce more seeds per square metre of canopy per year, but not per individual per lifetime. Journal of Ecology 92:384-396.

46. Ordonez, J. C., P. M. van Bodegom, J. P. M. Witte, R. P. Bartholomeus, J. R. van Hal, and R. Aerts. 2010. Plant Strategies in Relation to Resource Supply in Mesic to Wet Environments: Does Theory Mirror Nature? American Naturalist 175:225-239.

47. Pahl, A.T., Kollmann, J., Mayer, A. & Haider, S. (2013): No evidence for local adaptation in an invasive alien plant: field and greenhouse experiments tracing a colonization sequence. Annals of Botany 112 (9): 1921-1930. DOI: 10.1093/aob/mct246

48. Paine CET, Amissah L, Auge H, Baraloto C, Baruffol M, Bourland N, Bruelheide H, Dainou K, de Gouvenain RC, Doucet J-L, Doust SJ, Fine PV a, Fortunel C, Haase J, Holl KD, Jactel H, Li X, Kitajima K, Koricheva J, Martinez-Garza C, Messier C, Paquette A, Philipson CD, Piotto D, Poorter L, Posada JM, Potvin C, Rainio K, Russo SE, Ruiz-Jaen M, Scherer-Lorenzen M, Webb CO, Zahawi RA & Hector A (2015) Globally, functional traits are weak predictors of juvenile tree growth, and we do not know why. Journal of Ecology, 103, 978–989. DOI: 10.1111/1365-2745.12401

49. Paula, S., M. Arianoutsou, D. Kazanis, Ç. Tavsanoglu, F. Lloret, C. Buhk, F. Ojeda, B. Luna, J. M. Moreno, A. Rodrigo, J. M. Espelta, S. Palacio, B. Fernández-Santos, P. M. Fernandes, and J. G. Pausas. 2009. Fire-related traits for plant species of the Mediterranean Basin. Ecology 90:1420.

50. Peco B., de Pablos I., Traba J. , & Levassor C. (2005) The effect of grazing abandonment on species composition and functional traits: the case of dehesa Basic and Applied Ecology, 6(2): 175-183

51. Prentice, I.C., Meng, T., Wang, H., Harrison, S.P., Ni, J., Wang, G., 2011. Evidence for a universal scaling relationship of leaf CO2 drawdown along a moisture gradient. New Phytologist 190: 169–180

52. Preston, K. A., W. K. Cornwell, and J. L. DeNoyer. 2006. Wood density and vessel traits as distinct correlates of ecological strategy in 51 California coast range angiosperms. New Phytologist 170:807-818.

53. Quested, H. M., J. H. C. Cornelissen, M. C. Press, T. V. Callaghan, R. Aerts, F. Trosien, P. Riemann, D. Gwynn-Jones, A. Kondratchuk, and S. E. Jonasson. 2003. Decomposition of sub-arctic plants with differing nitrogen economies: A functional role for hemiparasites. Ecology 84:3209-3221.

54. Rodrigues, A.V.; Bones, F.L.V.; Schneiders, A.; Oliveira, L.Z.; Vibrans, A.C.; Gasper, A.L. Plant Trait Dataset for Tree-Like Growth Forms Species of the Subtropical Atlantic Rain Forest in Brazil. Data 2018, 3, 16.

55. Royal Botanical Gardens KEW. 2008. Seed Information Database (SID). Version 7.1. Available from: http://data.kew.org/sid/ (May 2008).

56. Sandel, B., J. D. Corbin, and M. Krupa. 2011. Using plant functional traits to guide restoration: a case study in California coastal grassland. Ecosphere 2(2):art23. doi:10.1890/ES10-00175.1

57. Schweingruber, F.H., Landolt, W.: The Xylem Database. Swiss Federal Research Institute WSL Updated (2005)

58. Shiodera, S., J. S. Rahajoe, and T. Kohyama. 2008. Variation in longevity and traits of leaves among co-occurring understorey plants in a tropical montane forest. Journal of Tropical Ecology 24:121-133.

59. Shipley, B. and M. Parent. 1991. Germination Responses of 64 Wetland Species in Relation to Seed Size, Minimum Time to Reproduction and Seedling Relative Growth-Rate. Functional Ecology 5:111-118.

60. Smith, N. G. and Dukes, J. S. (2017), LCE: leaf carbon exchange data set for tropical, temperate, and boreal species of North and Central America. Ecology, 98: 2978. doi:10.1002/ecy.1992

61. Sophie Gachet, Errol Véla, Thierry Tatoni, 2005, BASECO: a floristic and ecological database of Mediterranean French flora. Biodiversity and Conservation 14(4):1023-1034

62. Spasojevic, M. J. and K. N. Suding. 2012. Inferring community assembly mechanisms from functional diversity patterns: the importance of multiple assembly processes. Journal of Ecology 100:652-661.

63. Swaine, E. K. 2007. Ecological and evolutionary drivers of plant community assembly in a Bornean rain forest. PhD Thesis, University of Aberdeen, Aberdeen.

64. Thuiller W - Traits of European Alpine Flora - Wilfried Thuiller - OriginAlps Project - Centre National de la Recherche Scientifique

65. Von Holle, B. and D. Simberloff. 2004. Testing Fox's assembly rule: Does plant invasion depend on recipient community structure? Oikos 105:551-563.

66. Williams, M., Y.E. Shimabokuro and E.B. Rastetter. 2012. LBA-ECO CD-09 Soil and Vegetation Characteristics, Tapajos National Forest, Brazil. Data set. Available on-line [http://daac.ornl.gov] from Oak Ridge National Laboratory Distributed Active Archive Center, Oak Ridge, Tennessee, U.S.A. http://dx.doi.org/10.3334/ORNLDAAC/1104

67. Wirth, C. and J. W. Lichstein. 2009. The Imprint of Species Turnover on Old-Growth Forest Carbon Balances - Insights From a Trait-Based Model of Forest Dynamics. Pages 81-113 in C. Wirth, G. Gleixner, and M. Heimann, editors. Old-Growth Forests: Function, Fate and Value. Springer, New York, Berlin, Heidelberg.

68. Wright JP, Sutton-Grier A (2012) Does the leaf economic spectrum hold within local species pools across varying environmental conditions? Functional Ecology 2012 doi: 10.1111/1365-2435.12001

69. Wright, I. J., D. D. Ackerly, F. Bongers, K. E. Harms, G. Ibarra-Manriquez, M. Martinez-Ramos, S. J. Mazer, H. C. Muller-Landau, H. Paz, N. C. A. Pitman, L. Poorter, M. R. Silman, C. F. Vriesendorp, C. O. Webb, M. Westoby, and S. J. Wright. 2007. Relationships among ecologically important dimensions of plant trait variation in seven Neotropical forests. Annals of Botany 99:1003-1015.

70. Wright, I. J., P. B. Reich, M. Westoby, D. D. Ackerly, Z. Baruch, F. Bongers, J. Cavender-Bares, T. Chapin, J. H. C. Cornelissen, M. Diemer, J. Flexas, E. Garnier, P. K. Groom, J. Gulias, K. Hikosaka, B. B. Lamont, T. Lee, W. Lee, C. Lusk, J. J. Midgley, M. L. Navas, U. Niinemets, J. Oleksyn, N. Osada, H. Poorter, P. Poot, L. Prior, V. I. Pyankov, C. Roumet, S. C. Thomas, M. G. Tjoelker, E. J. Veneklaas, and R. Villar. 2004. The worldwide leaf economics spectrum. Nature 428:821-827.

71. Wright, S. J., K. Kitajima, N. J. B. Kraft, P. B. Reich, I. J. Wright, D. E. Bunker, R. Condit, J. W. Dalling, S. J. Davies, S. Díaz, B. M. J. Engelbrecht, K. E. Harms, S. P. Hubbell, C. O. Marks, M. C. Ruiz-Jaen, C. M. Salvador, and A. E. Zanne. 2011 . Functional traits and the growth-mortality tradeoff in tropical trees. Ecology 91:3664-3674.

72. Zheng, W. 1983. Silva Sinica: Volume 1-4. China Forestry Publishing House, Beijing.
