# Supplementary material for: No pervasive relationship between species size and local abundance trends
Source: Nat Ecol Evol. 2021 Dec 30;6(2):140–4. doi: 10.1038/s41559-021-01624-8 (PMC8825279; doi:10.1038/s41559-021-01624-8)
Supplement: Supplementary file 2 — Reporting Summary [file 41559_2021_1624_MOESM2_ESM.pdf]

nature research

Corresponding author(s): J.C.D.Terry

Last updated by author(s): Aug 25, 2021

Reporting Summary

Nature Research wishes to improve the reproducibility of the work that we publish. This form provides structure for consistency and transparency in reporting. For further information on Nature Research policies, see our Editorial Policies and the Editorial Policy Checklist.

Statistics

For all statistical analyses, confirm that the following items are present in the figure legend, table legend, main text, or Methods section.

n/a	Confirmed	
☐	☒	The exact sample size (n) for each experimental group/condition, given as a discrete number and unit of measurement
☐	☒	A statement on whether measurements were taken from distinct samples or whether the same sample was measured repeatedly
☐	☒	The statistical test(s) used AND whether they are one- or two-sided<br>*Only common tests should be described solely by name; describe more complex techniques in the Methods section.*
☐	☒	A description of all covariates tested
☐	☒	A description of any assumptions or corrections, such as tests of normality and adjustment for multiple comparisons
☐	☒	A full description of the statistical parameters including central tendency (e.g. means) or other basic estimates (e.g. regression coefficient) AND variation (e.g. standard deviation) or associated estimates of uncertainty (e.g. confidence intervals)
☐	☒	For null hypothesis testing, the test statistic (e.g. F, t, r) with confidence intervals, effect sizes, degrees of freedom and P value noted<br>*Give P values as exact values whenever suitable.*
☒	☐	For Bayesian analysis, information on the choice of priors and Markov chain Monte Carlo settings
☒	☐	For hierarchical and complex designs, identification of the appropriate level for tests and full reporting of outcomes
☒	☐	Estimates of effect sizes (e.g. Cohen's d, Pearson's r), indicating how they were calculated

Our web collection on statistics for biologists contains articles on many of the points above.

Software and code

Policy information about availability of computer code

Data collection	Original data was downloaded from open databases as described in methods. Certain parts were downloaded from public APIs using R code publicly available in the project repository https://github.com/jcdterry/BioTIME_BodySize
Data analysis	All analysis code was written in R and is publicly available at https://github.com/jcdterry/BioTIME_BodySize and archived on Zenodo at https://doi.org/10.5281/zenodo.4745554

For manuscripts utilizing custom algorithms or software that are central to the research but not yet described in published literature, software must be made available to editors and reviewers. We strongly encourage code deposition in a community repository (e.g. GitHub). See the Nature Research guidelines for submitting code & software for further information.

Data

Policy information about availability of data

All manuscripts must include a data availability statement. This statement should provide the following information, where applicable:

- Accession codes, unique identifiers, or web links for publicly available datasets
- A list of figures that have associated raw data
- A description of any restrictions on data availability

Original sources of open-source datasets are listed in the methods. Processed data are available with analysis code at: https://github.com/jcdterry/BioTIME_BodySize and archived on Zenodo at https://doi.org/10.5281/zenodo.4745553

Field-specific reporting

Please select the one below that is the best fit for your research. If you are not sure, read the appropriate sections before making your selection.

☐ Life sciences ☐ Behavioural & social sciences ☒ Ecological, evolutionary & environmental sciences

For a reference copy of the document with all sections, see nature.com/documents/nr-reporting-summary-flat.pdf

Ecological, evolutionary & environmental sciences study design

All studies must disclose on these points even when the disclosure is negative.

Study description

We linked a large database of that collates studies of ecological community time series (BioTIME) to databases of body size traits, to detect if there are global patterns in the community dynamics of larger or smaller species.
Although we used millions of observations in our analysis, the fundamental statistical unit was the 'study' as tabulated in BioTIME. For each study we calculated the rank-correlation between a body-size trait of the species in that assemblage and changes in their abundance through time (which we termed 'tau'). Where a study covered a very large spatial extent, observations divided into grid cells, trait correlations calculated and then an overall average tau value for the study as a whole calculated. We tested three approaches to transforming the community abundance data, as detailed in the methods.
Trends in this set of 'tau' correlation values was then tested using simple statistical tests. Firstly, to test if the mean was different to a null expectation (effectively zero). For this we generated null-data by randomising available trait data within each assemblage and carrying out permutation tests. Secondly, to detect if 'tau' was influenced by a suite of properties for the study (e.g. latitude or duration of the study) we fit linear models, transforming the predictor variables if necessary.
We tested 6 body size traits, which largely correspond to different ecological guilds. We treated each of the 6 guild/trait combinations as independent samples. However, we note that a number of studies had sufficient trait data to appear in multiple tests, as detailed in the main text methods.

Research sample

Community dynamics data was sourced from the BioTIME database (http://biotime.st-andrews.ac.uk/downloadArea.php). This was selected as it is the largest and most comprehensive such database, that has given considerable insight into global biodiversity trends.
Trait databases were selected based on scale and the species groups contained within BioTIME and are cited in the methods section

Sampling strategy

We sought to maximise our sample size by using all data available that was of sufficient quality. The number of studies is moderate (and clearly displayed in the dotplots). We directly present the null distribution with which we compare each mean and the number of samples in the dotplots, so readers should be able to comprehend the statistical power available to us.

Data collection

Original data was collected from a huge number of authors, as detailed in the database references. Data processing was all carried out by JCDT as outlined in the methods and presented in the publicly available code.

Timing and spatial scale

Our data filtering to exclude short time series (less than 10 years span) in the BioTIME database was pre-determined as offering some chance of detecting major trends.
The datasets used are global in scope, although the distribution is skewed. The spatial grain of each study (as reported in BioTIME) is included in our results. The spatial scale of assignment to cells of dispersed studies was based on previous work and corresponds to the larges single site studies.

Data exclusions

Data exclusions fall into two categories. Firstly, some data was excluded due to being from time series that were too short or from too simple a community to be useful. There are a number of stages to this, and are detailed in our methods. Secondly, the process of cleaning 10'000s of names necessitates automation that cannot be comprehensive, and often has to err on the side of not matching uncertain fields and hence excluding data by default. With considerable further manual work, a moderate number of more species listed in BioTIME could be linked to trait values, but this is unlikely to significantly change the key results (study-level trait completeness was not predictive)

All such decisions are noted within the analysis code and all significant such choices are listed in the methods.

Reproducibility

The whole analysis is fully reproducible based on publicly available code and datasets.

Randomization

Not relevant – we used all data available and did not assign samples to treatments

Blinding

This was largely not relevant. Trait databases were constructed without reference to the dynamics of the species in question to mitigate possible biases in effort applied to assigning trait values.

Did the study involve field work? ☐ Yes ☒ No

Reporting for specific materials, systems and methods

We require information from authors about some types of materials, experimental systems and methods used in many studies. Here, indicate whether each material, system or method listed is relevant to your study. If you are not sure if a list item applies to your research, read the appropriate section before selecting a response.

Materials & experimental systems

n/a	Involved in the study
☒	☐ Antibodies
☒	☐ Eukaryotic cell lines
☒	☐ Palaeontology and archaeology
☒	☐ Animals and other organisms
☒	☐ Human research participants
☒	☐ Clinical data
☒	☐ Dual use research of concern

Methods

n/a	Involved in the study
☒	☐ ChIP-seq
☒	☐ Flow cytometry
☒	☐ MRI-based neuroimaging
