# Supplementary material for: No pervasive relationship between species size and local abundance trends
Source: Nat Ecol Evol. 2021 Dec 30;6(2):140–4. doi: 10.1038/s41559-021-01624-8 (PMC8825279; doi:10.1038/s41559-021-01624-8)
Supplement: Supplementary file 3 — Peer Review Information. [file 41559_2021_1624_MOESM3_ESM.pdf]

natureresearch

# Peer Review Information

**Journal:** Nature Ecology & Evolution
**Manuscript Title:** No pervasive relationship between species size and local abundance trends
**Corresponding author name(s):** J. Christopher D. Terry

## Editorial Notes:

| | |
|---|---|
| **Redactions – published data** | Parts of this Peer Review File have been redacted as indicated to remove third-party material. |

## Reviewer Comments & Decisions:

**Decision Letter, initial version:**

17th June 2021

*Please ensure you delete the link to your author homepage in this e-mail if you wish to forward it to your co-authors.

Dear Dr Terry,

Your Brief Communication, "No pervasive size trend in global community dynamics" has now been seen by three reviewers. You will see from their comments copied below that while they find your work of potential interest, they have raised quite serious concerns that must be addressed.

In particular, Reviewers 1 and 3 highlight substantial issues with the rank-based analytical approach. We feel that the conclusions are not strong enough at this stage to warrant publication in Nature Ecology & Evolution, and it appears that an overhaul of the analyses would be necessary to make the results more robust. Although we will consider a revised manuscript, please know we are unlikely to send it back to the reviewers unless all of their concerns are thoroughly addressed.

If you choose to revise your manuscript taking into account all reviewer and editor comments, please highlight all changes in the manuscript text file.

We are committed to providing a fair and constructive peer-review process. Please do not hesitate to contact us if there are specific requests from the reviewers that you believe are technically impossible

or unlikely to yield a meaningful outcome.

If revising your manuscript:

* Include a “Response to reviewers” document detailing, point-by-point, how you addressed each referee comment. If no action was taken to address a point, you must provide a compelling argument. This response will be sent back to the referees along with the revised manuscript.

* If you have not done so already we suggest that you begin to revise your manuscript so that it conforms to our Brief Communication format instructions at http://www.nature.com/natecolevol/info/final-submission. Refer also to any guidelines provided in this letter.

* Include a revised version of any required reporting checklist. It will be available to referees (and, potentially, statisticians) to aid in their evaluation if the manuscript goes back for peer review. A revised checklist is essential for re-review of the paper.

Please use the link below to submit a revised paper:

***[REDACTED]***

<strong>Note:</strong> This URL links to your confidential home page and associated information about manuscripts you may have submitted, or that you are reviewing for us. If you wish to forward this email to co-authors, please delete the link to your homepage.

If you wish to submit a suitably revised manuscript we would hope to receive it within 6 months. If you cannot send it within this time, please let us know. We will be happy to consider your revision so long as nothing similar has been accepted for publication at Nature Ecology & Evolution or published elsewhere.

Nature Ecology & Evolution is committed to improving transparency in authorship. As part of our efforts in this direction, we are now requesting that all authors identified as ‘corresponding author’ on published papers create and link their Open Researcher and Contributor Identifier (ORCID) with their account on the Manuscript Tracking System (MTS), prior to acceptance. This applies to primary research papers only. ORCID helps the scientific community achieve unambiguous attribution of all scholarly contributions. You can create and link your ORCID from the home page of the MTS by clicking on ‘Modify my Springer Nature account’. For more information please visit please visit <a href="http://www.springernature.com/orcid">www.springernature.com/orcid</a>.

Please do not hesitate to contact me if you have any questions or would like to discuss the required revisions further.

Thank you for the opportunity to review your work.

***[REDACTED]***

Reviewer expertise:

Reviewer #1: Macroecology, biodiversity declines

Reviewer #2: Population trends

Reviewer #3 : Macroecology, biodiversity declines

Reviewers' comments:

Reviewer #1 (Remarks to the Author):

This review is for Terry et al. "No pervasive size trend in global community dynamics", where the authors examined the evidence that larger body sizes are disproportionately declining in abundance. Specifically they looked at correlations between body size and changes in rank within community assemblages, using the BioTIME database, along with a number of trait databases. I thought the purpose of the paper was interesting and sufficiently broad to be of interest to Nature Ecol Evol. However, I question a number of elements in the analyses.

First, I wonder if the metrics used are insensitive. Particularly, the use of ranks might be insensitive to changes in relative abundance, even if they are occurring. I might expect that larger animals tend to have lower abundances. If this is the case, even if their absolute abundances decrease even more, it might not change the rank, but could nonetheless be important. What were the original ranks? Were larger species more often having lower abundances?

I wonder if one might target assemblages where absolute changes are actually occurring (other ones might add noise). The question could be respecified as "are changes in assemblages due to a disproportionate decline of large bodied species"? Was there a relation with magnitude of turnover?

Do you predict larger bodied species to have more positive or negative relations (I thought negative, right? larger bodies, greater decline in rank). I ask because Fig 1(iv) shows a positive relation. I also notice that most of the mean tau values were positive (albeit most non-significant). Is this expected?

the authors state that they generated 100 randomized tau values for each assemblage (although in Fig 2, they indicate they used 50 randomizations), and then performed two-sample t-tests to see whether the real mean differed from the null. It is not clear how this was done - was a t-test conducted for each of the 100 sets of randomizations? Which t-test was used in this case? or was the mean t-value used? Regardless, applying two sample t-tests to simulated (randomized) sets, treating them as if they were real data, probably isn't the best approach, given the arbitrary nature of how many simulated sets one generates. Instead, why not run 1000 or 10000 randomized sets, calculate tau and just see whether the empirical tau is greater or less than 95% of the sets? I think it is more powerful. I also wonder if power is lost, treating each assemblage as a single data point, and whether formal meta-analytic procedures might be possible. Finally, why are 2-tailed tests used? You have a clear expectation of larger body sizes declining more.

Minor points:

The authors binned widespread species into 10km grid cells. I wonder if this causes issues of non-independence.

The authors attribute the positive relation for amniote studies to anthropogenic dispersal limitations (affects smaller bodies animals more). Could the authors explain this more? Is anthropogenic dispersal limitations increasing during the time periods of the studies?

Reviewer #2 (Remarks to the Author):

The manuscript: ”No pervasive size trend in global community dynamics” investigates whether there is a global trend of decline in species body size. For that, the relationship between size traits and changes in abundance rank over time is analysed for several assemblages worldwide.

The study is well designed and make use of the BioTime database and several trait databases. The text is concise and well written and the figures and supporting materials are informative. The authors did a good job discussing the caveats of using such databases and performing such large-scale analyses. The conclusions are supported by the results and, in my opinion, the manuscript is of potential interest to the readers of this journal.

I appreciate that the authors explicitly mention that insects are not included in the analysis. I recommend to also discuss bias in the distribution of data across realms: are freshwater assemblages included in the study? This is relevant to the scope of the manuscript, as freshwater megafauna is reported as declining globally, see e.g. He, F., Zarfl, C., Bremerich, V., David, J. N., Hogan, Z., Kalinkat, G., ... & Jähnig, S. C. (2019). The global decline of freshwater megafauna. Global change biology, 25(11), 3883-3892.

From Extended Data Figure 1, it seems that the majority of time series are less than 20-years long. It could be that the decline or extinction of large-bodied species happened before the beginning of the time series. In that case, the time series cannot track the changes in size:ranks relationships. I recommend to discuss this issue.

Here some additional comments:

Line 16: delete “is”.

Figure 1b: The symbols are too small and difficult to distinguish. Please, increase their size to improve readability.

Line 119: The caption of figure 2 says that “Blue density plots show the distribution of 50 randomisations”. I wonder why 50 randomizations and not 100, as 100 were used to create the null distribution according to lines 47 and 185.

Line 177, “very small β values”: Can you please define what “very small” means?

Line 191: delete “that”.

Lines 189-194: From Extended Data Figure 1, it seems that there are a few outliers in the raw data and skewed distributions. Were the explanatory variables transformed?

Reviewer #3 (Remarks to the Author):

This study assesses whether temporal species turnover in community time series results in functional changes related to species size. This is an interesting and relevant question because numerous studies have pointed out size-related vulnerability to anthropogenic threats, and other have concluded that communities will be increasingly dominated by small species. I think this can be an important contribution with potentially interesting results. However, overall I felt a bit dissatisfied with the study. My main concerns relate to the analyses and hypotheses.

1) I don't think using ranks is a good idea. Using ranks makes the analysis quite insensitive to changes, and does not focus on changes per se but rather to reversals in species relative abundances. A species can remain stable and another may be declining, but in a matter of 15 years or so the rank will likely remain stable. Take a simplistic example of a community with two species, a mouse and a tiger. If the tiger decreases or increases while the mouse remain stable you won't see any change in the ranks, as the tiger will always be rarer than the mouse. Similarly, if the mouse decreases and the tiger increases, the rank will also remain invariant as these two species differ by orders of magnitude in their population density.
So at present, I'm not convinced by the conclusions made by the authors.
I see two possible options. One would consist in a linear model with abundance as dependent variable and body mass and time as predictors. The interaction between the two should inform on size-related changes. In such a model you could also include a number of potentially confounding variables (e.g. those assessed in Fig. 1 in extended data), which would make results more robust to these factors than a simple visual inspection. Obviously this requires some thinking about random intercepts/slopes etc..
A second option I can think of, is focusing on some community weighted average of body size and how this changes over time. You can still analyse this using a linear model.

2) The authors start by explaining that studies focusing on time-series have observed species turnover, and others have claimed different trends for species with different sizes. This is true, however, most of the literature on this (at least the one I know) relates to endothermic vertebrates, and here the authors analyse several different taxa including plants. I'd like to see more space dedicated to what is known or not known, how this knowledge is taxonomically biased, and so what can we expect from these results. Also note that even within taxa, authors have pointed out different size-trends (e.g. Ripple et al. 2017 PNAS, or Santini et al. 2021 Ecol Lett cited in the text). Currently the paper jumps into the methods and results too soon, I think the authors need to dig deeper in the literature and provide an overview of what is known and could be expected.

11-12: This comes a bit out of the blue in the abstract, both because specifically focused on body size (but it starts refering to traits in general), and because of the expectation small and large may do better/worse. Perhaps an introductory sentence on common perception/previous studies is worth adding on this

L. 19-20: See also
González-Suárez, M., Gómez, A., & Revilla, E. (2013). Which intrinsic traits predict vulnerability to extinction depends on the actual threatening processes. Ecosphere, 4(6), 1-16.

For how threats-vulnerability is related to spefici traits in mammals

Ripple, W. J., Wolf, C., Newsome, T. M., Hoffmann, M., Wirsing, A. J., & McCauley, D. J. (2017). Extinction risk is most acute for the world's largest and smallest vertebrates. Proceedings of the National Academy of Sciences, 114(40), 10678-10683.

For how vertebrates body mass relates to extinction risk (note that here they conclude both small and large are at higher risk for different reasons).

Cooke, R. S., Eigenbrod, F., & Bates, A. E. (2019). Projected losses of global mammal and bird ecological strategies. Nature communications, 10(1), 1-8.

Here the authors projected community changes into the future for birds and mammals

In general, you here are assessing entire communities, but as far as I know the literature on body-size related trends is mostly on mammals, and secondarily on vertebrates. The expectation for entire communities or different taxa needs to be better grounded in the literature

Line 28: is there any evidence that larger/smaller plants do better/worse?
I only remember this paper

de Jonge, M. M., Hilbers, J. P., Jongejans, E., Ozinga, W. A., Hendriks, A. J., & Huijbregts, M. A. (2018). Relating plant height to demographic rates and extinction vulnerability. Biological Conservation, 220, 104-111.

Line 50: positive relationship between body mass and "positive/negative"(?) trends?

Line 53: "predict" is a big word. I can imagine they do not predict them, but did they influence them? Did you find any relationship even if weak?
It would really help to add a loess fit on top of the scatterplots for better interpretation (but see my comment above on the need of a more robust way to account for these factors on the results). Also, it seems to me that some variables may benefit from transformation, e.g. species richness

Line 62-63: Is the direction of these changes consistent? Or no consistent pattern is observed?

65-67: Density is strongly negatively related to body mass, so habitat area is a bigger issue for large than small species

Line 70-71: Sometime the description/interpretation of the results is hard to follow, here and elsewhere I suggest explaining the pattern in words rather than referring to t and its statistical distribution.

90: This should be "meta-analyses", not "metanalyses" .
Note, however, that most of these analyses are not proper meta-analyses in sensu strictu, just

statistical analyses

Fig. 1 What are the coloured lines in b? It is a bit confusing, I'd only show the points. Also, include symbol legend in the figure for simplicity

139: Honestly, I would not trust the GBIF backbone taxonomy, gbif is full of misspelled names, synonyms, subspecies treated or not treated as species, etc.
Why not using catalogue of life in taxize?

Fig. 2 It may help to add % >0 for better interpretation

Line 218: I miss a rationale for it. Why do we need a null model? Why should we expect null model to deviate from no change?

One final point to take in consideration. The time series are, in most cases, quite short to detect such a trend. We may be able to detect changes in species with rapid life histories, but are we capable of detecting changes in species with slow life histories (the large ones)? This is something that is worth discussing and perhaps take into account in the analysis if possible

**Author Rebuttal to Initial comments**

*This review is for Terry et al. "No pervasive size trend in global community dynamics", where the authors examined the evidence that larger body sizes are disproportionately declining in abundance. Specifically they looked at correlations between body size and changes in rank within community assemblages, using the BioTIME database, along with a number of trait databases. I thought the purpose of the paper was interesting and sufficiently broad to be of interest to Nature Ecol Evol. However, I question a number of elements in the analyses.*

We are heartened to read that reviewer found the paper of interest for the NEE audience. We respond to the particular concerns below.
*First, I wonder if the metrics used are insensitive.*

*Particularly, the use of ranks might be insensitive to changes in relative abundance, even if they are occurring.*

*I might expect that larger animals tend to have lower abundances. If this is the case, even if their absolute abundances decrease even more, it might not change the rank, but could nonetheless be important. What were the original ranks? Were larger species more often having lower abundances?*

The reviewer puts forward two potential issues with rank-based approaches. Although both are valid possible problems, they are not a cause for fundamental concern in our analysis with this dataset. As we have said above, we no longer present a rank-based approach as ou**r 'principle' analysis, but do believe that is helpful to** present it as an alternative complementary approach that gives very similar results (Extended data figure 4 + 5).

An unfortunate feature of the BioTIME dataset is that there is no clear standardisation of sampling intensity through time. While in many of the studies sampling has been consistent, this is not be guaranteed. In this context, the ranking approach has the advantage of directly accommodating varying sampling effort or whole-community responses to environmental variability. However, this resilience does come at the expense of a degree of loss of information. For this reason, we believe, both rank-based and non-rank-based methods are complimentary, each having advantages and disadvantages. By including multiple methods, we believe our manuscript is now considerably stronger.

*Small abundance changes not being reflected in rank changes*

In response to the reviewer's first specific point, a key contextualising feature here is that rank changes are very frequent in the dataset. While it is true that it is possible that abundance changes may not necessarily translate into rank changes, in practice, they commonly do.

Where there is a potential issue, is that certain species at the very top of the ranking are less likely to change their ranking (being both dominant and as they can only move in one direction). However, this only affects a very small proportion of the species in the system. While theoretically there could be a corresponding 'edge' effect at the bottom of the ranking, in practice there is so much turnover at the very bottom (rare species being irregularly observed) in most systems that this is not visible. Even in the extreme case where a handful of abundant species maintain their dominant position, this still leaves the majority of non-dominant species (whose abundances are more similar) free to exchange rank positions further down the hierarchy.

*Biases deriving from size-abundance patterns.*

The reviewer's expectation that there could be an existing skewed pattern of traits that interacts with these choices (complicating the interpretation of tau) is a fair and widespread one. As it turns out, the relationship between average relative abundance rank and relative trait rank is very weak across the whole dataset (see figure below). This is not wholly unexpected: literature expectations suggest *local* size-density relationships (such as we are looking at here) are often weak (White et al 2007).

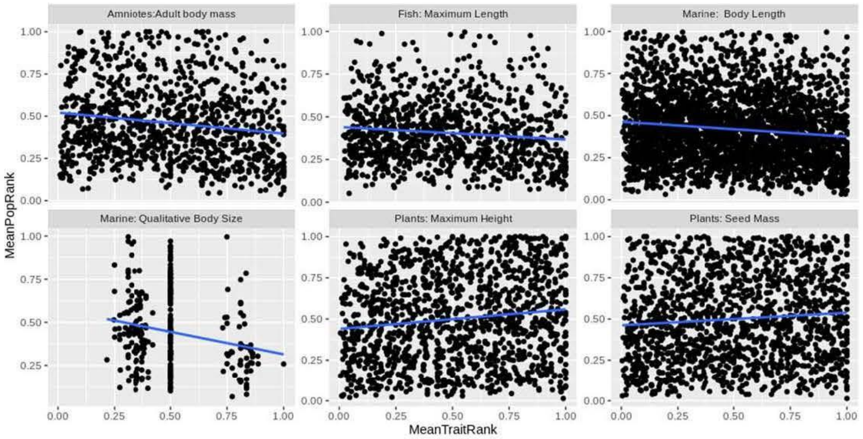


[N.B Here each dot in the above figure represents a single species within a study. X-axis is the mean trait rank within each assemblage, the y-axis the mean population rank across all assemblages. Figure is facetted by trait – where a species has multiple categories of trait data, it would appear multiple times]

*Overall sensitivity*

In order to address our question, essentially we are faced with the necessity to distil complex community level dynamics with patchy data down into a single number for each study. It would be fairly straightforward to construct artificial scenarios where a metric does or does not act in congruence with some other measure of trait-community dynamics - there are so many dimensions that could be tweaked. As such, quantifying the sensitivity of a metric of community biodiversity change is extremely challenging, and indeed not particularly helpful. It won't be news to any of the readers that there a multitude of valid ways to assess shifts of traits in communities.

That said, we can show that all three of the metrics we include in the revised manuscript are sensitive enough to pick up trends within individual studies in our dataset. In our revised manuscript, we highlight in figure 2 those studies that are individually significant, to make this point more clear. Further, as our tau is a two-sided metric, we make use of its sign, not just a magnitude (which is not straightforward to uniquely define) - we observe a more less even balance in positive and negative tau values. For these reasons, we do not believe the **'sensitivity' as such of the tau metric is a fundamenta**l cause for concern about our main conclusions. Instead, we believe a more valuable discussion, is about whether the signals that the metric is able to detect are in some way illuminating and provide a useful summary of the complex underlying dynamics.

*I wonder if one might target assemblages where absolute changes are actually occurring (other ones might add noise). The question could be respecified as "are changes in assemblages due to a disproportionate decline of large bodied species"? Was there a relation with magnitude of turnover?*

This question would certainly be an interesting analysis**, but we don't believe it is necessary to narrow the** scope of the main question to this degree by excluding certain studies. As we discussed above, nearly all assemblages showed considerable changes through time, both in abundances and in abundance rank, although there are some identifiable differences in the rate of turnover (Antão et al 2020). A sizeable proportion of species can be identified as clear winners or losers (see also Dornelas et al 2019), with clearly identifiable increases or decreases through time.

Our additional, species-based analysis, that relates the relative size of a species within an assemblage to **whether it can be categorised as a clear 'winner' or a 'loser' (Extended Data Figure 3), comes quite close to** answering this suggested question. We find that a species being relatively large for its community does not influence its chance of being a winner or loser. Although the scope is slightly different, this would indicate that there is no disproportionate decline of larger species.

**To answer the reviewer's final question (the relationship** between magnitude of turnover and our tau) we have **looked into this, and don't see a consistent trend acro**ss the different traits that would indicate a cause for concern that underlying turnover rate is causing an artefact (see figure below). Since the reviewer query was based on the rank-based metric, for consistency we show here a rank-based, metric of turnover derived from Collins et al (2008). Because the rigorous analysis of turnover is a considerable undertaking its own right (e.g. Antão et al 2020), we believe a full analysis would be outside the scope of our aims of the paper.

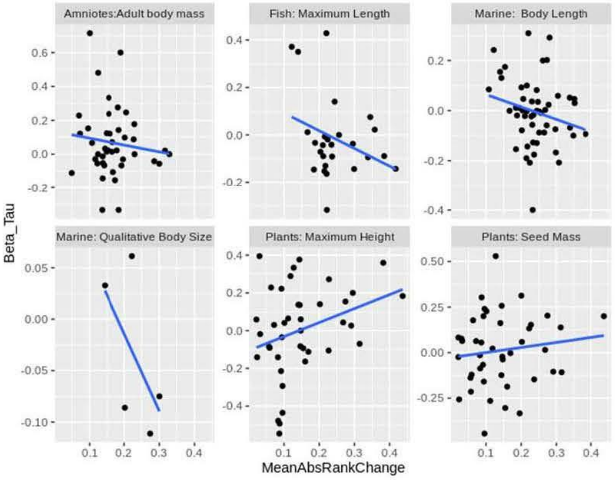

Here each dot represents one study. Y-axis = study tau value, calculated using the original rank-based approach. X-axis = the average rank shift observed in a community timeseries as:

$$RS = \frac{1}{n}\sum_{i=1}^{n}\sum_{t=1}^{L}\frac{|R_{i,t+1} - R_{i,t}|}{L}$$

where R is the relative rank of each species at each sample step, t is the sample index, L is the number of transitions (i.e. number of samples -1), n is the number of species in the assemblage, i is the species index. We note that this is only a quite rough analysis, since it is hard to know if the metric is capturing mostly small fluctuations, differences in observation error or consistent trends. It is also worth noting that the sampling period is sometimes irregular in some assemblages.

*Do you predict larger bodied species to have more positive or negative relations (I thought negative, right? larger bodies, greater decline in rank). I ask because Fig 1(iv) shows a positive relation. I also notice that most of the mean tau values were positive (albeit most non-significant). Is this expected?*

Indeed, if asked before we conducted the analyses, we would have expected a negative relationship to be more likely, since this is the general literature expectation (as we discuss the early parts of the text). The number of studies with positive tau values is notable. It is a surprising result, and part of why we believe the results are of interest to a wide audience.

Fig 1(iv) was just a single illustrative example of the method that happened to show a positive trend for a particular assemblage. It was selected essentially at random (when designing the figure we rejected a couple of alternative assemblages that had a large number of species, that made the figures challenging to interpret). In any case, we now no longer include this subfigure in our revised manuscript to free up space for splitting the map.

*the authors state that they generated 100 randomized tau values for each assemblage (although in Fig 2, they indicate they used 50 randomizations), and then performed two-sample t-tests to see whether the real mean differed from the null. It is not clear how this was done - was a t-test conducted for each of the 100 sets of randomizations? Which t-test was used in this case? or was the mean t-value used? Regardless, applying two sample t-tests to simulated (randomized) sets, treating them as if they were real data, probably isn't the best approach, given the arbitrary nature of how many simulated sets one generates. Instead, why not run 1000 or 10000 randomized sets, calculate tau and just see whether the empirical tau is greater or less than 95% of the sets? I think it is more powerful.*

Firstly, apologies for the mistake in the figure caption, we used 100 randomisations per assemblage-trait combination in the original analysis, not 50.

We have been happy to switch over to a fully randomisation approach as the reviewer suggests. The results are fundamentally unchanged. Our initial choice of t-tests was based on the hope that they would be broadly familiar, but clearly the additional adjustments we had to add meant we lost any advantage on this front. There is an argument that within the frequentist approach it is imperative to stick with the first test planned and carried out (the 2-sample t-test) on the basis that it was the originally planned test, and we would be happy to revert if thought appropriate. However, our results do not critically depend on the choice of test. A final alternative would be to include both tests, but this seems unlikely to add any additional clarity or insight.

We now represent the range of mean values from the randomisations as an interval under the dot-plots, and **the observed mean. We calculate 'exact' p-values from** an empirical distribution calculated from a set of **10'000 permutations of the trait data within each asse**mblage. We also use the same set of randomisations to **individually identify studies that show the 'study-level' significance, and highlight these in the dot plots.**

At this point, we reiterate that although the mean tau value of the amniote body mass trait is identified as being 'significantly' different to zero, we don't believe that this is a particular aspect of the results that should be overly-trumpeted (compared to the observation of a large overall spread of results). The BioTIME database has too many biases within it for a marginal divergence across studies from zero to be a signifier of a true directional trend given the wide variability. There is also a question of multiple comparisons – although the tests are largely independent, the fact that we did test six traits should be factored into the interpretation of the p-values (although we would argue that including an explicit Bonferroni-type correction here is not helpful). We have added some additional notes on this in the main text of our revised manuscript.

Although we no longer use the t-test approach, to answer the reviewer's questions: The objective of our overall summary statistics is simply to ask 'is the mean of the study-$\tau$'s different to zero within each trait', for which a one-sample t-test would be the classic statistical test. However, our case was complicated by the potential that the variance of the observed sample could differ from a random expectation. To correct for this, we used a two-sample t-test, where the 1$^{st}$ sample is the observed data and the 2$^{nd}$ sample is much 100x larger and generated from permutating the trait-ranks within each assemblage before calculating $\tau$. This second (null) sample has a mean that is essentially zero. While as the reviewer points out our choice of the number of randomisations was arbitrary, the estimation of the variance of the reference set converged rapidly enough, and with the reference set 100x larger than the observed dataset, further increase in size contributes very little further change to the effective degrees of freedom as calculated within the Welch's t-test. We also note that the one-sample t-test we included in the original SI gave near identical results.

*I also wonder if power is lost, treating each assemblage as a single data point, and whether formal meta-analytic procedures might be possible.*

This is a pertinent point, and one we considered when planning our analysis. A formal meta-analysis is appealing and in principle would be an improvement on our relatively simple approach. However, we believe the data (while extensive) would be insufficient to justify such an analysis. The motivation to conduct a formal metanalysis would be to try to improve in some way how different aspects of the data should be weighted. However, we believe our simple approach is considerably more justifiable.

It would seem reasonable to place extra weight on 'larger' studies, but the size of a study is multidimensional, encompassing the number of species, the taxonomic breadth of the study, the geographic scope, the size of individual samples, the temporal extent, the intensity and error of the sampling etc. When we examined a suite of these such study-properties, there was no clear indication that they are consistently related to our tau measure across different traits. It seems most reasonable to us to trust the original authors of the studies in the BioTIME database that they selected appropriate bounds for each focal assemblage.

A case could also be made to attempt to 'rebalance' the dataset, placing additional weight on studies from under-represented biomes, regions and taxonomic groups. However, although the dataset is unprecedentedly large and diverse, given the total global diversity of biomes and taxa it is not large or diverse enough to support such a balancing. Hopefully in time this may be possible, but this would seem a long way off. For now we have to make do with what we have, while making clear where the limitations of our data are.

*Finally, why are 2-tailed tests used? You have a clear expectation of larger body sizes declining more.*

Yes, we had a literature-based expectation that larger body-sizes would decline more (a negative mean $\tau$). However, this was not the only option, making a 2-tailed test appropriate (as indeed turned out to be the case with the positive mean response in many of the traits.) To change the orientation of the tests now (knowing the results) would not be statistically valid. If a one-tailed (negative tau only) test was carried out, it would not 'reveal' a negative trend (all calculated two-tailed p-values for negative mean taus were over 0.1).

*Minor points:*

*The authors binned widespread species into 10km grid cells. I wonder if this causes issues of non-independence.*

This would be a potential concern with certain analysis approaches. However, we used as our principal unit of replication the average response across the assemblages th**at make up the whole 'study'. Therefore, if similar** (non-independent) dynamics were occurring with two adjacent grid cells, then this would still only contribute a single point in the final collection of studies. We do not therefore believe that non-independence is a particular concern as our analysis approach is quite conservative.

We followed previous analyses using the BioTIME database to generate localised time series from widely distributed observations (as cited in the manuscript). If this spatial deconstruction was not carried out, differences through time of where samples were obtained (for example, due to changes in the routes of research cruises) could introduce artefacts. To some extent the choice of grid cell size was arbitrary but was selected as being in line with the spatial scale of the localised studies.
*The authors attribute the positive relation for amniote studies to anthropogenic dispersal limitations (affects smaller bodies animals more). Could the authors explain this more? Is anthropogenic dispersal limitations increasing during the time periods of the studies?*

We have slightly expanded the text on this topic [lines **79-82] . We don't wish to put too much weight on this** possible explanation - we were just citing a putative explanation for positive size trends from existing literature. Although increasing anthropogenic fragmentation (and hence dispersal limitations) is reasonable, we do not have any specific data to support that explanation over others.

Reviewer #2:

***The manuscript: "No pervasive size trend in global community dynamics" investigates whether there is a global*** *trend of decline in species body size. For that, the relationship between size traits and changes in abundance rank over time is analysed for several assemblages worldwide.*

*The study is well designed and make use of the BioTime database and several trait databases. The text is concise and well written and the figures and supporting materials are informative. The authors did a good job discussing the caveats of using such databases and performing such large-scale analyses. The conclusions are supported by the results and, in my opinion, the manuscript is of potential interest to the readers of this journal.*

Our thanks to the reviewer for the positive and supportive comments.
*I appreciate that the authors explicitly mention that insects are not included in the analysis. I recommend to also discuss bias in the distribution of data across realms: are freshwater assemblages included in the study? This is relevant to the scope of the manuscript, as freshwater megafauna is reported as declining globally, see e.g. He, F., Zarfl, C., Bremerich, V., David, J. N., Hogan, Z., Kalinkat, G., ... & Jähnig, S. C. (2019). The global decline of freshwater megafauna. Global change biology, 25(11), 3883-3892.*

At a very coarse level, the different traits largely divide up the terrestrial and aquatic realms (although not perfectly, and extremely coarsely). In terms of freshwater systems, this is an insightful point. There were 3 freshwater fish studies with sufficient data to be used in our study (out of 28 freshwater realm studies in our version of the BioTIME database, although that covers fish, plankton, gastropods, turtles, aquatic plants, amphibians and insects). Clearly this is not enough to construct a meaningful separate analysis. We now split the maps of studies into separate aquatic and terrestrial realms, and explicitly note the number of freshwater studies that we have the data to include.

There are a large number of biases within BioTIME, and in the interest of space we must be selective about the points we discuss. An extensive discussion of the biases within the database has previously been published:

[redacted]

FIGURE 2 Proportion of studies that fall into the different classifications of: Climate, number of years sampled, realm, taxa and biome

From: Dornelas et al (2018). BioTIME: A database of biodiversity time series for the Anthropocene. *Global Ecology and Biogeography*, *27*(7), 760–786. https://doi.org/10.1111/geb.12729 (CC-BY)

We have added a point about the biases in biogeographic realms in general in our discussion of biases (since the issue is larger than just freshwater systems), but we believe the lack of insect data in our dataset to be the most concerning and in need of highlighting [lines 109-121].

We have also added the He et al citation to our discussion of how our results are community analysis, not especially directed to the plight of apex species.
*From Extended Data Figure 1, it seems that the majority of time series are less than 20-years long. It could be that the decline or extinction of large-bodied species happened before the beginning of the time series. In that case, the time series cannot track the changes in size:ranks relationships. I recommend to discuss this issue.*

Indeed, this is a potentially significant issue, and we have extended our discussion of this issue in our manuscript [Lines 86-94]. The issue of preceding declines is problematic and intrinsically hard to address. That said, we do not see a consistent relationship between the duration of the time series and our tau. We now highlight in the manuscript that the duration of the fish studies is negatively correlated with tau – i.e. positive tau values seem to be linked to short study durations, which may indicate recoveries from past pressures (Extended Data Table 1). However, since very long time series form only a small part of BioTIME, this is only very weakly testable with the data we have to hand. In the plants, the effect of study-duration is in the other direction, for which we do not have a simple explanation to offer.

*Here some additional comments:*

*Line 16: delete "is".*

Done – thanks for the spot

*Figure 1b: The symbols are too small and difficult to distinguish. Please, increase their size to improve readability.*

We have been able to increase the size a small amount, and now split the maps into realms (reducing the number of points on each map). The shape of the points conveys a relatively less important piece of information than the colour and the location spread of the points.

***Line 119: The caption of figure 2 says that "Blue density plots show the distribution of 50 randomisations". I*** *wonder why 50 randomizations and not 100, as 100 were used to create the null distribution according to lines 47 and 185.*

This was a mistake on our part, failing to update the caption text during revisions. As discussed in our response to reviewer 1, we now take a different approach to statistically summarising our overall results.

***Line 177, "very small β values": Can you please define what "very small" means?***

Apologies for this oversight. We have added the cutoff (< 0.00001) we used to the method section. However, in practice most of these are numerical rounding errors that were very much smaller than this (of the order $10^{-14}$).

***Line 191: delete "that".***

**Done – thanks for the spot**

*Lines 189-194: From Extended Data Figure 1, it seems that there are a few outliers in the raw data and skewed distributions. Were the explanatory variables transformed?*

Yes. Where appropriate, predictor data was log-transformed and these transformations are stated explicitly in the methods section and in Extended data Figure 2, tables 2 and 3.

Reviewer #3:

*This study assesses whether temporal species turnover in community time series results in functional changes related to species size. This is an interesting and relevant question because numerous studies have pointed out size-related vulnerability to anthropogenic threats, and other have concluded that communities will be increasingly dominated by small species. I think this can be an important contribution with potentially interesting results. However, overall I felt a bit dissatisfied with the study. My main concerns relate to the analyses and hypotheses.*

We are glad to see that the reviewer sees value in the aims of the study. We hope that our responses below can assuage their concerns. In fact, we share many of the concerns and are at pains to make clear in the manuscript exactly what the results can and cannot tell about the state of global communities.

***1) I don't think using ranks is a good idea. Using ranks makes the analysis quite insensitive to changes, and*** *does not focus on changes per se but rather to reversals in species relative abundances. A species can remain stable and another may be declining, but in a matter of 15 years or so the rank will likely remain stable. Take a simplistic example of a community with two species, a mouse and a tiger. If the tiger decreases or increases* ***while the mouse remain stable you won't see any change in the ranks, as the tiger will always be rarer than the*** *mouse. Similarly, if the mouse decreases and the tiger increases, the rank will also remain invariant as these two species differ by orders of magnitude in their population density.*

*So at present, I'm not convinced by the conclusions made by the authors.*

As previously discussed, we now present a non-rank based method in our main text results. However, we maintain that the rank-based approach can be informative. Although the concerns set out by the reviewer are a possible threat in principle, in practice there are many rank changes in the dataset. Ranking, and changes in ranks, is a relatively simple tool to standardise the measurement of ecological communities, but that does not necessarily mean that it is insensitive. Rank analyses can be informative at analysing global change (e.g. two

recent high-profile examples, Avolio et al (2021), Jones et al (2017)). For this dataset in particular, rank changes are more robust to irregular in sampling frequency and intensity through time, something that is otherwise very hard to control for in the BioTIME database.

A large proportion of these communities **do in fact show a considerable amount of turnover – indeed this was** a feature of many of the previous analyses using the BioTIME database. Most species change their rank between most sampling points. The communities have many more species than the two in the example the reviewer puts forward and rather than comparisons between completely different species (mice and tigers), **most studies focus on a particular guild – lizards in on**e area, songbirds somewhere else, etc. This means that **in practice (to continue the reviewer's example) that wh**ile the mice may not change ranks with the tigers of a particular study, they *could* and do change rank with the large number of other rodent species that would be included.

**This does leave the equivalents of 'tigers' without a di**rect comparison, as a rare large top predator. However, such pinnacle species (almost by definition) form a very small proportion of the overall community. We made this point in the original manuscript, when we set out how our analyses do not in any way detract from the plight of large apex species. We are taking a community ecology angle, not a conservation one.

That said, we do not want the potential issues around the sensitivity of rank analyses to be a distraction from our results, and it is clear that the rank-approach was causing unease. Using an alternative approach in our main text (that gives very similar results) and moving our rank-based metric to supplementary material seems to be the best option.

*I see two possible options. One would consist in a linear model with abundance as dependent variable and body mass and time as predictors. The interaction between the two should inform on size-related changes. In such a model you could also include a number of potentially confounding variables (e.g. those assessed in Fig. 1 in extended data), which would make results more robust to these factors than a simple visual inspection. Obviously this requires some thinking about random intercepts/slopes etc..*

We actually shared the initial instinct to build a single (enormous) hierarchical model along the lines that the reviewer suggests. It would be technically possible to build such a model in, for example, STAN. However, in practice the approach would introduce considerable additional problems and rely on assumptions that could not be well justified (as we discussed previously in our response to the comments of R1).

There are several fundamental constraints with the datase**t – the trait data is incomplete, the total amount of** sampling at each time point within a series is not necessarily consistent, and the number and total time span of the sampling varies between assemblages. All of these factors conspire to make the hierarchical model approach difficult to justify, because the implicit weighting assigned within and between each level in the data structure (observation / species / assemblage / study) would become obscured. Although the compartmentalised approach we took cannot propagate uncertainties up to the final result, it is not clear to us that there would be additional light shed from this process.

What we can do is test different methods to defining how species populations are changing through time. As mentioned above, we now include two additional (non- abundance rank based) measures of population change. These make slightly different assumptions, but turn out to be highly correlated with each other and the original rank-based approach.

The first method we use (and now present in the main text) is based on the approach of Dornelas et al (2019) (hereafter D19). This takes the raw data and cuts off strings of zeros at either end of the time series to avoid shrinkage of fits towards a flat line. In a slight divergence from D19, we retained a single zero on either side of the main trend to better capture colonisations or extinctions (D19 stated they removed all leading or trailing zeros, but as their code is not linked to the paper, we **can't check). The raw values are then transformed by** taking the square root. This stabilises the variance between populations and can handle zeros well compared

to taking a logarithm. Then each population is scaled (by subtraction) to a mean of 0 and (by division) to a standard deviation of 1. Through this we fitted an OLS-regression against the year, to extract a trend.

In a third approach, we maintained the removal of excess trailing or leading zeros, but transformed instead by dividing each time series by its mean. An OLS-fit through data transformed in this way captures another aspect of the change through time of the population.

Confirming the robustness of our results, there were strong correlations at the species level between the slopes calculated in both of these approaches and the original rank-based metric. Similarly, the overall results were very similar between the three approaches. We include these results in Extended Data Figure 4 and 5. Given this consistency (and the fact that this manuscript **is a 'brief communication') we present just a single** metric in the main text. We chose the approach of D19 as it is already established in the literature and as such will hopefully elicit less unease than our original rank-based approach.

Further, by putting this approach to the forefront it also **allows us to concisely provide an additional 'species-level' analysis that takes into account the confidence wi**th which the dynamics of each species can be assigned (Extended Data Figure 3). In this supplementary analysis we continue to follow the overall method of D19, we **label each species in each assemblage as a 'winner', 'loser' or having no detectable trend based on the sign** and significance of the regression line. Then we can test how the relative trait rank of a species within a community influences the likelihood of being a winner or loser (weighting down cases where a species was observed in multiple assemblages in the same study). This related analysis therefore has a different approach to emphasising aspects of the available data, but also shows no overall trend, increasing further the robustness of our overall message.

Finally in response to this comment, we must emphasise **that we did more than a 'simple visual inspection' of** the effect of the covariates. Although we do discuss the plots qualitatively we also fitted and test models (Extended data tables 1 and 2)

*A second option I can think of, is focusing on some community weighted average of body size and how this changes over time. You can still analyse this using a linear model.*

We agree that this is an appealing approach, and this would in many ways be the ideal option if the trait data was complete. However, with the incomplete nature of the trait data, it would be necessary to make strong assumptions about the distribution of missing trait data. It would also require strong choices about the transformation of size traits, introducing additional complication. We believe that in this case, such an approach would be unsafe and unjustifiable.

*2) The authors start by explaining that studies focusing on time-series have observed species turnover, and others have claimed different trends for species with different sizes. This is true, however, most of the literature on this (at least the one I know) relates to endothermic vertebrates, and here the authors analyse several different taxa including plants.*

While there is certainly a lot of data in the conservation literature around vertebrates, the largest studies that take a community ecology perspective area have come from the broader BioTime database. Within this dataset endothermic vertebrates are a large portion, but by no means dominant (see middle right panel in the figure we included in our response to R2). Earlier work on this topic that we cite (Vellend et al 2013) was entirely plant based.

*I'd like to see more space dedicated to what is known or not known, how this knowledge is taxonomically biased, and so what can we expect from these results. Also note that even within taxa, authors have pointed out different size-trends (e.g. Ripple et al. 2017 PNAS, or Santini et al. 2021 Ecol Lett cited in the text). Currently the paper jumps into the methods and results too soon, I think the authors need to dig deeper in the literature*

*and provide an overview of what is known and could be expected.*

We have been happy to expand our introductory paragraphs with more text and references [e.g. lines 16 - 19] but are inevitably somewhat constrained by length limits of a 'brief communication' (1500 words).

There are multiple potential drivers of body-size shifts that need to be summarised and unfortunately there is not a single definitive literature source to ground expectations in. For example, we note that Merckx et al (Nature 2018 *Body-size shifts in aquatic and terrestrial urban communities*) state in their first paragraph 'global climatic warming is expected to drive shifts to communities consisting of smaller species' but cite only Scheffers et al (2016), who do not make this prediction – the paper only directly discusses within-species body-size shifts, something that neither we or Merckx et al can detect with our available data. Although there has been considerably more research on within-species trait changes in response to climate, we think it is clearer to discuss this aspect a later in the manuscript (and have added an extra citation to a recently released paper: Hantak et al 2021).

We believe it is important to make a distinction between global species level vulnerability and trends within local communities (that we focus on). The cited Ripple et al paper (and others in that tradition) focus on global population extinction risks - while there is clearly some overlap, the processes have different contributing factors. The Santini & Issac paper is closer to our question of interest, but takes a quite different approach (for example using global population estimates and comparing populations that do not coexist, rather than localised communities). We cite both, because they provide crucial benchmarks, but are not completely comparable.

The underlying expectation of climate effects are fairly straightforward – if we observe that bodysizes with guilds are related to temperature (Bergmann's rule), then it is reasonable to expect the distribution of body-sizes in the community to follow temperature changes. We have expanded our description of this line of reasoning. We also now include a gateway reference into the literature attempting to use metabolic theory to predict community responses. Ultimately however, trends in our results will reflect a mixture of different pressures on communities, making a single clear expectation unreasonable.

*11-12: This comes a bit out of the blue in the abstract, both because specifically focused on body size (but it starts refering to traits in general), and because of the expectation small and large may do better/worse. Perhaps an introductory sentence on common perception/previous studies is worth adding on this*

We do agree that it is rather rapid, but unfortunately we are constrained to 100 words and 3 sentences in our abstract. However, we would not agree that our final abstract sentence presupposes a particular direction of trend (it would equally apply if 'larger' and 'smaller' were swapped).

*L. 19-20: See also*
*González-Suárez, M., Gómez, A., & Revilla, E. (2013). Which intrinsic traits predict vulnerability to extinction depends on the actual threatening processes. Ecosphere, 4(6), 1-16.*
*For how threats-vulnerability is related to spefici traits in mammals*

Our thanks for the reference – we have included it at the point where we discuss out that body-size – extinction risk patterns are threat dependent

*Ripple, W. J., Wolf, C., Newsome, T. M., Hoffmann, M., Wirsing, A. J., & McCauley, D. J. (2017). Extinction risk is most acute for the world's largest and smallest vertebrates. Proceedings of the National Academy of Sciences, 114(40), 10678-10683.*

*For how vertebrates body mass relates to extinction risk (note that here they conclude both small and large are at higher risk for different reasons).*

Yes, we included this reference and highlighted the context-dependency.

*Cooke, R. S., Eigenbrod, F., & Bates, A. E. (2019). Projected losses of global mammal and bird ecological strategies. Nature communications, 10(1), 1-8.*

*Here the authors projected community changes into the future for birds and mammals*

This is certainly an interesting paper, but we feel does not add additional information to the other references for our purposes.

*In general, you here are assessing entire communities, but as far as I know the literature on body-size related trends is mostly on mammals, and secondarily on vertebrates. The expectation for entire communities or different taxa needs to be better grounded in the literature*

Indeed, our approach is fairly distinct to the large body of literature relating traits to extinction risk. A vertebrate focus is certainly true for the assessment of extinction risks. However, in terms of trends within communities linked to climate change, the expectations are perhaps best established within aquatic systems, e.g.:

- Yvon-Durocher et al Warming alters the size spectrum and shifts the distribution of biomass in freshwater ecosystems. *Global Change Biology* **17, 1681–1694 (2011).**
- Daufresne et al . Global warming benefits the small in aquatic ecosystems. *Proceedings of the National Academy of Sciences of the United States of America* **106, 12788–12793 (2009).**
- Morán et al (2010). Increasing importance of small phytoplankton in a warmer ocean. Global Change **Biology, 16(3), 1137–1144. https://doi.org/10.1111/j.1365-2486.2009.01960.x**
- **O'Gorman et al (2017). Unexpected changes in** community size structure in a natural warming **experiment. Nature Climate Change, 7(9), 659–663. https://doi.org/10.1038/nclimate3368**

Our additions to the introduction (as discussed above) should clarify these issues.

*Line 28: is there any evidence that larger/smaller plants do better/worse?*
*I only remember this paper*
*de Jonge, M. M., Hilbers, J. P., Jongejans, E., Ozinga, W. A., Hendriks, A. J., & Huijbregts, M. A. (2018). Relating plant height to demographic rates and extinction vulnerability. Biological Conservation, 220, 104-111.*

Thank you for the reference. We have added it in to our discussion of the plant traits results alongside an earlier paper relating plant size to life-history (Marba et al 2007). The use of size-traits in plants is more speculative that for animals (as we discuss in the manuscript), but we think is worth including.

To better ground the plant work, we now cite a tundra study that showed increases in community plant height (driven by species turnover) that was linked to climate warming (Bjorkman et al 2018). We considered citing Wieczynski et al 2019, but decided against it as that study uses only the distribution of traits at a particular point in time, not changes through time, which we thought would take too much space to differentiate.
***Line 50: positive relationship between body mass and "positive/negative"(?) trends?***

We have heavily rephased this part [Lines 54-66]

***Line 53: "predict" is a big word. I can imagine they do not predict them, but did they influence them? Did you*** *find any relationship even if weak?*
*It would really help to add a loess fit on top of the scatterplots for better interpretation (but see my comment above on the need of a more robust way to account for these factors on the results).*

**Predict, or 'to be predictive of' would seem to us a pr**etty standard way of describing a directional statistical **relationship without implying a direct mechanistic link that 'influence' would imply.**

All results from our tests are detailed in Extended data tables 1 and 2. They show that the full set of explanatory variables largely had extremely weak relationships with the tau values. As we discuss in the manuscript, although certain factors were identified as significant for certain traits (without correcting for multiple comparisons), this was inconsistent - suggesting that it is not a driver that would cause notable artefacts. As we discussed above, building an all-encompassing model would create significantly more problems than it would solve.

Because of this lack of support for a relationship, we do not introduce model fit lines in order to avoid implying a relationship when there is no evidence for one. In this case LOESS fit lines also lead to overfitting and do not aid interpretation:

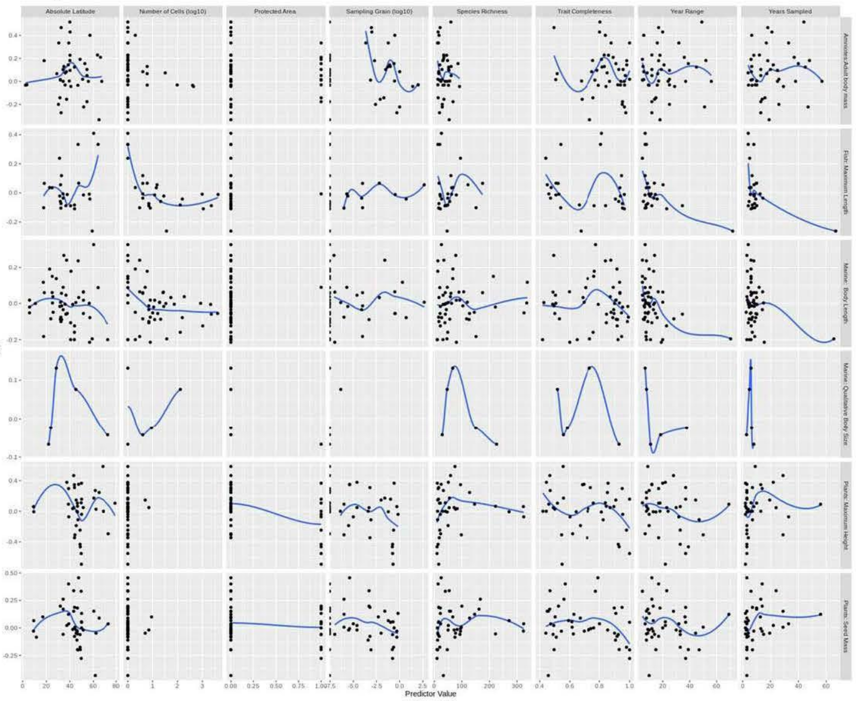


*Also, it seems to me that some variables may benefit from transformation, e.g. species richness*

As we detail in our methods, we transformed the most heavily skewed parameters (number of cells and spatial grain of the original study). Inspection of the model diagnostic plots showed that there was no need to transform species richness.

*Line 62-63: Is the direction of these changes consistent? Or no consistent pattern is observed?*

**There was no consistent pattern observed – hence the la**ck of an overall clear trend. We have added some reiteration of this point to this sentence.

*65-67: Density is strongly negatively related to body mass, so habitat area is a bigger issue for large than small species*

Indeed. We have added a mention of habitat loss alongside habitat fragmentation to our expanded introduction [lines 22-23].

*Line 70-71: Sometime the description/interpretation of the results is hard to follow, here and elsewhere I suggest explaining the pattern in words rather than referring to t and its statistical distribution.*

We have made several edits to reduce distribution-focussed results description.

***90: This should be "meta-analyses", not "metanalyses" .***
*Note, however, that most of these analyses are not proper meta-analyses in sensu strictu, just statistical analyses*

**True - we have changed to just 'analyses' here.**

***Fig. 1 What are the coloured lines in b? It is a bit confusing, I'd only show the points. Also, include symbol*** *legend in the figure for simplicity*

We now split the sites by realm, to make the figure easier to parse as there are fewer points per map. The lines are label lines that show the location of studies that would otherwise overlap and be impossible to distinguish if only the points were shown. We have also added an inset legend for the different traits rather than listing the shapes in the caption.

*139: Honestly, I would not trust the GBIF backbone taxonomy, gbif is full of misspelled names, synonyms, subspecies treated or not treated as species, etc.*
*Why not using catalogue of life in taxize?*

While we share the reviewers caution, the choice of GBIF was to try and cross-reference as many names between the trait databases and BioTIME as possible (it is worth noting that GBIF starts with CoL and then expands). Our approach to using the backbone taxonomy was quite conservative, rejecting records labelled as synonyms etc wherever possible.

*Fig. 2 It may help to add % >0 for better interpretation*

This is possibly helpful, but we fear might lead to an expectation that our final statistical test was non-parametric test based on the frequency of signs. With our switch to a slightly different final analysis, this figure is now different in any case.

*Line 218: I miss a rationale for it. Why do we need a null model? Why should we expect null model to deviate from no change?*

We have discussed this in depth in our response to R1, but for completeness the purpose of the null model is principally to determine a baseline expectation for the variance around 0 if there was no influence of traits, in order to assess the significance of deviations in the observed distribution of tau values. Under the null hypothesis the mean would not be expected to differ from zero (and does not).

*One final point to take in consideration. The time series are, in most cases, quite short to detect such a trend. We may be able to detect changes in species with rapid life histories, but are we capable of detecting changes in species with slow life histories (the large ones)? This is something that is worth discussing and perhaps take into account in the analysis if possible*

As discussed in our response to R2, we have introduced additional discussion about the possible influence of study-span in our revision.

We included time span of the study as one of the suite of predictor variables we examined. It did not have a notable or consistent impact. However, as the reviewer points out, the best measure would be some count of the number of generations covered by the study. Unfortunately, this would not be straightforward to gather the data for.

In terms of the reviewers later points, at a within-study level, if large species were fixed but smaller species were changing around them, that is exactly the type of signal we would be looking for - it would not represent a problem as such.

**References cited:**


Antão, L.H., Bates, A.E., Blowes, S.A., Waldock, C., Supp, S.R., Magurran, A.E., et al. (2020). Temperature-related biodiversity change across temperate marine and terrestrial systems. Nature Ecology and Evolution, 4, 927–933.

Avolio, M. L., Komatsu, K. J., Collins, S. L., Grman, E., Koerner, S. E., Tredennick, A. T., … Tognetti, P. M. (2021). Determinants of community compositional change are equally affected by global change. *Ecology Letters*, (April), ele.13824. https://doi.org/10.1111/ele.13824

Bjorkman, A. D., Myers-Smith, I. H., Elmendorf, S. C., Normand, S., Rüger, N., Beck, P. S. A., … Weiher, E. (2018). Plant functional trait change across a warming tundra biome. *Nature*, *562*(7725), 57–62. https://doi.org/10.1038/s41586-018-0563-7

Collins, S. L., Suding, K. N., Cleland, E. E., Batty, M., Pennings, S. C., Gross, K. L., … Clark, C. M. (2008). Rank clocks and plant community dynamics. *Ecology*, *89*(12), 3534–3541. https://doi.org/10.1890/07-1646.1

Dornelas, M., Gotelli, N. J., Shimadzu, H., Moyes, F., Magurran, A. E., & McGill, B. J. (2019). A balance of winners and losers in the Anthropocene. *Ecology Letters*, *22*(5), 847–854. https://doi.org/10.1111/ele.13242

Jones, S. K., Ripplinger, J., & Collins, S. L. (2017). Species reordering, not changes in richness, drives long-term dynamics in grassland communities. *Ecology Letters*, *20*(12), 1556–1565. https://doi.org/10.1111/ele.12864

Marbà, N., Duarte, C. M., & Agusti, S. (2007). Allometric scaling of plant life history. *Proceedings of the National Academy of Sciences of the United States of America*, *104*(40), 15777–15780. https://doi.org/10.1073/pnas.0703476104

Merckx, T., Souffreau, C., Kaiser, A., Baardsen, L. F., Backeljau, T., Bonte, D., … Van Dyck, H. (2018). Body-size shifts in aquatic and terrestrial urban communities. *Nature*, *558*(7708), 113–116. https://doi.org/10.1038/s41586-018-0140-0

Scheffers, B. R., De Meester, L., Bridge, T. C. L., Hoffmann, A. A., Pandolfi, J. M., Corlett, R. T., … Watson, J. E. M. (2016). The broad footprint of climate change from genes to biomes to people. *Science*, *354*(6313). https://doi.org/10.1126/science.aaf7671

Vellend, M., Baeten, L., Myers-Smith, I. H., Elmendorf, S. C., Beauséjour, R., Brown, C. D., … Wipf, S. (2013). Global meta-analysis reveals no net change in local-scale plant biodiversity over time. *Proceedings of the National Academy of Sciences*, *110*(48), 19456–19459. https://doi.org/10.1073/pnas.1312779110

Wieczynski, D. J., Boyle, B., Buzzard, V., Duran, S. M., Henderson, A. N., Hulshof, C. M., … Savage, V. M. (2019). Climate shapes and shifts functional biodiversity in forests worldwide. *Proceedings of the National Academy of Sciences*, *116*(2), 587–592. https://doi.org/10.1073/pnas.1813723116

White, E. P., Ernest, S. K. M., Kerkhoff, A. J., & Enquist, B. J. (2007). Relationships between body size and abundance in ecology. *Trends in Ecology and Evolution*, *22*(6), 323–330. https://doi.org/10.1016/j.tree.2007.03.007

**Decision Letter, first revision:**

24th September 2021

*Please ensure you delete the link to your author homepage in this e-mail if you wish to forward it to your co-authors.

Dear Dr Terry,

Your manuscript entitled "No pervasive size trend in global community dynamics." has now been seen again by the three reviewers, whose comments are attached. You will see that while Reviewers 1 and 2 now endorse publication, Reviewer 3 continues to raise some concerns about the analyses. We would like to see this comment addressed before we can reach a final decision regarding publication. Although the reviewer does not mention this, we wondered if analyses of simulated data could be used to explore the contingency of the results on the range of body sizes in the data.

We therefore invite you to revise your manuscript taking into account all reviewer and editor comments. Please highlight all changes in the manuscript text file.

We are committed to providing a fair and constructive peer-review process. Do not hesitate to contact us if there are specific requests from the reviewers that you believe are technically impossible or unlikely to yield a meaningful outcome.

When revising your manuscript:

* Include a “Response to reviewers” document detailing, point-by-point, how you addressed each reviewer comment. If no action was taken to address a point, you must provide a compelling argument. This response will be sent back to the reviewers along with the revised manuscript.

* If you have not done so already please begin to revise your manuscript so that it conforms to our Brief Communication format instructions at http://www.nature.com/natecolevol/info/final-submission. Refer also to any guidelines provided in this letter.

* Include a revised version of any required reporting checklist. It will be available to referees (and, potentially, statisticians) to aid in their evaluation if the manuscript goes back for peer review. A revised checklist is essential for re-review of the paper.

Please use the link below to submit your revised manuscript and related files:

***[REDACTED]***

<strong>Note:</strong> This URL links to your confidential home page and associated information about manuscripts you may have submitted, or that you are reviewing for us. If you wish to forward this email to co-authors, please delete the link to your homepage.

We hope to receive your revised manuscript within four to eight weeks. If you cannot send it within

this time, please let us know. We will be happy to consider your revision so long as nothing similar has been accepted for publication at Nature Ecology & Evolution or published elsewhere.

Nature Ecology & Evolution is committed to improving transparency in authorship. As part of our efforts in this direction, we are now requesting that all authors identified as 'corresponding author' on published papers create and link their Open Researcher and Contributor Identifier (ORCID) with their account on the Manuscript Tracking System (MTS), prior to acceptance. ORCID helps the scientific community achieve unambiguous attribution of all scholarly contributions. You can create and link your ORCID from the home page of the MTS by clicking on 'Modify my Springer Nature account'. For more information please visit please visit <a href="http://www.springernature.com/orcid">www.springernature.com/orcid</a>.

Please do not hesitate to contact me if you have any questions or would like to discuss these revisions further.

We look forward to seeing the revised manuscript and thank you for the opportunity to review your work.

***[REDACTED]***

Reviewers' comments:

Reviewer #1 (Remarks to the Author):

The authors have adequately addressed my concerns, and I believe that the interpretation of the results is sound (and is interesting). I agree with their presentation of multiple metrics of change (including keeping the ranked version in the extended material), and that the results are robust to these choices. I also agree with the secondary, species-based analysis. Finally, although this was not addressing one of my explicit comments, I liked that they kept their interpretation of significance of the amniotes more cautious and did not over interpret it. Overall, I think the authors have done a good job.

Reviewer #2 (Remarks to the Author):

The authors did a good job in reviewing the manuscript. The new analytical approaches make the manuscript stronger. I have only a few minor comments.
The reference to Figure 2 precedes that of Figure 1 (line 52 vs line 107).
Line 71, "overall positive association": Please, specify what association this is.
Line 107: Is the reference to Figure 1b correct, or should it refer to the whole Figure 1?
Line 173, "Data not from adults was discarded": this is stated also at line 170.

Reviewer #3 (Remarks to the Author):

In the first revision, me, and the other two referees had similar concerns on the sensitivity of the rank analysis to changes. The authors have argued that even if this is true in principle, this is unlikely in practice and that the conclusions do not change when using different approaches.
That other approaches lead to similar results is re-assuring, but in a way it is potentially concerning. I made a simplistic and extreme example in my previous comment (tigers and mice), and authors argued that that was indeed extreme and there's no similar case in their dataset, as most time series focus on particular guilds such as lizards or song birds. I find this very concerning, as the results are likely heavily dependent on the variability of taxa included in each time series. I expect that size-related trends are only visible if the community data includes very large and very small species, but not if the data only include similar-sized species. Is there any relationship between size-trait range (or better, 95% of the distribution) with the trend observed?

Please note that I revised the pdf with track-changes, so the line numbers below correspond to that pdf document.

One general comment: The author often refer to "traits" in general, e.g. "relative trait rank within an assemblage". However, as all traits are size traits, I suggest repeating "size traits" all the times to avoid confusion.

Also, the authors indeed argued that their methodological options are limited due to gaps in the data, however I am still confused on how authors dealt with incomplete data, and would like to see this better explained in the text.

L 54: several?

Line 62: See my comment above on the potential influence of size range in the community values.

L. 68-69: The authors should also discuss how representative the biotime is taxonomically. Most of the time series do not include large vertebrate species, which are those on which much of this size-dependent threat literature has been developed

l. 96 I think "spatial, temporal and TAXONOMIC BIAS" would be more appropriate than "limitations"

L 110: I think this is the first time you mention 3 approaches. Above you only say you repeat the analysis using different transformations and cite the SI.

L. 224-226: cite the corresponding SI here.

L. 248: I am not sure I understand this. At least 5 times the species occur in the time series? i.e. observed at 5 time steps?
Please clarify. Also, as this is based on a continuous value, why not using that value directly instead of binarizing into winner and looser which degrades the information into a threshold-dependent binary outcome?

Extended Data Table 1: Revise sentence "There was no consistent drivers.."
I am not sure how to interpret the table. What I find confusing is the response variable. From the text (l. 62) is seems like you use the correlation coefficient, but here it seems you use a trait variable? Please clarify.

Extended data Table 4: I find hard to understand this: “relationship between the relative trait rank within an assemblage and the assignment to either ‘winner’ or ‘loser’ categories”. Please clarify. In general, I think it would be really useful if you could elaborate more on some part of the methods in a dedicated section of the SI, especially the different approaches to assess trends.

I would only provide Extended Data Table 6 as a csv table, not also in the pdf

*******************END*******************

**Author Rebuttal, first revision:**

**Reviewer #1 (Remarks to the Author):**

*The authors have adequately addressed my concerns, and I believe that the interpretation of the results is sound (and is interesting). I agree with their presentation of multiple metrics of change (including keeping the ranked version in the extended material), and that the results are robust to these choices. I also agree with the secondary, species-based analysis. Finally, although this was not addressing one of my explicit comments, I liked that they kept their interpretation of significance of the amniotes more cautious and did not over interpret it. Overall, I think the authors have done a good job.*

Our thanks to the reviewer for the supportive comments. We very much agree that a cautious interpretation of the results is essential.

**Reviewer #2 (Remarks to the Author):**

*The authors did a good job in reviewing the manuscript. The new analytical approaches make the manuscript stronger. I have only a few minor comments.*

Our thanks to the reviewer for the positive and constructive comments.

*The reference to Figure 2 precedes that of Figure 1 (line 52 vs line 107).*

Thank you for the spot. We think it is makes marginally more sense for the maps to come before the main results plot (although both are to some extent ‘results’). We have introduced an earlier reference to figure 1 to keep the ordering sensible. (Line 37)

*Line 71, ”overall positive association”: Please, specify what association this is.*

We have added additional clarification (L.75)

*Line 107: Is the reference to Figure 1b correct, or should it refer to the whole Figure 1?*

Indeed - we have adjusted. This was a relic of our previous draft.

*Line 173, ”Data not from adults was discarded”: this is stated also at line 170.*

This is true; however, the first reference refers to quantitative body size measurements, while the second refers to the qualitative traits. While we could probably find a way to phrase it once to cover both categories, it would likely be longer and more convoluted than our current formulation

**Reviewer #3 (Remarks to the Author):**

*In the first revision, me, and the other two referees had similar concerns on the sensitivity of the rank analysis to changes. The authors have argued that even if this is true in principle, this is unlikely in practice and that the conclusions do not change when using different approaches.*

*That other approaches lead to similar results is re-assuring, but in a way it is potentially concerning. I made a simplistic and extreme example in my previous comment (tigers and mice), and authors argued that that was indeed extreme and there's no similar case in their dataset, as most time series focus on particular guilds such as lizards or song birds. I find this very concerning, as the results are likely heavily dependent on the variability of taxa included in each time series. I expect that size-related trends are only visible if the community data includes very large and very small species, but not if the data only include similar-sized species. Is there any relationship between size-trait range (or better, 95% of the distribution) with the trend observed?*

This is an interesting point, distinct to the points discussed around the previous version of the manuscript. Like all cross-study comparative analyses, there is a balance to be struck between comparing like-with-like and also having a sufficient range to detect signals through noise. To continue the analogy further, a comparison of a restricted set of mice might span a very small size range that would be hard to relate to trends. On the flip-side, comparing tigers with mice is also likely to be heavily influenced by other drivers. Most ideally for the questions we want to address, a scale roughly of 'rodents' would probably hit the sweet spot. To some extent we do have to trust that the original compilers of the data contained in BioTIME made reasonable choices of the scale of data, that they do indeed represent meaningful communities. The BioTIME dataset is specifically curated for the analysis of communities, and has been subject to much discussion and analysis, so we believe this is a reasonable assumption.

To address the possibility that trait-ranges have a driving influence on our results, we have looked into the data and do not find any particular cause for concern and have incorporated the results into the manuscript.

For this analysis, for each assemblage we took the difference between the log10(minimum) and log10(maximum) values of traits for the species that were actually used in the analyses (i.e. excluding very transient species that were filtered out as described in the main text). Clearly there is a very large number of ways to describe the spread of traits in each community, but this seemed the most direct. We looked at how this relates to our tau metric (for multi-site studies we took an average trait range, so each dot represents a single study-trait combination).

Contrary to the reviewer's expectation, there is no obvious problematic trend in the divergences from zero and the trait range. Most assemblages showed ranges of about 1-2, (i.e. a 10-fold to 100-fold size

range) Note that some traits are lengths and some are masses - lengths showed a smaller range (as would be expected based on the number of dimensions involved).

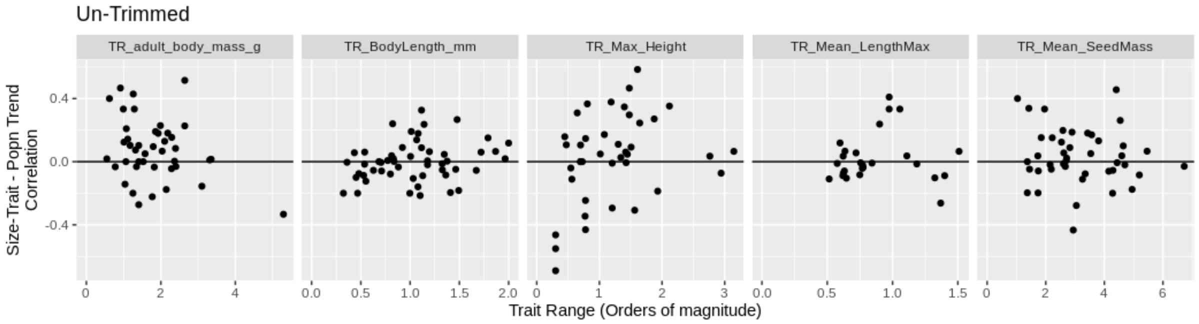


We repeated the analysis excluding the 5% largest and 5% smallest species, and the results were very similar:

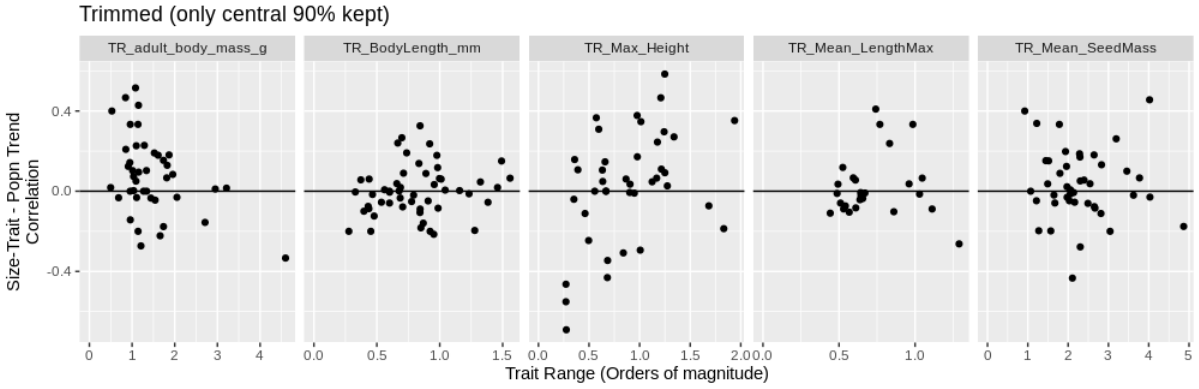


To address this in the manuscript, we have added this metric of ‘trait range’ as an additional predictor into SI Fig2, alongside the other potential factors such as time-series length and number of species (L61, L221). We have split the figure into two parts because the sub-facets were getting too small. Because the results were so similar, we only included the ‘untrimmed’ trait range.

We have added a sentence to the main text making our assumptions on this explicit (L95-96). Additionally, we have added some extra explanation about what a BioTIME ‘study’ constitutes (L28), and added summary statistics of the trait range to the main text (L42-43).

*Please note that I revised the pdf with track-changes, so the line numbers below correspond to that pdf document.*

*One general comment: The author often refer to “traits” in general, e.g. “relative trait rank within an assemblage“. However, as all traits are size traits, I suggest repeating “size traits” all the times to avoid confusion.*

This is a good idea – we have gone through the manuscript and clarified this in a number of places where we present our methods and results.

*Also, the authors indeed argued that their methodological options are limited due to gaps in the data, however I am still confused on how authors dealt with incomplete data, and would like to see this better explained in the text.*

We assume here that the reviewer is referring to incomplete trait data here, based on their reference to the methodological constraints. Trait data could be missing in a number of different ways, but the main issues were the cases where the species naming within BioTIME may not be relatable to trait data bases, or that the species did not have trait data listed.

Our approach to incomplete alignment between the trait data and the population data was quite straightforward - we did not attempt to infer or impute any missing data and focussed on the correlation between the trait values and the population responses ($\beta$) across the species that we had data for. A possible source of confusion here is that we linked the trait data before doing the final rounds of filtering described in the methods (L186-191), so that our dataset would of the maximum use to other future users.

Where we do not have trait data, it is excluded from the calculation of the Kendall rank correlation coefficient between the trait in question and $\beta$. In the method for calculating $\beta$ that we include in the main text, each species is looked at in isolation, so the analysis proceeds as if those species were removed.

In the case of the population rank-change approach to calculating $\beta$, the counts of species that do not have trait data are included in the calculation of $\beta$. We considered this one of the advantages of the rank-abundance approach. However, the species without trait data cannot be considered in the trait-β correlation.

We have adjusted the main text (L47) and the methods section (L201) to make this more clear.

*L 54: several?*

We presume that the suggestion is to replace: '**Certain'** individual studies showed significant relationships… . It wouldn't be a big change either way, but we don't consider the number sufficient to be labelled as 'several'.

*Line 62: See my comment above on the potential influence of size range in the community values.*

We have added trait range as an additional tested co-predictor at this location, as we discuss above.

*L. 68-69: The authors should also discuss how representative the biotime is taxonomically. Most of the time series do not include large vertebrate species, which are those on which much of this size-dependent threat literature has been developed*

We have added extra text highlighting the lack of large-vertebrate coverage in the BioTIME database [L68-69]. We agree it is important to make crystal clear that our manuscript is not focussed on the threats faced by large-vertebrate apex species. There are important distinctions between the

conservation-orientated threat literature (which as the reviewer points out is primarily large and charismatic vertebrate focussed) and broader community ecology questions.

However, in terms of BioTIME's representiveness, large vertebrate species are a tiny fraction of global biodiversity (despite being a fraction with distinctive functional roles). Some would argue that BioTIME is biased *towards* vertebrates, not against them, although this is mostly towards small ones.

*l. 96 I think "spatial, temporal and TAXONOMIC BIAS" would be more appropriate than "limitations"*

We would agree that taxonomic biases within BioTIME are a significant issue, but the taxonomic biases could not be detected by the tests we refer to there. We discuss taxonomic unevenness and biases later in the paragraph. The difference is largely semantic, however at that point in the manuscript we discuss issues such as shortness of timeseries or the number of species which we feel are 'limitations' (due to the scale of data), rather than 'biases' (due to unbalanced sampling) in the data - although they might have the potential to cause biases in the results.

*L 110: I think this is the first time you mention 3 approaches. Above you only say you repeat the analysis using different transformations and cite the SI.*

Thank you for the spot. We have smoothed this out and added a pointer the results figure in SI.

*L. 224-226: cite the corresponding SI here.*

Added

*L. 248: I am not sure I understand this. At least 5 times the species occur in the time series? i.e. observed at 5 time steps?*

*Please clarify.*

Yes – that is correct. We have clarified the text (L225). The key point is that we only tested those species that were observed at least 5 times - the total time series could be longer (e.g 8,4,2,0,3,0,1).

*Also, as this is based on a continuous value, why not using that value directly instead of binarizing into winner and looser which degrades the information into a threshold-dependent binary outcome?*

We are extracting a measure in the confidence in trends here, not simply the slope as such, in order to provide a complimentary analysis to the main results. While we could use the p-value as some kind of continuous response, this does not feel particularly natural and would introduce additional distracting considerations.

*Extended Data Table 1: Revise sentence "There was no consistent drivers.."*

We have fixed the grammar here, thank you for the spot.

*I am not sure how to interpret the table. What I find confusing is the response variable. From the text (l. 62) is seems like you use the correlation coefficient, but here it seems you use a trait variable? Please clarify.*

Yes – the response variable is our 'τ'. We conduct separate analyses for each trait category. The table contains the model coefficient estimates for the effect of each possible driver on tau.

We have revised the table caption text to make this more clear:

**Extended Data Table 1** Full statistical results from lLinear model analysis of putative study-level drivers predictors of size trait – population trend correlations ($\tau$). We tested each Each trait separately, except (apart from 'Qualitative body size', for which there were too few studies for a meaningful test.) was tested independently and Nno corrections were made to the reported values for multiple comparisons. There were was no consistent drivers - while certain relationships between possible predictor variables were identified as significant at $p<0.05$ (highlighted in bold), these were not consistent across the set of traits and had low explanatory power (adjusted-$R2$) in all cases except Fish (as discussed in the main text).

*Extended data Table 4: I find hard to understand this: "relationship between the relative trait rank within an assemblage and the assignment to either 'winner' or 'loser' categories". Please clarify. In general, I think it would be really useful if you could elaborate more on some part of the methods in a dedicated section of the SI, especially the different approaches to assess trends.*

We have re-written this caption (below) to simplify the sentence structure and split the table to make it even more clear that there are two separate sets of tests being conducted. There is not much more complicated here than a series of weighted logistic regressions, that are described in the main text methods.

**Extended Data Table 4** Results from logistic regressions seeking to determine if Statistical results demonstrating the lack of a significant relationship between the relative trait rank within an assemblage and predicts the likelihood of an identifiable population level trend. Separate analyses were carried out for assignment to either 'winner' andor 'loser' categories. Each test was a logistic regression, weighted by the reciprocal of the number of cells within a study that include the species. Coefficient estimates are shown on the transformed scale. Separate models were fit for each trait and direction of trend, and no corrections were made for multiple comparisons.

*I would only provide Extended Data Table 6 as a csv table, not also in the pdf*

We previously included the large table to fulfil the requirement to directly link back to the original data providers within BioTIME. However, we agree that it was very unwieldly. To improve matters, we have greatly reduced the number of columns in the SI table, and just point out the information available in the code supplement. This has also allowed us to include data citations in a better form, and is in better keeping with other papers in this journal that have used the BioTIME dataset.

**Decision Letter, second revision:**

18th October 2021

Dear Dr. Terry,

Thank you for submitting your revised manuscript "No pervasive size trend in global community dynamics." (NATECOLEVOL-210513641B). It has now been seen again by Reviewer 3, whose are below. The reviewers find that the paper has improved in revision, and therefore we'll be happy in principle to publish it in Nature Ecology & Evolution, pending minor revisions to add further caveats in line with the reviewer's requests, and to comply with our editorial and formatting guidelines.

If the current version of your manuscript is in a PDF format, please email us a copy of the file in an editable format (Microsoft Word or LaTex)-- we can not proceed with PDFs at this stage.

We are now performing detailed checks on your paper and will send you a checklist detailing our editorial and formatting requirements in about a week. Please do not upload the final materials and make any revisions until you receive this additional information from us.

Thank you again for your interest in Nature Ecology & Evolution. Please do not hesitate to contact me if you have any questions.

***[REDACTED]***

Reviewer #3 (Remarks to the Author):

I've now read the response of the authors and the revised paper. I still think the paper is a good scientific product, although I am still not enthusiastic about the overall conclusion and message.
The bottomline of the authors is they trust "that the original compilers of BIOTIME made reasonable choices of the scale of data, that they do indeed represent meaningful communities". First, biologically speaking, those in BioTIME are not communities, but just assemblages of species that have been recorded. Second, the selection of species depends on the data used to estimate animal abundance. For example, in birds or fishes, the same sampling approach is OK to sample "most" species in an area, but this is not true for e.g. mammals, where depending on the group of species we use different techniques. This makes impossible to have e.g. time series of a mammal community. Also, for example, cetaceans are not sampled the same way fishes are sampled, so time series of fish species will unlikely include cetaceans, and yet biologically speaking cetaceans do belong to the same community and we may expect a shift due to anthropogenic effects. An honest discussion of what is a community according to BIOTIME database, and what does this imply for the conclusions, is still lacking from the text.
It is great the authors tested the effect of body size range, but one can see most of the data span 2 orders of variation (with the exception of seed mass), while just to give some examples, fishes and mammals span >6 orders of variation in body mass, and birds >4. It is not only a matter of apex predators. Fishes are almost all carnivorous, and the largest mammals and birds are not carnivorous either.
Everything in macroecology is scale-dependent, there are many factors that affect community trait shifts that act simultaneously, some are only evident over large value ranges. Provocatively, would we be able to detect the Bergmann's rule you mention within a single country? Probably not.

Overall, I remain to be convinced that traits in communities are overall shifting, or not shifting. Right now the paper sends a strong message that is appealing for big journals like Nat Ecol Evol, but may be detrimental for scientific research.
In conclusion, while the authors know acknowledge this limitation, I think this should be more apparent. Most of the conclusions are made at the beginning of the text, while this important limitation is only briefly mentioned at the end, so won't be noticed by many.
My only final comment is to be really transparent on the boundaries within which your conclusions are valid, starting from the abstract or the first part of the paper.

Our ref: NATECOLEVOL-210513641B

25th October 2021

Dear Dr. Terry,

Thank you for your patience as we've prepared the guidelines for final submission of your Nature Ecology & Evolution manuscript, "No pervasive size trend in global community dynamics." (NATECOLEVOL-210513641B). Please carefully follow the step-by-step instructions provided in the attached file, and add a response in each row of the table to indicate the changes that you have made. Please also check and comment on any additional marked-up edits we have proposed within the text. Ensuring that each point is addressed will help to ensure that your revised manuscript can be swiftly handed over to our production team.

**We would like to start working on your revised paper, with all of the requested files and forms, as soon as possible (preferably within two weeks). Please get in contact with us immediately if you anticipate it taking more than two weeks to submit these revised files.**

When you upload your final materials, please include a point-by-point response to any remaining reviewer comments.

If you have not done so already, please alert us to any related manuscripts from your group that are under consideration or in press at other journals, or are being written up for submission to other journals (see: https://www.nature.com/nature-research/editorial-policies/plagiarism#policy-on-duplicate-publication for details).

In recognition of the time and expertise our reviewers provide to Nature Ecology & Evolution's editorial process, we would like to formally acknowledge their contribution to the external peer review of your manuscript entitled "No pervasive size trend in global community dynamics.". For those reviewers who give their assent, we will be publishing their names alongside the published article.

Nature Ecology & Evolution offers a Transparent Peer Review option for new original research manuscripts submitted after December 1st, 2019. As part of this initiative, we encourage our authors to support increased transparency into the peer review process by agreeing to have the reviewer comments, author rebuttal letters, and editorial decision letters published as a Supplementary item. When you submit your final files please clearly state in your cover letter whether or not you would like to participate in this initiative. Please note that failure to state your preference will result in delays in accepting your manuscript for publication.

<b>Cover suggestions</b>

As you prepare your final files we encourage you to consider whether you have any images or illustrations that may be appropriate for use on the cover of Nature Ecology & Evolution.

Covers should be both aesthetically appealing and scientifically relevant, and should be supplied at the best quality available. Due to the prominence of these images, we do not generally select images featuring faces, children, text, graphs, schematic drawings, or collages on our covers.

We accept TIFF, JPEG, PNG or PSD file formats (a layered PSD file would be ideal), and the image should be at least 300ppi resolution (preferably 600-1200 ppi), in CMYK colour mode.

If your image is selected, we may also use it on the journal website as a banner image, and may need to make artistic alterations to fit our journal style.

Please submit your suggestions, clearly labeled, along with your final files. We'll be in touch if more information is needed.

Nature Ecology & Evolution has now transitioned to a unified Rights Collection system which will allow our Author Services team to quickly and easily collect the rights and permissions required to publish your work. Approximately 10 days after your paper is formally accepted, you will receive an email in providing you with a link to complete the grant of rights. If your paper is eligible for Open Access, our Author Services team will also be in touch regarding any additional information that may be required to arrange payment for your article.

Please note that <i>Nature Ecology & Evolution</i> is a Transformative Journal (TJ). Authors may publish their research with us through the traditional subscription access route or make their paper immediately open access through payment of an article-processing charge (APC). Authors will not be required to make a final decision about access to their article until it has been accepted. <a href="https://www.springernature.com/gp/open-research/transformative-journals"> Find out more about Transformative Journals</a>

<B>Authors may need to take specific actions to achieve <a href="https://www.springernature.com/gp/open-research/funding/policy-compliance-faqs"> compliance</a> with funder and institutional open access mandates.</b> For submissions from January 2021, if your research is supported by a funder that requires immediate open access (e.g. according to <a href="https://www.springernature.com/gp/open-research/plan-s-compliance">Plan S principles</a>) then you should select the gold OA route, and we will direct you to the compliant

route where possible. For authors selecting the subscription publication route our standard licensing terms will need to be accepted, including our <a href="https://www.springernature.com/gp/open-research/policies/journal-policies">self-archiving policies</a>. Those standard licensing terms will supersede any other terms that the author or any third party may assert apply to any version of the manuscript.

Please note that you will not receive your proofs until the publishing agreement has been received through our system.

For information regarding our different publishing models please see our <a href="https://www.springernature.com/gp/open-research/transformative-journals"> Transformative Journals </a> page. If you have any questions about costs, Open Access requirements, or our legal forms, please contact ASJournals@springernature.com.

Please use the following link for uploading these materials:
***[REDACTED]***

If you have any further questions, please feel free to contact me.

***[REDACTED]***

Reviewer #3:
Remarks to the Author:
I've now read the response of the authors and the revised paper. I still think the paper is a good scientific product, although I am still not enthusiastic about the overall conclusion and message.
The bottomline of the authors is they trust "that the original compilers of BIOTIME made reasonable choices of the scale of data, that they do indeed represent meaningful communities". First, biologically speaking, those in BioTIME are not communities, but just assemblages of species that have been recorded. Second, the selection of species depends on the data used to estimate animal abundance. For example, in birds or fishes, the same sampling approach is OK to sample "most" species in an area, but this is not true for e.g. mammals, where depending on the group of species we use different techniques. This makes impossible to have e.g. time series of a mammal community. Also, for example, cetaceans are not sampled the same way fishes are sampled, so time series of fish species will unlikely include cetaceans, and yet biologically speaking cetaceans do belong to the same community and we may expect a shift due to anthropogenic effects. An honest discussion of what is a community according to BIOTIME database, and what does this imply for the conclusions, is still lacking from the text.
It is great the authors tested the effect of body size range, but one can see most of the data span 2 orders of variation (with the exception of seed mass), while just to give some examples, fishes and mammals span >6 orders of variation in body mass, and birds >4. It is not only a matter of apex predators. Fishes are almost all carnivorous, and the largest mammals and birds are not carnivorous either.
Everything in macroecology is scale-dependent, there are many factors that affect community trait shifts that act simultaneously, some are only evident over large value ranges. Provocatively, would we be able to detect the Bergmann's rule you mention within a single country? Probably not.

Overall, I remain to be convinced that traits in communities are overall shifting, or not shifting. Right now the paper sends a strong message that is appealing for big journals like Nat Ecol Evol, but may be detrimental for scientific research.
In conclusion, while the authors know acknowledge this limitation, I think this should be more apparent. Most of the conclusions are made at the beginning of the text, while this important limitation is only briefly mentioned at the end, so won't be noticed by many.
My only final comment is to be really transparent on the boundaries within which your conclusions are valid, starting from the abstract or the first part of the paper.

**Author Rebuttal, second revision:**

*Reviewer 3.*

I've now read the response of the authors and the revised paper. I still think the paper is a good scientific product, although I am still not enthusiastic about the overall conclusion and message.

We believe the edits we have made can address the reviewer's concerns – indeed we completely agree on the importance of calibrating the strength of the conclusion appropriately. We note that our manuscript is much more circumspect and includes considerably more (both in absolute and proportional terms) discussion about the limitations for the BioTIME database than previous papers that have used the database (e.g. Dornelas et al 2014; Blowes et al 2019*;* Antão et al 2020). We also cite key papers in the extensive discussion about the use of these databases (Gonzalez et al 2016, Vellend et al 2017).

The bottomline of the authors is they trust "that the original compilers of BIOTIME made reasonable choices of the scale of data, that they do indeed represent meaningful communities". First, biologically speaking, those in BioTIME are not communities, but just assemblages of species that have been recorded. Second, the selection of species depends on the data used to estimate animal abundance. For example, in birds or fishes, the same sampling approach is OK to sample "most" species in an area, but this is not true for e.g. mammals, where depending on the group of species we use different techniques. This makes impossible to have e.g. time series of a mammal community. Also, for example, cetaceans are not sampled the same way fishes are sampled, so time series of fish species will unlikely include cetaceans, and yet biologically speaking cetaceans do belong to the same community and we may expect a shift due to anthropogenic effects. An honest discussion of what is a community according to BIOTIME database, and what does this imply for the conclusions, is still lacking from the text.

These are issues that are fundamental to all ecological research – what is a community? Although we would reject the insinuation that have been dishonest in our presentation, we have been happy to add

further clarification of what the studies in BioTIME represent. Distinctions between assemblages and communities are real, but the extent of data that the reviewer appears to be requesting here, fully complete communities, would lead to paralysis of progress in ecology. It would not be necessary to have sampled all species in a community to detect size-based determinants in performance, as long as the sampling was not strongly biased in some way.

We are speculating here, but we suspect some of these points are derived through the lens of the macro-ecological studies that attempt to link species traits to their overall global performance, in which circumstance it would be meaningful to attempt to include all mammals or birds. While related, this is a fundamentally different scale and approach to the localised community-level approach we take. Our edits to the abstract are intended to make this distinction more readily apparent.

It is great the authors tested the effect of body size range, but one can see most of the data span 2 orders of variation (with the exception of seed mass), while just to give some examples, fishes and mammals span >6 orders of variation in body mass, and birds >4. It is not only a matter of apex predators. Fishes are almost all carnivorous, and the largest mammals and birds are not carnivorous either.

While there is inevitably room for improvement of the data, we believe that the trait range is sufficient to make meaningful comparisons, and is also enough of a span for it to be reasonable to expect a differences. There is not good evidence of greater influence of traits in those studies that include a wider spread of traits, as we show in our SI. As we discussed in our previous response, it is not clear that it would necessarily be advantageous to have traits spanning 6 orders of magnitude within a single study. Comparing elephants with mice is unlikely to be particularly informative because the scales of their dynamics would be likely be poorly captured in a single study.

Everything in macroecology is scale-dependent, there are many factors that affect community trait shifts that act simultaneously, some are only evident over large value ranges. Provocatively, would we be able to detect the Bergmann's rule you mention within a single country? Probably not.

This is certainly true, and indeed a major point about our paper (e.g. our final sentence '*Community responses appear to be considerably more nuanced and localised than previously considered based on theoretical macroecological expectations*'). Our results can be seen as a pushback against extrapolating from very large scales (e.g. expectations of climate warming derived from Bergmann's rule) down to the community level, where all sorts of processes are occurring, and which result in a wide variety of size trait-driven and non-size-trait driven responses.

Overall, I remain to be convinced that traits in communities are overall shifting, or not shifting. Right now the paper sends a strong message that is appealing for big journals like Nat Ecol Evol, but may be detrimental for scientific research.

Some scepticism like this is certainly warranted. However, we maintain that our study represents a strong best effort to use the breadth of data that is available. We see our paper as a comprehensive first attempt at investigating this problem and we repeatedly highlight the gaps that remain.

In conclusion, while the authors know acknowledge this limitation, I think this should be more apparent. Most of the conclusions are made at the beginning of the text, while this important limitation is only briefly mentioned at the end, so won't be noticed by many.

My only final comment is to be really transparent on the boundaries within which your conclusions are valid, starting from the abstract or the first part of the paper.

We have added additional discussion of the limitations, and reworked the abstract (as discussed over email) to make the scale of our analyses more clear.

**Final Decision Letter:**

12th November 2021

Dear Dr Terry,

We are pleased to inform you that your Brief Communication entitled "No pervasive relationship between species size and local abundance trends", has now been accepted for publication in Nature Ecology & Evolution.

Before your manuscript is typeset, we will edit the text to ensure it conforms to house style.

Once your manuscript is typeset you will receive a link to your electronic proof via email, with a request to make any corrections as soon as possible. If you have queries at any point during the production process then please contact the production team at rjsproduction@springernature.com. Once your paper has been scheduled for online publication, the Nature press office will be in touch to confirm the details.

We have commissioned a News & Views piece to accompany the article; please note that this may add a week or so to the production timeline while we work with the News & Views author.

Acceptance of your manuscript is conditional on all authors' agreement with our publication policies (see www.nature.com/authors/policies/index.html). In particular your manuscript must not be published elsewhere and there must be no announcement of the work to any media outlet until the publication date (the day on which it is uploaded onto our web site).

Please note that <i>Nature Ecology & Evolution</i> is a Transformative Journal (TJ). Authors may publish their research with us through the traditional subscription access route or make their paper

immediately open access through payment of an article-processing charge (APC). Authors will not be required to make a final decision about access to their article until it has been accepted. <a href="https://www.springernature.com/gp/open-research/transformative-journals"> Find out more about Transformative Journals</a>

<B>Authors may need to take specific actions to achieve <a href="https://www.springernature.com/gp/open-research/funding/policy-compliance-faqs"> compliance</a> with funder and institutional open access mandates.</b> For submissions from January 2021, if your research is supported by a funder that requires immediate open access (e.g. according to <a href="https://www.springernature.com/gp/open-research/plan-s-compliance">Plan S principles</a>) then you should select the gold OA route, and we will direct you to the compliant route where possible. For authors selecting the subscription publication route our standard licensing terms will need to be accepted, including our <a href="https://www.springernature.com/gp/open-research/policies/journal-policies">self-archiving policies</a>. Those standard licensing terms will supersede any other terms that the author or any third party may assert apply to any version of the manuscript.

In approximately 10 business days you will receive an email with a link to choose the appropriate publishing options for your paper and our Author Services team will be in touch regarding any additional information that may be required.

You will not receive your proofs until the publishing agreement has been received through our system.

If you have any questions about our publishing options, costs, Open Access requirements, or our legal forms, please contact ASJournals@springernature.com

An online order form for reprints of your paper is available at <a href="https://www.nature.com/reprints/author-reprints.html">https://www.nature.com/reprints/author-reprints.html</a>. All co-authors, authors' institutions and authors' funding agencies can order reprints using the form appropriate to their geographical region.

We welcome the submission of potential cover material (including a short caption of around 40 words) related to your manuscript; suggestions should be sent to Nature Ecology & Evolution as electronic files (the image should be 300 dpi at 210 x 297 mm in either TIFF or JPEG format). Please note that such pictures should be selected more for their aesthetic appeal than for their scientific content, and that colour images work better than black and white or grayscale images. Please do not try to design a cover with the Nature Ecology & Evolution logo etc., and please do not submit composites of images related to your work. I am sure you will understand that we cannot make any promise as to whether any of your suggestions might be selected for the cover of the journal.

You can now use a single sign-on for all your accounts, view the status of all your manuscript submissions and reviews, access usage statistics for your published articles and download a record of your refereeing activity for the Nature journals.

To assist our authors in disseminating their research to the broader community, our SharedIt initiative provides you with a unique shareable link that will allow anyone (with or without a subscription) to read the published article. Recipients of the link with a subscription will also be able to download and

print the PDF.

You can generate the link yourself when you receive your article DOI by entering it here: <a href="http://authors.springernature.com/share">http://authors.springernature.com/share<a>.

***[REDACTED]***

P.S. Click on the following link if you would like to recommend Nature Ecology & Evolution to your librarian http://www.nature.com/subscriptions/recommend.html#forms

** Visit the Springer Nature Editorial and Publishing website at <a href="http://editorial-jobs.springernature.com?utm_source=ejP_NEcoE_email&utm_medium=ejP_NEcoE_email&utm_campaign=ejp_NEcoE">www.springernature.com/editorial-and-publishing-jobs</a> for more information about our career opportunities. If you have any questions please click <a href="mailto:editorial.publishing.jobs@springernature.com">here</a>.**
